# Gender disparities in access to care for time-sensitive conditions during COVID-19 pandemic in Chile

**DOI:** 10.1186/s12889-021-11838-x

**Published:** 2021-10-19

**Authors:** Jorge Pacheco, Francisca Crispi, Tania Alfaro, María Soledad Martínez, Cristóbal Cuadrado

**Affiliations:** 1 Departamento de Salud Pública, Universidad de Concepción, Víctor Lamas 1290 Casilla 160-C, 4070386 Concepción, Chile; 2 Escuela de Salud Pública, Universidad de Chile, Independencia 939, Independencia, 8380453 Santiago de Chile, Chile; 3 Centre for Health Economics, University of York, York, UK

**Keywords:** Gender, Pandemics, Health services accessibility

## Abstract

**Background:**

During the COVID-19 pandemic, reductions in healthcare utilization are reported in different contexts. Nevertheless, studies have not explored specifically gender disparities in access to healthcare in the context of COVID-19.

**Methods:**

To evaluate gender disparities in access to medical in Chile we conducted an interrupted time series analysis using segmented regression. The outcome variable was the number of weekly confirmed cases of a set of oncologic and cardiovascular time-sensitive conditions at a national level. The series contained data from weeks 1 to 39 for 2017 to 2020. The intervention period started at week 12. We selected this period because preventive interventions, such as school closures or teleworking, were implemented at this point. We estimated the level effect using a dummy variable indicating the intervention period and slope effect using a continuous variable from weeks 12 to 39. To test heterogeneity by gender and age group, we conducted a stratified analysis.

**Results:**

We observed a sizable reduction in access to care with a slowly recovery for oncologic (level effect 0.323; 95% CI 0.291–0.359; slope effect 1.022; 95% CI 1.016–1.028) and cardiovascular diseases (level effect 0.586; 95% CI 0.564–0.609; slope effect 1.009; 95% CI 1.007–1.011). Greater reduction occurred in women compared to men, particularly marked on myocardial infarction (level effect 0.595; 95% CI 0.566–0.627 versus 0.532; 95% CI 0.502–0.564) and colorectal cancer (level effect 0.295; 95% CI 0.248–0.35 versus 0.19; 95% CI 0.159–0.228). Compared to men, a greater absolute reduction was observed in women for oncologic diseases, excluding sex-specific cancer, (1352; 95% CI 743–1961) and cardiovascular diseases (1268; 95% CI 946–1590).

**Conclusion:**

We confirmed a large drop in new diagnoses for time-sensitive conditions during the COVID-19 pandemic in Chile. This reduction was greater for women. Our findings should alert policy-makers about the urgent need to integrate a gender perspective into the pandemic response.

**Supplementary Information:**

The online version contains supplementary material available at 10.1186/s12889-021-11838-x.

## Background

The COVID-19 pandemic reduced the utilization of health care services, similarly to the phenomena reported in previous epidemic outbreaks, like SARS [1], MERS [2], and Ebola [3]. In the current pandemic, studies have shown a decrease in the frequency of different interventions like surgeries (electives or not) and hospital admissions, including specific time-sensitive conditions, such as acute coronary syndrome [4, 5], myocardial infarctions [6, 7], stroke [8–11] and cancer [12–17].

Although it has been largely studied that gender impacts access to healthcare [18–20], gender differences in access to healthcare have been scarcely examined during the COVID-19 pandemic. While most studies have not explored heterogeneity by gender [4, 9–15], some studies that examine differences between men and women on acute coronary syndrome [5, 6] and stroke [8] have not found relevant disparities. Only one study was done in Latin America and did not explore gender differences [11]. To the best of our knowledge, a single research explored access differences in cancer care by gender during the pandemic. The authors did not identify any relevant differences, although the more considerable decrease was for breast cancer [17].

Gender has been proposed as a structural determinant of health, as gender norms shape social stratification, health-related exposures and behaviors, healthcare access, health systems, and health research [21]. Nevertheless, the response to outbreaks has been usually devoid of a gender perspective, limiting the effectiveness of the public health response [22, 23].

Gender norms and stratification influence social and economic outcomes, which in turn could impact access to health care [24]. First, evidence has demonstrated that school closure and mandatory confinement have increased caregiving responsibilities in families, which traditionally fall on women, producing significant disruption in their daily lives compared with men [25]. Second, as there is a general reduction in the availability of health services, gender bias that usually affects access for women, especially to cardiovascular diseases, may increase [26]. Finally, during the pandemic, employment was impacted, and many people suffered income reduction. As women are overrepresented in informal jobs, they experienced higher unemployment rates and a more significant reduction in working hours and salaries compared with men during the pandemic in different contexts [27, 28]. Also, COVID-19 has increased levels of gender violence, and reproductive health is usually not prioritized during emergencies [24], potentially reducing access to relevant diagnostic services such as smear tests for cervical cancer. Furthermore, it is important to consider in this framework the intersections that each of these areas has with other conditions such as age, socioeconomic level, ethnic background, migration status, and others, which may modify their implications [29].

This study aims to evaluate disparities between men and women in access to medical care in Chile during the COVID-19 pandemic. We focus on severe and time-sensitive group conditions (cardiovascular diseases and cancer) with guaranteed access in the context of the Chilean health system. As observed in other countries, we hypothesized a large drop in both group conditions diagnosis, but with a more significant decrease in women.

## Methods

### Study setting

In 2005 Chile implemented a Health Reform which included the National Explicit Health Guarantees Regime (“AUGE”, nowadays “GES” - explicit guarantees in health-), a set of guarantees aimed to ensure access to timely (opportunity guarantee), affordable, and quality services for people of both insurance systems predominant in Chile (public, National Health Fund - FONASA -, and private, ISAPRES), for 56 health conditions, which have been amplified to 85 nowadays [30]. During the current pandemic, the obligation for FONASA and ISAPRES to comply with the Explicit Guarantee of opportunity established for the health problems was suspended for up to 1 month since the 8th of April, except for severe conditions included in this study such as acute myocardial infarction, stroke, and cancers.

Before the onset of the pandemic, Chilean women used more healthcare services than men. They declared a worse self-perception of their health status and a greater number of healthcare needs [31]. In relation to health conditions included in GES, women have a larger waiting time than men, especially in the age group between 35 to 49 years [20]. For acute myocardial infarction, women in Chile have higher in-hospital mortality and a lower probability of receiving treatment of proven clinical efficacy compared to men [32].

### Variables conceptualization

#### Gender

Sex and gender are highly entangled, and therefore is difficult to separate them for analysis [33]. In this study, we state that the measured differences in access to healthcare by sex are explained mainly by gender norms. First, because the role of gender in access to healthcare has been previously studied as a relevant factor [18–20]. Second, because it seems less plausible that the variations between females and males in the utilization before and after the pandemic are due to biological characteristics. Therefore, and following other authors who choose the term gender to account for social and structural factors [33], hereafter the manuscript refers to “gender” for the studied categories of women and men.

#### Health care utilization

According to Levesque’s model of health care accessibility [34], we conceptualize health care utilization as the result of a dynamic interplay between individuals and services. In this model, access is defined as an opportunity to reach and obtain appropriate health care services in situations of perceived need for care. Five dimensions of health services explain accessibility: approachability, acceptability, availability and accommodation, affordability, and appropriateness. Each of these dimensions is related to an individual ability to generate access: the ability to perceive, ability to seek, ability to pay, and ability to engage. Health care access barriers (or facilitators) can appear in each dimension and occur in a cumulative manner. During the pandemic, emerging barriers decreased accessibility to health care services, affecting individuals (e.g. decrease of acceptability due to fear of contagion) and services (e. g. decrease of availability due to human resources diversion) reducing health care utilization. Such barriers can be different in type and intensity based on gender-roles.

#### Data sources

We obtained data from the National Health Fund (Fondo Nacional de Salud - FONASA) which finances all public hospitals in Chile and provides health coverage to nearly 15 million inhabitants (75% of the Chilean population). We selected a set of nine time-sensitive conditions included in the National Explicit Health Guarantees Regime (“AUGE”): two acute cardiovascular diseases (stroke and myocardial infarction) and seven cancers (gastric cancer, colorectal cancer, lymphoma, leukemia, cervical cancer, breast cancer, and testis cancer). We selected both group conditions because they encompass the two major causes of death in Chile. Additionally, delayed care for time-sensitive conditions, such as major cardiovascular events and cancer, can lead to an increased risk of long-term disability and premature death. Also, the demand for acute cardiovascular diseases is inelastic so short-term variations indicate severe disruptions in health services utilization [35].

The attending physician registers every public-insured patient with a medical diagnosis of these conditions as a confirmed case. National clinical guidelines standardize the diagnostic process for each disease, reducing practice variation and improving reporting quality. A confirmed case report is mandatory by law for healthcare providers. A description of case definitions included in the National Clinical Guidelines is available in the Supplementary File 1 (Table S1).

#### Analysis

We conducted an interrupted time-series analysis using a segmented regression [36]. Due to the count nature of the data (number of cases diagnosed per week), we fitted generalized linear models with a Negative Binomial distribution. The outcome variable was the number of confirmed cases for the following diseases: stroke (includes transient ischemic attack), myocardial infarction, all cardiovascular diseases (stroke plus myocardial infarction), gastric cancer, colorectal cancer, lymphoma, leukemia, cervical cancer (includes dysplasia), breast cancer, testicular cancer, and all cancers.

The series contained data from epidemiological weeks 1 (December 30th to January 5th) to 39 (September 21th to 27th) for the years 2017 to 2020 (156 weeks). The intervention period started at week 12 (March 16th to 22th). We selected this period because most of the public health interventions implemented during the pandemic, including school closures and remote working recommendations, started at this point (March 15th). Also, in that period started a process of cessation of elective surgeries and centralization of acute beds by the Ministry of Health. Interventions and dates details are available in the Supplementary File 1 (Table S2).

The model was defined as:

$$ Log\left({Y}_{dt}\right)= Log\left({P}_{dt}\right)+{\beta}_0+{\beta}_1 time+{\beta}_2 intervention+{\beta}_3 intervention\ast tfter+{\beta}_4 age+{Z}_{dt}+\varepsilon $$

With *Y*_*dt*_ the number of confirmed cases of disease *d* in week *t*, *P*_*dt*_ the population (number) of public health beneficiaries by age-group, *β*_1_ is the time elapsed since the start of the study (in weeks), *β*_2_ is a dummy variable indicating the intervention period (coded 1), *β*_3_ is the time elapsed since the beginning of the intervention (in weeks), *β*_4_ adjust for the effect of age (20 to 29 years, 30 to 39 years, 40 to 49 years, 50 to 59 years, 60 to 69 years, 70 to 79 years, 80 years, and more), *Z*_*dt*_ a vector of adjustment co-variable for weekly and yearly seasonal fixed effect terms and *ε* is an error term.

To test heterogeneity, we did stratified analysis by gender and gender-age. As a sensitivity analysis, we fitted unadjusted and adjusted models for all cancers, after excluding sex-specific cancers (breast cancer, cervical cancer, and testicular).

We reported the mean and standard deviation for descriptive analysis and incidence rate ratios (IRR) and absolute effects (counts) with 95% confidence intervals for regressions models. We used STATA 16.0 for analyses.

## Results

We analyzed a total of 156 weeks with 327,477 cardiovascular events (83,034 strokes and 244,443 myocardial infarction) and 137,700 cancer diagnoses (23,135 gastric cancers, 24,579 colorectal cancers, 5,290 lymphomas, 2535 leukemia, 42,143 cervical cancers, 37,443 breast cancers, and 2,575 testicular cancers) during the study period (Table S3, Supplementary File 1).

**Fig. 1:**
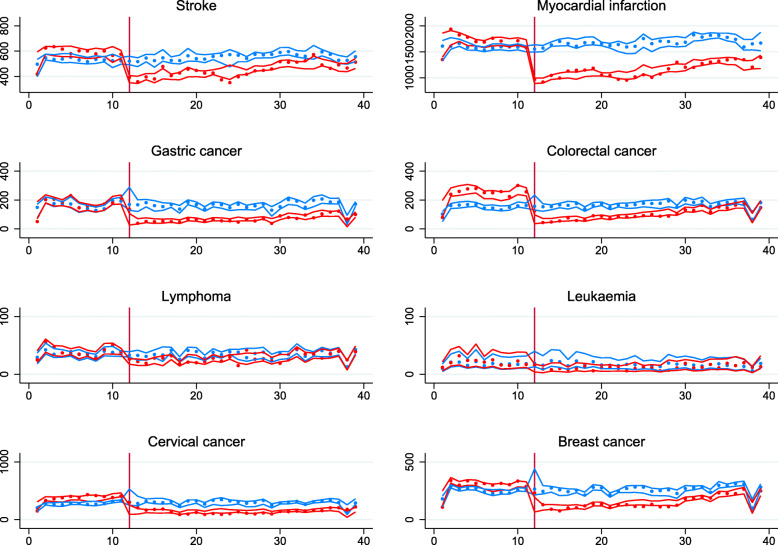
Number of new cases diagnosed per week for each disease during the first 39 weeks of the year, 2017-2020. Points represent the average number of events (new cases diagnosed) per week for each disease during the first 39 weeks of the year. Solid lines are the point estimate for the fitted model. Colored areas around the lines are the 95% confidence intervals for the fitted model. In blue, the cases observed in the years 2017–2019 (used as a control group). In green, the number of patients diagnosed in 2020 (affected by the COVID-19 pandemic). The vertical line represents the starting week of the first population-level interventions for COVID-19 in Chile (week 12)

**Fig. 2:**
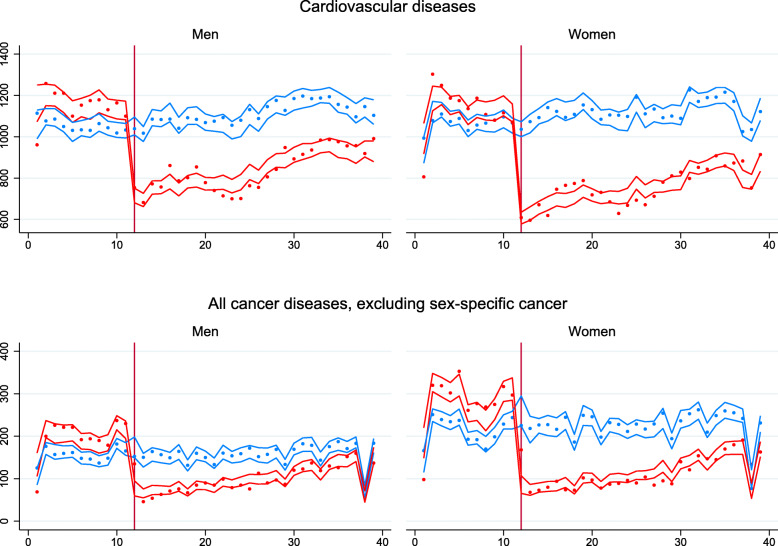
Number of new cases by sex of cardiovascular diseases and cancers, excluding sex-specific cancers, during the first 39 weeks of the year, 2017-2020. Points represent the average number of events (new cases diagnosed) per week for all cancers and cardiovascular events during the first 39 weeks of the year. Cancers exclude sex-specific conditions such as breast, cervical, or testicular cancer to facilitate comparisons between genders. Cardiovascular events include stroke and myocardial infarction. Solid lines are the point estimate for the fitted model. Colored areas around the lines are the 95% confidence intervals for the fitted model. In blue, the cases observed in the years 2017–2019 (used as a control group). In red, the number of patients diagnosed in 2020 (affected by the COVID-19 pandemic). The vertical line represents the starting week of the first population-level interventions for COVID-19 in Chile (week 12)

Compared to previous years (2017–2019), after week 12 (March 16, 2020) an immediate downward trend in the number of events was confirmed for all diseases (Fig. 1). In the oncologic diseases group, a smaller decrease occurred for lymphoma and leukemia (Fig. 1). In the cardiovascular diseases group, we observe more substantial reductions for myocardial infarction compared to stroke. Previous to the observed downward, trends were parallel for all studied diseases. The drop observed at week 38 is related to the national holiday. When analyzed by gender, women showed a greater impact on their access compared with men for both diseases groups during the study period (Fig. 2).

In our model, we confirmed a larger immediate reduction (level effect) for cancer conditions (0.323; 95% CI 0.291–0.359) compared to the cardiovascular events (0.586; 95% CI 0.564–0,609). In contrast, the post intervention slope was larger for cancer conditions (1.022; 95% CI 1.016–1.028) than cardiovascular events (1.009; 95% CI 1.007–1.011) (Table 1). Among cancer conditions, a greater immediate reduction was observed in colorectal cancer (0.229; 95% CI 0.199–0.265), gastric cancer (0.306; 95% CI 0.253–0.371), cervical cancer (0.335; 95% CI 0.287–0.392) and breast cancer (0.336; 95% CI 0.293–0.385). A greater post intervention slope was observed in colorectal cancer (1.036 95% CI 1.028– 1.043), breast cancer (1.028 95% CI 1.021–1.036) and gastric cancer (1.022 95% 1.011–1.032). This suggest a rapid recovery trend for this diagnosis after the initial abrupt reduction for this conditions. In the cardiovas- cular group, the most affected condition was myocardial infarction (0.564 95% CI 0.539–0.589) with similar post intervention trends. (Table 1).

**Table 1:** Incidence Rate Ratio for weekly confirmed cases during the pandemic period (week 12-39)^a^

	Both sexes		Men		Women	
	Level effect	Slope effect	Level effect	Slope effect	Level effect	Slope effect
All cardiovascular diseases	0.586 (0.564–0.609)	1.009 (1.007–1.011)	0.621 (0.593–0.65)	1.008 (1.005–1.01)	0.553 (0.527–0.579)	1.01 (1.008–1.012)
Stroke (includes transient ischemic attack)	0.653 (0.617–0.691)	1.008 (1.006–1.011)	0.697 0.649–0.75)	1.008 (1.005–1.012)	0.613 (0.571–0.658)	1.008 (1.005–1.012)
Myocardial infarction	0.563 (0.539–0.589)	1.009 (1.007–1.011)	0.595 (0.566–0.627)	1.007 (1.005–1.01)	0.532 (0.502–0.564)	1.011 (1.008–1.014)
All cancer	0.323 (0.291–0.359)	1.022 (1.016–1.028)	0.364 (0.315–0.42)	1.024 (1.017–1.031)	0.312 (0.279–0.35)	1.021 (1.015–1.028)
All cancer (excluding sex specific cancer)	0.293 (0.258–0.334)	1.028 (1.021–1.035)	0.351 (0.302–0.408)	1.025 (1.017–1.033)	0.254 (0.218–0.296)	1.03 (1.022–1.038)
Gastric cancer	0.306 (0.253–0.371)	1.022 (1.011–1.032)	0.338 (0.265–0.431)	1.021 (1.008–1.035)	0.228 (0.231–0.36)	1.021 (1.009–1.033)
Colorectal cancer	0.229 (0.199–0.265)	1.036 (1.028–1.043)	0.295 (0.248–0.35)	1.032 (1.023–1.041)	0.19 (0.159–0.228)	1.038 (1.029–1.048)
Lymphoma	0.569 (0.467–0.693)	1.017 (1.007–1.028)	0.643 (0.49–0.844)	1.009 (0.996–1.022)	0.497 (0.378–0.655)	1.025 (1.01–1.039)
Leukaemia	0.388 (0.286–0.526)	1.031 (1.015–1.047)	0.383 (0.251–0.586)	1.034 (1.011–1.058)	0.392 (0.259–0.594)	1.027 (1.006–1.05)
Cervical cancer (includes dysplasia)	–	–	–	–	0.335 (0.287–0.392)	1.007 (0.998–1.016)
Breast cancer	–	–	–	–	0.336 (0.293–0.385)	1.028 (1.021–1.036)
Testicular cancer	–	–	0.469 (0.339–0.649)	1.013 (0.997–1.029)	–	–

^a^Interrupted time series analysis by sex adjusted by age, population size, and seasonality (week and year). The model includes level and slope effect terms. Complete models are available in Supplemental material (Table S3-S5)

A differential impact, with larger effects on women than men, was observed across cardiovascular and oncological diseases. For the former, a 6.8% (0.621 95% CI 0.593–0.65 in men and 0.553 95% CI 0.527–0.579 in women) additional immediate reduction in access for cardiovascular events in women compared with men was evident. For the latter, a further non-significant 5.2% immediate reduction (0.364; 95% CI 0.315–0.408 in men and 0.312 95% CI 0.279–0.35) in access to newly diagnosed cancers among females compared with males was observed in the pandemic period. In the sensitivity analysis, differences between sexes in the cancer group increased after excluding sex-specific cancers such as breast, cervical, and testicular cancers. In this analysis, a bigger impact on access was confirmed among women (0.351; IC 95% 0.302–0.408 in men and 0.254; IC95% 0.218–0.296 in men). Differences in post-intervention trends were similar in both groups. (Table 1).

When analyzed by specific cardiovascular diseases, a greater immediate decrease in women than men took place for myocardial infarction (0.697; 95% CI 0.649–0.75 in men and 0.532 95% CI 0.502–0.564 in women). When analyzed by specific oncologic diseases, a greater immediate decrease in women than men occurred for colorectal cancer (0.295; 95% CI 0.248–0.35 in men and 0.19; 95% CI 0.159–0.228 in women). Also, a greater immediate reduction on cervical cancer (0–335 95% CI 0–287-0.392) and breast cancer (0.336 95% CI 0.293–0.385) compared to testicular cancer (0.469; 95% CI 0.339–0.649) was observed (Table 1). Post intervention trends were similar for all specific cardiovascular and oncologic diseases.

To make sense of these findings, we also present absolute effects sizes. A greater absolute effect in access for women compared to men occurred in almost all conditions (Table 2). An excess impact in women compared to men was observed for oncologic (9,140; 95% CI 4,619-13,661) and cardiovascular diseases (1,268; 95% CI 946–1,590) during the 28 weeks of the pandemic included in the study period. In the sensitivity analysis, differences between genders persisted but were smaller (1,352; 95% CI 743–1,916). When analyzed by specific diseases, we found sizable differences in access for women compared to men for myocardial infarction (729 95% CI 631–930), colorectal cancer (844 95% CI 288–1401), gastric cancer (562; 95% CI 362–762) and stroke (538 95% CI 250–624).

**Table 2:** Absolute reduction in confirmed cases during the pandemic period (week 12–39)

	Men Count (95%CI)	Women Count (95%CI)	Excess impact on woman Count (95%CI)
All cardiovascular diseases	9,047 (6,845 - 11,248)	10,315 (7,791 - 12,838)	1,268 (946–1,590)
Stroke (includes transient ischemic attack)	1,557 (798–2,214)	2,286 (1,428–3,144)	729 (631–930)
Myocardial infarction	7,497 (5,702 - 8,906)	8,035 (5,952 - 9,529)	538 (250–624)
All cancer	2,056 (611–3,161)	11,196 (5,229 - 17,163)	9,140 (4,619 - 13,661)
All cancer (excluding sex specific cancer)	1,863 (564–3,161)	3,215 (1,307 - 5,122)	1,352 (743–1,961)
Gastric cancer	828 (44–1,612)	1,390 (406–2,374)	562 (362–762)
Colorectal cancer	896 (348–1,444)	1,740 (636–2,844)	844 (288–1,401)
Lymphoma	128 (− 25–281)	111 (− 36–258)	17 (11–23)
Leukemia	10 (− 20–40)	15 (− 44–13)	-5 (−4 - -7)
Cervical cancer (includes dysplasia)	..	5,185 (2,522 - 7,848)	..
Breast cancer	..	2,931 (784–5,078)	..
Testicular cancer	202 (−25–430)	..	..

In our final analysis, we estimated relative and absolute effects across gender and age groups for cardiovascular diseases and oncologic diseases, excluding sex-specific cancer (Table S4﻿). For cardiovascular diseases, we only observed a significant immediate decrease for the 20 to 29 years’ group. A larger absolute difference for women compared to men was observed in the older groups (527 95% CI 485–569 in the 70 to 79 years’ group and 668 95% CI 472–614 in the 80 years and older group). For oncologic diseases, a larger immediate decrease was evident for women in all ages groups, and the greater absolute difference was observed among middle-aged and older women (426 95% CI 175–676 in 50 to 59 years’ group and 395 95% CI 364–427 in 60 to 69 years group).

## Discussion

Our analysis confirmed a large drop in the access to medical diagnosis for cardiovascular and oncologic conditions in Chile during COVID-19 pandemic as previous studies have shown [4–15], This decrease was more significant for oncologic than cardiovascular diseases. Also, we confirmed our hypothesis of sizable gender disparities in the impact of the pandemic on access to medical care. A large group of time-sensitive conditions was affected by this differential effect, even though healthcare access for these conditions is guaranteed by law in Chile. This finding is worrisome because delaying care for these severe conditions can lead to long-term disability and - eventually - premature mortality.

Because cardiovascular diseases and cancers have different etiological mechanisms, it is highly implausible to explain this finding through biological mechanisms. While a stroke and myocardial infarction could increase after COVID-19 infection [37, 38], a reduction in the number of diagnosed cardiovascular events is probably explained by decreased access to healthcare. If men are more prone to COVID-19 [39], this could explain, at least partially, that the decline in stroke and myocardial infarction in males could be smaller compared with women. Although, this explanation cannot be given in cancer because these diseases do not share the same causes and acute changes in cancer incidence are unlikely to be attributable to COVID-19 infection. In this setting, a reduced number of newly diagnosed cancers, particularly among women, is a clear marker of reduced access and unmet needs.

Gender norms and hierarchies could explain this wide effect better. During the pandemic, women faced more health care barriers than men. Income decrease due to work hours reduction [27] and higher unemployment [28] reduced women’s ability to pay for health care. Also, interventions to reduce COVID-19 transmission, such as school closures, increased the care burden in families reducing women’s time availability to seek care [25]. This could explain the greater differential effect observed on diseases that require scheduled appointments for testing (e.g., colorectal, cervical, gastric, and breast cancer).

From a health services perspective, the diversion of resources (health personnel, hospital beds, among others) to cope with the pandemic reduced provider’s availability for cancer and cardiovascular care. Previous to the pandemic, women waited more time than men to access care for these health conditions [20]. For acute myocardial infarction, the treatment of women was proven suboptimal compared to men [32]. These gender biases could be aggravated in the context of health services scarcity, differentially affecting the ability to reach and use health care services in women [21].

Finally, fear of SARS-CoV-2 contagion in medical settings could reduce the acceptability of health services, decreasing the user’s ability to seek care. The reduction of health services utilization showed a rapid onset starting when the first control measures were established. This sudden decrease preceded the stay-at-home mandates (March 26) and lockdowns (May 13). Also, it preceded the period of the highest incidence of cases (May–June). This pattern could be explained by user fear triggered by extensive media coverage about death overseas and the uncertainty of a new infectious disease during the first weeks of the pandemic. A Chilean survey evidenced that this fear was more frequent in women than in men [40]. It is unclear why women suffered more fear but this could partly explain immediate differences in access to health care.

Women’s health access disruption has been observed for other conditions. A recent systematic review concluded that maternal and fetal outcomes worsened during the COVID-19 pandemic with an increase in maternal deaths, stillbirth, ruptured ectopic pregnancies, and maternal depression [41]. Similar disruptions were observed for contraception and safe abortion services [24]. According to these findings, an urgent call to protect sexual and reproductive care and to include a gender perspective in the pandemic response was raised by multiple humanitarian organizations [42].

As strengths, this is the first study from Latin America that explores access by gender to medical diagnosis during the COVID-19 pandemic. To test our hypothesis, we used a rich, comprehensive, and reliable national database where cases were defined based on standardized diagnostic processes. We select a variety of severe time-sensitive conditions to avoid generalization based on anecdotal evidence. Moreover, we tested different models, maintaining our conclusions unchanged.

This study has several limitations. First, we use administrative data, which might be subject to underreporting during the pandemic. Nevertheless, confirmed case reports have been mandatory for healthcare providers since 2004. Moreover, they are used for health claim payments in the Chilean health system, therefore making it less likely that reduced reporting could explain the observed effect. Second, due to the data codification, this study only considers two categories for sex and gender (female and male). This dichotomy excludes a spectrum of gender identities and the intersex population [21]. Future studies must explore differential effects on health care accessibility during pandemics for broader gender classification. Third, we cannot rule out residual confounding in the context of observational data. Nevertheless, due to the characteristic of the exposure of interest (the pandemic) is unlikely that better data could be obtained using alternative sources or study designs. We controlled confirmed cases by population and age in our models and included seasonal adjustments by week and year to control for unobserved time-specific confounding factors. The use of previous year trends as a control for the same observational units allows adjustment for confounding, but since no parallel control group was available adjustment for other time-variant effects concomitants to the pandemic was not feasible.

## Conclusion

In our study, we confirmed a large drop in the medical diagnosis for time-sensitive conditions during the COVID-19 pandemic in Chile. Additionally, we demonstrate that women were far more affected compared to men. This differential effect by gender was observed for a broad group of time-sensitive conditions. As researchers have posed [22, 23], our findings should alert policy-makers about the urgent need to integrate a gender perspective into outbreak response. If school closure has a role in the observed differential effect, increasing the number of health care services will not be enough to shorten these disparities between genders. Services provision should be reachable, especially for women who are raising children or have other caregiver responsibilities and reduce economic barriers. Also, health professionals should be aware of this situation and encouraged through clinical guidelines to reduce current gender bias in their clinical practice.

Future research must evaluate the consequences of access reductions on disability and premature death. The observed effect occurred in a set of severe time-sensitive conditions where care delays could worsen prognosis. Additionally, we need to know the causes, which could be informed through surveys and innovative ways to provide care for these diseases during the actual pandemic.

## Supplementary Information

**Additional file 1.**

## Data Availability

The datasets supporting the conclusions of this article are available in the GitHub repository: https://github.com/CoV-IMPACT-C/gender-impact-access-covid

## Abbreviations

AUGE: Acceso Universal a Garantías Explícitas (National Explicit Health Guarantees Regime)
COVID-19: Coronavirus disease 2019
FONASA: Fondo Nacional de Salud (Public insurer)
IRR: Incidence Rate Ratio
ISAPRES: Instituciones de Salud Previsional (Private insurers)
MERS: Middle East respiratory syndrome
SARS: Severe acute respiratory syndrome
