# Supplementary material for: Gender disparities in access to care for time-sensitive conditions during COVID-19 pandemic in Chile
Source: BMC Public Health. 2021 Oct 19;21:1802. doi: 10.1186/s12889-021-11838-x (PMC8523204; doi:10.1186/s12889-021-11838-x)
Supplement: Supplementary file 1 — Additional file 1. [file 12889_2021_11838_MOESM1_ESM.docx]

**Supplementary File**

**Table S1.- Case definition included in the National Clinical Guidelines**

| **Health problem** | **Medical diagnosis included ICD-10** |
| --- | --- |
| **Stroke** | Stroke (I63, I64) and transient ischemic attack (G45) |
| **Myocardial infarction** | Myocardial infarction (I21-I22) |
| **Gastric cancer** | Gastric cancer (C16) and Carcinoma in situ of stomach (D00.2) |
| **Colorectal cancer** | Malignant neoplasm of colon (C18), rectosigmoid junction (C19) and rectum (C20) |
| **Lymphoma** | Hodgkin lymphoma (C81), Follicular lymphoma (C82), Non-follicular lymphoma (C83), Mature T/NK-cell lymphomas (C84), and other specified and unspecified types of non-Hodgkin lymphoma (C85) |
| **Leukaemia** | Lymphoid leukaemia (C91), Myeloid leukaemia (C92), Monocytic leukaemia (C93), Other leukaemias of specified cell type (C94) and Leukemia of unspecified cell type (C95) |
| **Cervical cancer** | Malignant neoplasm of cervix uteri (C53), Dysplasia of cervix uteri (N87), and carcinoma in situ of cervix uteri (D06) |
| **Breast cancer** | Malignant neoplasm of the breast (C50) and carcinoma in situ of the breast (D05). |
| **Testis cancer** | Malignant neoplasm of testis (C62) |

**Table S2. Description of governmental COVID-19 control measures by date**

| **Date** | **Control measures** |
| --- | --- |
| **March 3^rd^** | First confirmed COVID case |
| **March 13^th^** | Isolation of confirmed cases in schools and preventive quarantine for contacts  Public employees over 75 years old, or who have a medical condition, will be able to work from home  Public events restrictions (<500 persons)  Border control: mandatory quarantine for people arriving from high-risk countries. |
| **March 15^th^** | Schools closure (nursery schools, municipal, subsidised private, and private schools)  Recommended remote work for non-essential workers.  Restricted access to long-term care facilities.  Day care centres and community centres for seniors are suspended.  Public events restrictions (<200 persons).  Hygiene measures reinforced in transportation.  Border control: cruise ships landfall prohibition |
| **March 18^th^** | State of catastrophe declaration.  Border control: border closure for foreigners and mandatory quarantine for Chilean nationals and permanent residents. |
| **March 20^th^** | Preventive lockdown in three sectors of the capital. |
| **March 22^nd^** | Nationwide night-time curfew |
| **March 26^th^** | Lockdown in 7 cities (1.3 million inhabitants) |
| **March 28^th^** | Lockdown in Temuco and Padre Las Casas |
| **March 30^th^** | Lockdown in Chillán, Chillán Viejo and Osorno |
| **April 1^st^** | Lockdown in Punta Arenas and Eastern Island. |
| **April 9^th^** | Suspension of maximum waiting time in some diseases included in the Universal Access Plan |

**Table S3.- Weekly number of confirmed cases by disease and year**

|  | 2017 | 2018 | 2019 | 2020 |
| --- | --- | --- | --- | --- |
|  | Mean (SD) | Mean (SD) | Mean (SD) | Mean (SD) |
| Stroke (includes transient ischemic attack) | 500,3 (34,7) | 544,4 (32,9) | 598,6 (42,2) | 485,7 (82,6) |
| Myocardial infarction | 1577,8 (105,5) | 1633,2 (86,3) | 1753,5 (113,5) | 1303,2 (287,5) |
| Gastric cancer | 158,5 (29,5) | 164,8 (31,7) | 169,9 (40,4) | 100,0 (57,0) |
| Colorectal cancer | 114,4 (18,9) | 155,8 (27,8) | 217,4 (39,4) | 142,6 (77,2) |
| Lymphoma | 32,3 (6,5) | 33 (8,2) | 39,4 (8,4) | 31,0 (9,6) |
| Leukaemia | 16,3 (4,5) | 15,8 (4,6) | 18,7 (5,3) | 14,2 (6,1) |
| Cervical cancer (includes dysplasia) | 266,5 (39,9) | 292,5 (49,7) | 312,6 (48,9) | 209,0 (116,2) |
| Breast cancer | 237,0 (33,4) | 254,5 (46,1) | 275,3 (46,1) | 193,3 (85,5) |
| Testicular cancer | 16,7 (5,0) | 16,0 (4,3) | 19 (4,7) | 14,4 (5,8) |

**Table S4.- Relative and absolute effect by gender and age group for cardiovascular and oncologic diseases (excluding sex-specific cancer)**

|  | All cardiovascular diseases | | | | |
| --- | --- | --- | --- | --- | --- |
|  | Level effect  Men  IRR (95%CI) | Level effect  Women  IRR (95%CI) | Men  Count  (95% CI) | Women  Count  (95% CI) | Excess impact on woman  Count (95%CI) |
| 20 to 29 years | 0,560  (0,506-0,621) | 0,58  (0,501-0,672) | 944  (633-1255) | 649  (271-1027) | -295  (-228 - -362) |
| 30 to 39 years | 0,621  (0,558-0,692) | 0,559  (0,488-0,639) | 882  (481-1283) | 929  (475-1384) | 47  (-6 – 101) |
| 40 to 49 years | 0,645  (0,587-0,709) | 0,528  (0,475-0,587) | 1061  (563-1560) | 1268  (740-1796) | 207  (178-236) |
| 50 to 59 years | 0,643  (0,593-0,706) | 0,567  (0,518-0,62) | 1700  (1058-2341) | 1782  (1126-2438) | 82  (69-96) |
| 60 to 69 years | 0,654  (0,604-0,706) | 0,592  (0,535-0,655) | 1576  (919-2233) | 1661  (979-2344) | 85  (59-111) |
| 70 to 79 years | 0,585  (0,539-0,635) | 0,547  (0,499-0,598) | 1621  (1031-2211) | 2148  (1516-2780) | 527  (485-569) |
| 80 years and older | 0,616  (0,551-0-688) | 0,51  (0,459-0,567) | 1241  (688-2044) | 1909  (1160-2658) | 668  (472-614) |
|  | Oncologic diseases, excluding sex-specific cancer | | | | |
|  | Level effect  Men  IRR (95%CI) | Level effect  Women  IRR (95%CI) | Men  Count  (95% CI) | Women  Count  (95% CI) | Excess impact on woman  Count (95%CI) |
| 20 to 29 years | 0,702  (0,44-1,12) | 0,303  (0,169-0,545) | 30  (-36 – 97) | 32  (-37 – 101) | 2  (-1–4) |
| 30 to 39 years | 0,43  (0,257-0,72) | 0,253  (0,154-0,417) | 59  (-26– 330) | 85  (-8 – 177) | 25  (-32-153) |
| 40 to 49 years | 0,328  (0,238-0,452) | 0,274  (0,181-0,415) | 223  (37-411) | 410  (10-810) | 187  (-26–399) |
| 50 to 59 years | 0,348  (0,273-0,443) | 0,239  (0,177-0,323) | 366  (105-628) | 792  (280-1304) | 426  (175-676) |
| 60 to 69 years | 0,361  (0,254-0,512) | 0,259  (0,197-0,342) | 498  (4-1000) | 893  (360-1426) | 395  (364-427) |
| 70 to 79 years | 0,32  (0,247-0,414) | 0,245  (0,162-0,37) | 467  (73-861) | 743  (45-1440) | 276  (-27–579) |
| 80 years and older | 0,342  (0,229-0,51) | 0,25  (0,189-0,331) | 256  (-17 – 528) | 331  (49-614) | 76  (65-86) |

Complete models are available in tables S33-S53

**Table S5.- All cardiovascular diseases model for both sexes (full specification)**

| Negative binomial regression Number of obs = | 1,092 |
| --- | --- |
| Wald chi2(49) = | 108566.15 |
| Dispersion = mean Prob > chi2 = | 0.0000 |
| Log pseudolikelihood = -4916.5618 Pseudo R2 = | 0.3436 |
|  |  |
| Robust |  |
| _freq IRR Std. Err. z P>z [95% Conf. | Interval] |
|  |  |
| t2 1.000366 .0000821 4.46 0.000 1.000205 | 1.000527 |
| 1.level .5860784 .0112538 -27.83 0.000 .5644312 | .6085558 |
| slope 1.008946 .0008506 10.56 0.000 1.00728 | 1.010614 |
| age_group |  |
| 2 1.417897 .0169431 29.22 0.000 1.385075 | 1.451496 |
| 3 2.13635 .0238885 67.89 0.000 2.090039 | 2.183687 |
| 4 3.16191 .0341329 106.64 0.000 3.095713 | 3.229522 |
| 5 4.893497 .0523418 148.46 0.000 4.791977 | 4.997168 |
| 6 7.494821 .0810211 186.32 0.000 7.337693 | 7.655314 |
| 7 10.39533 .1189116 204.68 0.000 10.16486 | 10.63102 |
| week |  |
| 2 1.105684 .03783 2.94 0.003 1.03397 | 1.182371 |
| 3 1.111321 .0374155 3.14 0.002 1.040355 | 1.187127 |
| 4 1.07181 .0356666 2.08 0.037 1.004135 | 1.144045 |
| 5 1.054568 .0355049 1.58 0.115 .9872255 | 1.126503 |
| 6 1.036034 .0350831 1.05 0.296 .9695045 | 1.107129 |
| 7 1.051834 .0358067 1.48 0.138 .983944 | 1.124408 |
| 8 1.059886 .0360264 1.71 0.087 .9915766 | 1.132902 |
| 9 1.045697 .0349918 1.34 0.182 .9793151 | 1.116579 |
| 10 1.051512 .0356758 1.48 0.139 .9838633 | 1.123813 |
| 11 1.031975 .0342923 0.95 0.344 .966905 | 1.101424 |
| 12 1.03453 .034188 1.03 0.304 .9696472 | 1.103755 |
| 13 1.027053 .0349545 0.78 0.433 .9607783 | 1.097899 |
| 14 1.084462 .0356669 2.47 0.014 1.016762 | 1.15667 |
| 15 1.090522 .0361481 2.61 0.009 1.021926 | 1.163723 |
| 16 1.118213 .0375794 3.32 0.001 1.046932 | 1.194347 |
| 17 1.073719 .0354398 2.15 0.031 1.006457 | 1.145476 |
| 18 1.101556 .0371216 2.87 0.004 1.03115 | 1.17677 |
| 19 1.118296 .0375984 3.33 0.001 1.04698 | 1.194469 |
| 20 1.080441 .035486 2.36 0.018 1.013081 | 1.15228 |
| 21 1.057598 .0343459 1.72 0.085 .9923785 | 1.127103 |
| 22 1.056516 .0359425 1.62 0.106 .9883672 | 1.129364 |
| 23 1.031478 .0358689 0.89 0.373 .9635186 | 1.104231 |
| 24 1.041165 .0377534 1.11 0.266 .9697376 | 1.117853 |
| 25 1.108627 .0383088 2.98 0.003 1.036029 | 1.186312 |
| 26 1.052648 .0355591 1.52 0.129 .9852105 | 1.124702 |
| 27 1.093491 .0363138 2.69 0.007 1.024584 | 1.167032 |
| 28 1.099993 .0362971 2.89 0.004 1.031103 | 1.173485 |
| 29 1.099815 .0385129 2.72 0.007 1.026863 | 1.17795 |
| 30 1.107772 .0378138 3.00 0.003 1.036083 | 1.184421 |
| 31 1.161217 .0387198 4.48 0.000 1.087754 | 1.239641 |
| 32 1.149154 .0370827 4.31 0.000 1.078724 | 1.224183 |
| 33 1.154638 .0369597 4.49 0.000 1.084424 | 1.229399 |
| 34 1.163076 .0382241 4.60 0.000 1.09052 | 1.240459 |
| 35 1.145778 .0383116 4.07 0.000 1.073096 | 1.223382 |
| 36 1.121912 .0371369 3.48 0.001 1.051436 | 1.197112 |
| 37 1.049309 .0366556 1.38 0.168 .9798699 | 1.123669 |
| 38 1.048259 .0356665 1.39 0.166 .9806336 | 1.120547 |
| 39 1.086335 .0405266 2.22 0.026 1.009739 | 1.168742 |
| year |  |
| 2018 1.000594 .0066296 0.09 0.929 .9876846 | 1.013673 |
| 2019 1.033601 .0093616 3.65 0.000 1.015414 | 1.052113 |
| 2020 1 (omitted) |  |
| _cons .0000695 2.21e-06 -301.10 0.000 .0000653 | .0000739 |
| ln(popula~n) 1 (exposure) |  |
|  |  |
| /lnalpha -6.147537 .1466585 -6.434982 | -5.860092 |
|  |  |
| alpha .0021387 .0003137 .0016044 | .002851 |
|  |  |
| Note: Estimates are transformed only in the first equation. |  |
| Note: _cons estimates baseline incidence rate. |  |
| . |  |

**Table S6.- All cardiovascular diseases model for men (full specification)**

| Negative binomial regression Number of obs = | 1,092 |
| --- | --- |
| Wald chi2(49) = | 62026.06 |
| Dispersion = mean Prob > chi2 = | 0.0000 |
| Log pseudolikelihood = -4429.6208 Pseudo R2 = | 0.3427 |
|  |  |
| Robust |  |
| _freq IRR Std. Err. z P>z [95% Conf. | Interval] |
|  |  |
| t2 1.000456 .0001015 4.49 0.000 1.000257 | 1.000655 |
| 1.level .6210102 .014388 -20.56 0.000 .593441 | .6498602 |
| slope 1.007514 .0010578 7.13 0.000 1.005442 | 1.009589 |
| age_group |  |
| 2 1.380245 .0206824 21.51 0.000 1.340298 | 1.421383 |
| 3 1.955802 .0267675 49.01 0.000 1.904036 | 2.008975 |
| 4 3.011683 .0386372 85.94 0.000 2.936899 | 3.08837 |
| 5 4.929564 .0631239 124.58 0.000 4.807384 | 5.054851 |
| 6 7.365361 .095675 153.72 0.000 7.180209 | 7.555288 |
| 7 10.02923 .1424872 162.28 0.000 9.753815 | 10.31243 |
| week |  |
| 2 1.03428 .0384054 0.91 0.364 .9616809 | 1.11236 |
| 3 1.030464 .0388607 0.80 0.426 .9570455 | 1.109515 |
| 4 1.001482 .0375059 0.04 0.968 .930605 | 1.077757 |
| 5 .9621518 .0377826 -0.98 0.326 .8908772 | 1.039129 |
| 6 .9783401 .0360517 -0.59 0.552 .9101713 | 1.051614 |
| 7 .9786014 .0386851 -0.55 0.584 .9056429 | 1.057437 |
| 8 1.002334 .0383701 0.06 0.951 .9298817 | 1.080431 |
| 9 .975493 .0352749 -0.69 0.493 .9087487 | 1.04714 |
| 10 .9667194 .0357618 -0.91 0.360 .8991082 | 1.039415 |
| 11 .9622113 .0354278 -1.05 0.295 .8952202 | 1.034215 |
| 12 .982831 .0371292 -0.46 0.647 .9126879 | 1.058365 |
| 13 .9452798 .0343157 -1.55 0.121 .8803593 | 1.014988 |
| 14 1.018621 .0371408 0.51 0.613 .9483669 | 1.09408 |
| 15 1.012079 .0367357 0.33 0.741 .94258 | 1.086703 |
| 16 1.040133 .0392617 1.04 0.297 .9659591 | 1.120003 |
| 17 .9811625 .0360102 -0.52 0.604 .9130624 | 1.054342 |
| 18 1.021532 .0374939 0.58 0.562 .9506267 | 1.097727 |
| 19 1.023357 .0380656 0.62 0.535 .9514046 | 1.100751 |
| 20 .9925887 .0364834 -0.20 0.840 .9235973 | 1.066734 |
| 21 .9837608 .0353538 -0.46 0.649 .9168526 | 1.055552 |
| 22 .9852871 .0373902 -0.39 0.696 .9146626 | 1.061365 |
| 23 .9533863 .0361668 -1.26 0.208 .8850717 | 1.026974 |
| 24 .9695031 .0384021 -0.78 0.434 .8970839 | 1.047769 |
| 25 1.022335 .0398203 0.57 0.571 .947193 | 1.103437 |
| 26 .9849479 .0370155 -0.40 0.687 .9150063 | 1.060236 |
| 27 1.038285 .0384017 1.02 0.310 .9656821 | 1.116346 |
| 28 1.064065 .0388407 1.70 0.089 .9905981 | 1.142981 |
| 29 1.055177 .0395936 1.43 0.152 .9803598 | 1.135704 |
| 30 1.078703 .0406146 2.01 0.044 1.001966 | 1.161317 |
| 31 1.088317 .0411412 2.24 0.025 1.010597 | 1.172015 |
| 32 1.087189 .0391072 2.32 0.020 1.013179 | 1.166604 |
| 33 1.096002 .0380827 2.64 0.008 1.023846 | 1.173243 |
| 34 1.097926 .0394033 2.60 0.009 1.02335 | 1.177936 |
| 35 1.066679 .0409169 1.68 0.092 .9894241 | 1.149966 |
| 36 1.049403 .0384579 1.32 0.188 .9766702 | 1.127552 |
| 37 1.016064 .0392678 0.41 0.680 .9419428 | 1.096017 |
| 38 1.040689 .039891 1.04 0.298 .9653688 | 1.121886 |
| 39 1.019208 .0428602 0.45 0.651 .9385722 | 1.106771 |
| year |  |
| 2018 .9988682 .0084721 -0.13 0.894 .9824004 | 1.015612 |
| 2019 1.015142 .0114719 1.33 0.184 .992905 | 1.037878 |
| 2020 1 (omitted) |  |
| _cons .0000859 2.96e-06 -271.76 0.000 .0000803 | .000092 |
| ln(popula~n) 1 (exposure) |  |
|  |  |
| /lnalpha -6.054788 .1950949 -6.437167 | -5.672409 |
|  |  |
| alpha .0023466 .0004578 .0016009 | .0034396 |
|  |  |
| Note: Estimates are transformed only in the first equation. |  |
| Note: _cons estimates baseline incidence rate. |  |

**Table S7.- All cardiovascular diseases model for women (full specification)**

| Negative binomial regression Number of obs = | 1,092 |
| --- | --- |
| Wald chi2(49) = | 71669.01 |
| Dispersion = mean Prob > chi2 = | 0.0000 |
| Log pseudolikelihood = -4391.6102 Pseudo R2 = | 0.3486 |
|  |  |
| Robust |  |
| _freq IRR Std. Err. z P>z [95% Conf. | Interval] |
|  |  |
| t2 1.000252 .0000916 2.75 0.006 1.000072 | 1.000431 |
| 1.level .5526783 .0132278 -24.78 0.000 .5273509 | .5792221 |
| slope 1.010442 .0011615 9.04 0.000 1.008168 | 1.012721 |
| age_group |  |
| 2 1.479696 .0223066 25.99 0.000 1.436615 | 1.524068 |
| 3 2.361985 .0338524 59.97 0.000 2.296559 | 2.429275 |
| 4 3.346716 .0459974 87.89 0.000 3.257766 | 3.438094 |
| 5 4.883656 .0674576 114.81 0.000 4.753215 | 5.017677 |
| 6 7.804917 .1067763 150.19 0.000 7.598421 | 8.017026 |
| 7 11.3523 .16141 170.87 0.000 11.04031 | 11.67311 |
| week |  |
| 2 1.195755 .0486599 4.39 0.000 1.104088 | 1.295033 |
| 3 1.207348 .0441998 5.15 0.000 1.123753 | 1.297162 |
| 4 1.159597 .0431049 3.98 0.000 1.078117 | 1.247235 |
| 5 1.168033 .0427476 4.24 0.000 1.087184 | 1.254895 |
| 6 1.108593 .0424827 2.69 0.007 1.028378 | 1.195064 |
| 7 1.141348 .0425282 3.55 0.000 1.060965 | 1.227821 |
| 8 1.13002 .0424284 3.26 0.001 1.049848 | 1.216315 |
| 9 1.13171 .0439005 3.19 0.001 1.048857 | 1.221109 |
| 10 1.153704 .0446065 3.70 0.000 1.069507 | 1.244529 |
| 11 1.118082 .0418187 2.98 0.003 1.039051 | 1.203124 |
| 12 1.098913 .0407596 2.54 0.011 1.021861 | 1.181776 |
| 13 1.126996 .044068 3.06 0.002 1.043851 | 1.216764 |
| 14 1.163817 .0430363 4.10 0.000 1.082451 | 1.251298 |
| 15 1.187329 .0460122 4.43 0.000 1.100487 | 1.281025 |
| 16 1.208801 .0452759 5.06 0.000 1.123241 | 1.300879 |
| 17 1.186378 .0456278 4.44 0.000 1.100236 | 1.279263 |
| 18 1.197854 .0449418 4.81 0.000 1.11293 | 1.289258 |
| 19 1.236013 .0457367 5.73 0.000 1.149544 | 1.328986 |
| 20 1.192034 .0456133 4.59 0.000 1.105904 | 1.284872 |
| 21 1.151907 .0422237 3.86 0.000 1.072053 | 1.237709 |
| 22 1.151594 .0431238 3.77 0.000 1.0701 | 1.239294 |
| 23 1.131653 .0436734 3.20 0.001 1.049212 | 1.220572 |
| 24 1.135218 .0463378 3.11 0.002 1.047936 | 1.22977 |
| 25 1.219781 .0459869 5.27 0.000 1.132898 | 1.313327 |
| 26 1.141945 .0444236 3.41 0.001 1.058113 | 1.232419 |
| 27 1.168522 .0444309 4.10 0.000 1.084605 | 1.258932 |
| 28 1.151478 .0423565 3.83 0.000 1.071383 | 1.237561 |
| 29 1.162531 .0448495 3.90 0.000 1.077869 | 1.253843 |
| 30 1.154571 .0450549 3.68 0.000 1.069557 | 1.246342 |
| 31 1.258357 .0458492 6.31 0.000 1.171628 | 1.351506 |
| 32 1.227854 .0442427 5.70 0.000 1.144131 | 1.317703 |
| 33 1.233555 .0444241 5.83 0.000 1.149487 | 1.323771 |
| 34 1.250492 .0459178 6.09 0.000 1.163657 | 1.343807 |
| 35 1.25081 .0457735 6.12 0.000 1.164238 | 1.34382 |
| 36 1.217585 .0463023 5.18 0.000 1.130134 | 1.311803 |
| 37 1.095136 .0443992 2.24 0.025 1.011483 | 1.185708 |
| 38 1.063088 .0420964 1.54 0.122 .9837015 | 1.148882 |
| 39 1.176651 .0482343 3.97 0.000 1.085812 | 1.27509 |
| year |  |
| 2018 1.004178 .0080684 0.52 0.604 .9884883 | 1.020117 |
| 2019 1.053413 .0106225 5.16 0.000 1.032798 | 1.07444 |
| 2020 1 (omitted) |  |
| _cons .0000553 1.97e-06 -274.71 0.000 .0000515 | .0000593 |
| ln(popula~n) 1 (exposure) |  |
|  |  |
| /lnalpha -6.313167 .2263595 -6.756823 | -5.86951 |
|  |  |
| alpha .0018123 .0004102 .0011629 | .0028243 |
|  |  |
| Note: Estimates are transformed only in the first equation. |  |
| Note: _cons estimates baseline incidence rate. |  |
| . |  |

**Table S8.- Stroke model for both sexes (full specification)**

| Negative binomial regression Number of obs = | 1,092 |
| --- | --- |
| Wald chi2(49) = | 71364.26 |
| Dispersion = mean Prob > chi2 = | 0.0000 |
| Log pseudolikelihood = -3839.5825 Pseudo R2 = | 0.3855 |
|  |  |
| Robust |  |
| _freq IRR Std. Err. z P>z [95% Conf. | Interval] |
|  |  |
| t2 1.000685 .000123 5.57 0.000 1.000444 | 1.000926 |
| 1.level .6528775 .0187008 -14.89 0.000 .6172346 | .6905787 |
| slope 1.008484 .001314 6.48 0.000 1.005912 | 1.011063 |
| age_group |  |
| 2 1.948597 .06094 21.33 0.000 1.832744 | 2.071774 |
| 3 4.258176 .1205342 51.18 0.000 4.028367 | 4.501094 |
| 4 8.429991 .2252346 79.79 0.000 7.999899 | 8.883206 |
| 5 16.41181 .4352529 105.50 0.000 15.58052 | 17.28745 |
| 6 29.80876 .7884719 128.34 0.000 28.30276 | 31.3949 |
| 7 48.36057 1.284556 146.02 0.000 45.9073 | 50.94494 |
| week |  |
| 2 1.189243 .058276 3.54 0.000 1.080337 | 1.309126 |
| 3 1.173301 .0536972 3.49 0.000 1.072639 | 1.28341 |
| 4 1.159406 .055104 3.11 0.002 1.056282 | 1.272598 |
| 5 1.165006 .0569476 3.12 0.002 1.058571 | 1.282143 |
| 6 1.141879 .0521953 2.90 0.004 1.044027 | 1.248902 |
| 7 1.120946 .0545558 2.35 0.019 1.01896 | 1.233139 |
| 8 1.138397 .0565225 2.61 0.009 1.032834 | 1.254748 |
| 9 1.13532 .053764 2.68 0.007 1.034687 | 1.245741 |
| 10 1.108542 .0530374 2.15 0.031 1.009315 | 1.217523 |
| 11 1.111603 .0528354 2.23 0.026 1.012725 | 1.220136 |
| 12 1.113446 .0561459 2.13 0.033 1.008665 | 1.229111 |
| 13 1.086908 .0556485 1.63 0.104 .9831325 | 1.201637 |
| 14 1.151276 .0550798 2.94 0.003 1.048228 | 1.264454 |
| 15 1.101207 .0573155 1.85 0.064 .9944106 | 1.219473 |
| 16 1.198039 .057059 3.79 0.000 1.091266 | 1.315258 |
| 17 1.114881 .051433 2.36 0.018 1.018497 | 1.220386 |
| 18 1.121558 .0534725 2.41 0.016 1.021501 | 1.231415 |
| 19 1.196763 .0568298 3.78 0.000 1.090405 | 1.313495 |
| 20 1.149461 .0539642 2.97 0.003 1.048413 | 1.260248 |
| 21 1.129855 .0516536 2.67 0.008 1.033019 | 1.235768 |
| 22 1.161826 .0552996 3.15 0.002 1.058342 | 1.275428 |
| 23 1.140035 .0603431 2.48 0.013 1.027693 | 1.264658 |
| 24 1.134731 .0607051 2.36 0.018 1.021777 | 1.260172 |
| 25 1.151114 .0534326 3.03 0.002 1.05101 | 1.260751 |
| 26 1.112894 .0558404 2.13 0.033 1.008658 | 1.227901 |
| 27 1.180197 .0583163 3.35 0.001 1.071259 | 1.300212 |
| 28 1.172007 .052807 3.52 0.000 1.072945 | 1.280214 |
| 29 1.178006 .0613888 3.14 0.002 1.063626 | 1.304685 |
| 30 1.234665 .0554909 4.69 0.000 1.130557 | 1.348359 |
| 31 1.239509 .0548507 4.85 0.000 1.136534 | 1.351814 |
| 32 1.197979 .0558972 3.87 0.000 1.093283 | 1.312701 |
| 33 1.180436 .0534012 3.67 0.000 1.080278 | 1.289881 |
| 34 1.217653 .0623156 3.85 0.000 1.101443 | 1.346125 |
| 35 1.208399 .0573715 3.99 0.000 1.101026 | 1.326243 |
| 36 1.159551 .0533911 3.21 0.001 1.059489 | 1.269063 |
| 37 1.091579 .0527885 1.81 0.070 .992867 | 1.200104 |
| 38 1.075604 .0502381 1.56 0.119 .9815112 | 1.178716 |
| 39 1.144882 .0544745 2.84 0.004 1.042942 | 1.256787 |
| year |  |
| 2018 1.020104 .0108425 1.87 0.061 .9990726 | 1.041578 |
| 2019 1.055764 .0139813 4.10 0.000 1.028713 | 1.083525 |
| 2020 1 (omitted) |  |
| _cons 5.16e-06 2.52e-07 -249.54 0.000 4.69e-06 | 5.68e-06 |
| ln(popula~n) 1 (exposure) |  |
|  |  |
| /lnalpha -6.098011 .2812266 -6.649205 | -5.546817 |
|  |  |
| alpha .0022473 .000632 .0012951 | .0038999 |
|  |  |
| Note: Estimates are transformed only in the first equation. |  |
| Note: _cons estimates baseline incidence rate. |  |
| . |  |

**Table S9.- Stroke model for men (full specification)**

| Negative binomial regression Number of obs = | 1,092 |
| --- | --- |
| Wald chi2(49) = | 39081.31 |
| Dispersion = mean Prob > chi2 = | 0.0000 |
| Log pseudolikelihood = -3344.234 Pseudo R2 = | 0.3912 |
|  |  |
| Robust |  |
| _freq IRR Std. Err. z P>z [95% Conf. | Interval] |
|  |  |
| t2 1.000538 .0001684 3.19 0.001 1.000208 | 1.000868 |
| 1.level .6974011 .0257193 -9.77 0.000 .6487709 | .7496765 |
| slope 1.008319 .0017102 4.88 0.000 1.004972 | 1.011676 |
| age_group |  |
| 2 2.085569 .0998443 15.35 0.000 1.898778 | 2.290735 |
| 3 4.851732 .2149651 35.65 0.000 4.448184 | 5.291891 |
| 4 11.51072 .4788644 58.73 0.000 10.60941 | 12.4886 |
| 5 24.86898 1.026878 77.83 0.000 22.93562 | 26.96531 |
| 6 44.50393 1.839101 91.85 0.000 41.04147 | 48.2585 |
| 7 66.71144 2.787259 100.53 0.000 61.46621 | 72.40428 |
| week |  |
| 2 1.185353 .0598442 3.37 0.001 1.073677 | 1.308645 |
| 3 1.133294 .0605332 2.34 0.019 1.02065 | 1.258369 |
| 4 1.153796 .0610486 2.70 0.007 1.040138 | 1.279873 |
| 5 1.128774 .0611074 2.24 0.025 1.015141 | 1.255127 |
| 6 1.122609 .0560226 2.32 0.020 1.018006 | 1.23796 |
| 7 1.074541 .0542789 1.42 0.155 .9732531 | 1.18637 |
| 8 1.118073 .0635557 1.96 0.050 1.000194 | 1.249844 |
| 9 1.086047 .0570577 1.57 0.116 .9797806 | 1.203838 |
| 10 1.049786 .0527843 0.97 0.334 .9512646 | 1.15851 |
| 11 1.087949 .058879 1.56 0.119 .9784582 | 1.209692 |
| 12 1.10719 .0655141 1.72 0.085 .985951 | 1.243338 |
| 13 1.109453 .0590284 1.95 0.051 .9995875 | 1.231394 |
| 14 1.128075 .0556902 2.44 0.015 1.024038 | 1.242681 |
| 15 1.087641 .0586101 1.56 0.119 .9786261 | 1.208801 |
| 16 1.202824 .063256 3.51 0.000 1.08502 | 1.333419 |
| 17 1.108936 .053168 2.16 0.031 1.009475 | 1.218197 |
| 18 1.061698 .0546416 1.16 0.245 .9598263 | 1.174381 |
| 19 1.142696 .0558362 2.73 0.006 1.038336 | 1.257544 |
| 20 1.157069 .0589325 2.86 0.004 1.047141 | 1.278536 |
| 21 1.092961 .051238 1.90 0.058 .9970115 | 1.198143 |
| 22 1.109993 .0541323 2.14 0.032 1.008808 | 1.221326 |
| 23 1.129666 .0618309 2.23 0.026 1.014753 | 1.257591 |
| 24 1.145172 .0617521 2.51 0.012 1.030316 | 1.272831 |
| 25 1.147222 .0558513 2.82 0.005 1.042816 | 1.262081 |
| 26 1.110826 .062218 1.88 0.061 .9953366 | 1.239717 |
| 27 1.184279 .0614254 3.26 0.001 1.069804 | 1.311003 |
| 28 1.161694 .05674 3.07 0.002 1.055643 | 1.278399 |
| 29 1.188646 .0636939 3.23 0.001 1.070141 | 1.320275 |
| 30 1.256792 .0609535 4.71 0.000 1.142828 | 1.382121 |
| 31 1.212653 .0544917 4.29 0.000 1.110419 | 1.324299 |
| 32 1.192612 .0576063 3.65 0.000 1.084885 | 1.311035 |
| 33 1.147821 .0533252 2.97 0.003 1.047922 | 1.257242 |
| 34 1.205571 .0649499 3.47 0.001 1.084762 | 1.339834 |
| 35 1.16599 .0600177 2.98 0.003 1.054097 | 1.289761 |
| 36 1.150285 .0565674 2.85 0.004 1.044591 | 1.266674 |
| 37 1.097399 .058687 1.74 0.082 .9881974 | 1.218667 |
| 38 1.078165 .0508724 1.60 0.111 .982929 | 1.182629 |
| 39 1.101412 .0606629 1.75 0.079 .9887071 | 1.226964 |
| year |  |
| 2018 1.017203 .0145493 1.19 0.233 .9890831 | 1.046123 |
| 2019 1.037363 .0186509 2.04 0.041 1.001444 | 1.07457 |
| 2020 1 (omitted) |  |
| _cons 4.37e-06 2.50e-07 -215.58 0.000 3.91e-06 | 4.89e-06 |
| ln(popula~n) 1 (exposure) |  |
|  |  |
| /lnalpha -6.483374 .6108948 -7.680706 | -5.286042 |
|  |  |
| alpha .0015286 .0009338 .0004616 | .0050618 |
|  |  |
| Note: Estimates are transformed only in the first equation. |  |
| Note: _cons estimates baseline incidence rate. |  |

**Table S10.- Stroke model for women (full specification)**

| Negative binomial regression Number of obs = | 1,092 |
| --- | --- |
| Wald chi2(49) = | 41822.17 |
| Dispersion = mean Prob > chi2 = | 0.0000 |
| Log pseudolikelihood = -3408.0837 Pseudo R2 = | 0.3785 |
|  |  |
| Robust |  |
| _freq IRR Std. Err. z P>z [95% Conf. | Interval] |
|  |  |
| t2 1.000777 .000145 5.36 0.000 1.000493 | 1.001061 |
| 1.level .6131033 .0223332 -13.43 0.000 .5708571 | .6584759 |
| slope 1.008525 .0018111 4.73 0.000 1.004981 | 1.012081 |
| age_group |  |
| 2 1.848986 .0735629 15.45 0.000 1.710284 | 1.998937 |
| 3 3.873116 .1384945 37.87 0.000 3.610965 | 4.154298 |
| 4 6.48981 .2211874 54.87 0.000 6.070453 | 6.938136 |
| 5 11.17487 .3780904 71.34 0.000 10.45786 | 11.94103 |
| 6 21.16703 .7062552 91.48 0.000 19.82708 | 22.59752 |
| 7 38.59122 1.290046 109.28 0.000 36.14383 | 41.20433 |
| week |  |
| 2 1.190679 .0793314 2.62 0.009 1.044917 | 1.356775 |
| 3 1.210248 .0730705 3.16 0.002 1.075181 | 1.362281 |
| 4 1.172742 .0699129 2.67 0.008 1.043418 | 1.318095 |
| 5 1.203854 .0721155 3.10 0.002 1.070492 | 1.35383 |
| 6 1.162434 .0709856 2.46 0.014 1.031308 | 1.310232 |
| 7 1.166216 .0731603 2.45 0.014 1.031289 | 1.318795 |
| 8 1.155339 .0711902 2.34 0.019 1.023905 | 1.303644 |
| 9 1.191286 .0732735 2.85 0.004 1.055992 | 1.343915 |
| 10 1.170172 .0753734 2.44 0.015 1.031387 | 1.327631 |
| 11 1.13636 .0675744 2.15 0.032 1.011343 | 1.27683 |
| 12 1.123508 .0715309 1.83 0.067 .9917046 | 1.272829 |
| 13 1.063547 .0727706 0.90 0.368 .9300689 | 1.21618 |
| 14 1.177246 .0757287 2.54 0.011 1.037796 | 1.335435 |
| 15 1.123098 .0764517 1.71 0.088 .9828216 | 1.283397 |
| 16 1.186729 .0760073 2.67 0.008 1.046728 | 1.345455 |
| 17 1.125738 .0676966 1.97 0.049 1.000576 | 1.266557 |
| 18 1.184247 .0752919 2.66 0.008 1.045501 | 1.341404 |
| 19 1.258175 .0793991 3.64 0.000 1.111795 | 1.423828 |
| 20 1.149966 .0712607 2.25 0.024 1.018446 | 1.29847 |
| 21 1.173762 .0712768 2.64 0.008 1.042055 | 1.322115 |
| 22 1.220005 .0761592 3.19 0.001 1.079506 | 1.37879 |
| 23 1.157824 .0747785 2.27 0.023 1.020158 | 1.314068 |
| 24 1.13416 .0773045 1.85 0.065 .9923308 | 1.296261 |
| 25 1.161141 .071848 2.41 0.016 1.028526 | 1.310856 |
| 26 1.125273 .0713084 1.86 0.063 .9938426 | 1.274085 |
| 27 1.185187 .0750545 2.68 0.007 1.046846 | 1.34181 |
| 28 1.189502 .0687153 3.00 0.003 1.062167 | 1.332102 |
| 29 1.179156 .0808225 2.40 0.016 1.030926 | 1.348698 |
| 30 1.218693 .0747925 3.22 0.001 1.080575 | 1.374464 |
| 31 1.272104 .0763647 4.01 0.000 1.130901 | 1.430936 |
| 32 1.204834 .0732472 3.07 0.002 1.069495 | 1.357299 |
| 33 1.216393 .0722807 3.30 0.001 1.082664 | 1.36664 |
| 34 1.235483 .0809989 3.23 0.001 1.086504 | 1.404888 |
| 35 1.259795 .0748203 3.89 0.000 1.121363 | 1.415317 |
| 36 1.176906 .0698873 2.74 0.006 1.0476 | 1.322172 |
| 37 1.098753 .0702756 1.47 0.141 .9692993 | 1.245497 |
| 38 1.084522 .0678215 1.30 0.194 .9594182 | 1.22594 |
| 39 1.197052 .0732142 2.94 0.003 1.061822 | 1.349504 |
| year |  |
| 2018 1.026393 .0141338 1.89 0.059 .999062 | 1.054472 |
| 2019 1.072346 .0172844 4.33 0.000 1.038999 | 1.106764 |
| 2020 1 (omitted) |  |
| _cons 5.80e-06 3.67e-07 -190.52 0.000 5.12e-06 | 6.57e-06 |
| ln(popula~n) 1 (exposure) |  |
|  |  |
| /lnalpha -9.106274 9.093659 -26.92952 | 8.71697 |
|  |  |
| alpha .000111 .0010091 2.02e-12 | 6105.654 |
|  |  |
| Note: Estimates are transformed only in the first equation. |  |
| Note: _cons estimates baseline incidence rate. |  |
|  |  |

**Table S11.- Myocardial infarction model for both sexes (full specification)**

| Negative binomial regression Number of obs = | 1,092 |
| --- | --- |
| Wald chi2(49) = | 59360.07 |
| Dispersion = mean Prob > chi2 = | 0.0000 |
| Log pseudolikelihood = -4764.4748 Pseudo R2 = | 0.3193 |
|  |  |
| Robust |  |
| _freq IRR Std. Err. z P>z [95% Conf. | Interval] |
|  |  |
| t2 1.000258 .0000904 2.86 0.004 1.000081 | 1.000435 |
| 1.level .5632285 .0127753 -25.31 0.000 .5387379 | .5888326 |
| slope 1.009219 .0010515 8.81 0.000 1.00716 | 1.011282 |
| age_group |  |
| 2 1.369997 .0171599 25.13 0.000 1.336774 | 1.404046 |
| 3 1.942962 .0224161 57.57 0.000 1.899521 | 1.987397 |
| 4 2.682306 .0301168 87.88 0.000 2.623923 | 2.741988 |
| 5 3.842396 .0431733 119.80 0.000 3.758703 | 3.927953 |
| 6 5.462004 .0635654 145.89 0.000 5.338828 | 5.588021 |
| 7 6.938959 .0868657 154.74 0.000 6.770777 | 7.111319 |
| week |  |
| 2 1.084378 .0383408 2.29 0.022 1.011776 | 1.16219 |
| 3 1.094701 .0375072 2.64 0.008 1.023602 | 1.170738 |
| 4 1.048641 .0366252 1.36 0.174 .9792585 | 1.122939 |
| 5 1.02366 .0350322 0.68 0.494 .9572505 | 1.094677 |
| 6 1.007432 .0353713 0.21 0.833 .9404374 | 1.0792 |
| 7 1.033626 .0357818 0.96 0.339 .9658207 | 1.10619 |
| 8 1.039005 .035673 1.11 0.265 .9713879 | 1.111329 |
| 9 1.022553 .0351008 0.65 0.516 .9560198 | 1.093716 |
| 10 1.03603 .0356161 1.03 0.303 .968524 | 1.108242 |
| 11 1.009979 .0353222 0.28 0.776 .9430686 | 1.081638 |
| 12 1.013477 .0343301 0.40 0.693 .9483759 | 1.083047 |
| 13 1.012377 .0363124 0.34 0.732 .9436497 | 1.086109 |
| 14 1.066664 .0367205 1.87 0.061 .9970673 | 1.141118 |
| 15 1.088327 .0369437 2.49 0.013 1.018275 | 1.163199 |
| 16 1.095117 .0384543 2.59 0.010 1.022283 | 1.17314 |
| 17 1.063722 .0376394 1.75 0.081 .9924504 | 1.140112 |
| 18 1.0975 .0377737 2.70 0.007 1.025907 | 1.17409 |
| 19 1.097248 .0384027 2.65 0.008 1.024503 | 1.175157 |
| 20 1.060689 .0363976 1.72 0.086 .9916973 | 1.134481 |
| 21 1.037495 .0354366 1.08 0.281 .970314 | 1.109327 |
| 22 1.027886 .0375223 0.75 0.451 .9569131 | 1.104124 |
| 23 1.002739 .0349098 0.08 0.937 .9365989 | 1.073549 |
| 24 1.017982 .0367475 0.49 0.622 .9484471 | 1.092615 |
| 25 1.099029 .0400142 2.59 0.009 1.023336 | 1.180322 |
| 26 1.036942 .0377286 1.00 0.319 .9655707 | 1.113589 |
| 27 1.070752 .0367102 1.99 0.046 1.001166 | 1.145175 |
| 28 1.082182 .0369957 2.31 0.021 1.012048 | 1.157177 |
| 29 1.079437 .0389009 2.12 0.034 1.005823 | 1.158439 |
| 30 1.074902 .0385229 2.02 0.044 1.001989 | 1.15312 |
| 31 1.141638 .0402518 3.76 0.000 1.06541 | 1.22332 |
| 32 1.136967 .0387429 3.77 0.000 1.063512 | 1.215495 |
| 33 1.151155 .0382728 4.23 0.000 1.078534 | 1.228667 |
| 34 1.150815 .0385379 4.19 0.000 1.077708 | 1.228882 |
| 35 1.13229 .0396313 3.55 0.000 1.057218 | 1.212692 |
| 36 1.114604 .0383631 3.15 0.002 1.041893 | 1.192388 |
| 37 1.040852 .0381016 1.09 0.274 .9687898 | 1.118273 |
| 38 1.042476 .037663 1.15 0.250 .9712112 | 1.118971 |
| 39 1.074437 .0420627 1.83 0.067 .9950788 | 1.160124 |
| year |  |
| 2018 .9957206 .0074248 -0.58 0.565 .9812742 | 1.01038 |
| 2019 1.026992 .0103495 2.64 0.008 1.006907 | 1.047479 |
| 2020 1 (omitted) |  |
| _cons .0000659 2.12e-06 -299.05 0.000 .0000619 | .0000702 |
| ln(popula~n) 1 (exposure) |  |
|  |  |
| /lnalpha -5.874157 .1393333 -6.147246 | -5.601069 |
|  |  |
| alpha .0028112 .0003917 .0021394 | .0036939 |
|  |  |
| Note: Estimates are transformed only in the first equation. |  |
| Note: _cons estimates baseline incidence rate. |  |
| . |  |

**Table S12.- Myocardial infarction model for men (full specification)**

| Negative binomial regression Number of obs = | 1,092 |
| --- | --- |
| Wald chi2(49) = | 34004.83 |
| Dispersion = mean Prob > chi2 = | 0.0000 |
| Log pseudolikelihood = -4239.1959 Pseudo R2 = | 0.3185 |
|  |  |
| Robust |  |
| _freq IRR Std. Err. z P>z [95% Conf. | Interval] |
|  |  |
| t2 1.000412 .0001081 3.81 0.000 1.0002 | 1.000624 |
| 1.level .5954871 .0154348 -20.00 0.000 .565991 | .6265203 |
| slope 1.00748 .0012411 6.05 0.000 1.00505 | 1.009915 |
| age_group |  |
| 2 1.336535 .0201925 19.20 0.000 1.297538 | 1.376703 |
| 3 1.774433 .0246041 41.36 0.000 1.726859 | 1.823317 |
| 4 2.47976 .0326684 68.94 0.000 2.416551 | 2.544623 |
| 5 3.680071 .0486927 98.47 0.000 3.585862 | 3.776755 |
| 6 5.04252 .0698954 116.72 0.000 4.907372 | 5.181391 |
| 7 6.486943 .0986719 122.92 0.000 6.296404 | 6.683248 |
| week |  |
| 2 .9960757 .0388791 -0.10 0.920 .9227159 | 1.075268 |
| 3 1.004797 .0397922 0.12 0.904 .9297557 | 1.085895 |
| 4 .9633895 .0391432 -0.92 0.359 .8896455 | 1.043246 |
| 5 .919507 .037969 -2.03 0.042 .8480209 | .9970192 |
| 6 .9409488 .0367337 -1.56 0.119 .8716375 | 1.015772 |
| 7 .9549389 .038884 -1.13 0.257 .8816895 | 1.034274 |
| 8 .9730944 .0379268 -0.70 0.484 .9015275 | 1.050343 |
| 9 .9493994 .0368372 -1.34 0.181 .8798768 | 1.024415 |
| 10 .9456707 .0375312 -1.41 0.159 .874899 | 1.022167 |
| 11 .9293614 .036546 -1.86 0.062 .8604233 | 1.003823 |
| 12 .9522591 .0368792 -1.26 0.207 .8826524 | 1.027355 |
| 13 .9038498 .0361817 -2.53 0.012 .8356457 | .9776207 |
| 14 .991871 .0388837 -0.21 0.835 .9185146 | 1.071086 |
| 15 .9938781 .0383273 -0.16 0.873 .9215266 | 1.07191 |
| 16 .9961919 .0395977 -0.10 0.924 .9215279 | 1.076905 |
| 17 .9495713 .0391596 -1.25 0.210 .8758398 | 1.02951 |
| 18 1.015478 .0399504 0.39 0.696 .9401189 | 1.096877 |
| 19 .9956952 .0395894 -0.11 0.914 .9210478 | 1.076393 |
| 20 .9496077 .0369898 -1.33 0.184 .8798074 | 1.024946 |
| 21 .9577398 .0366208 -1.13 0.259 .8885879 | 1.032273 |
| 22 .9566502 .0401615 -1.06 0.291 .8810865 | 1.038694 |
| 23 .9093892 .0362554 -2.38 0.017 .8410353 | .9832986 |
| 24 .9263059 .0375766 -1.89 0.059 .8555088 | 1.002962 |
| 25 .9919383 .0420178 -0.19 0.848 .9129109 | 1.077807 |
| 26 .9544355 .039142 -1.14 0.255 .8807209 | 1.03432 |
| 27 1.001292 .038492 0.03 0.973 .9286211 | 1.07965 |
| 28 1.043515 .0402677 1.10 0.270 .9675021 | 1.125499 |
| 29 1.022144 .0406853 0.55 0.582 .945433 | 1.105078 |
| 30 1.03468 .0422846 0.83 0.404 .9550362 | 1.120966 |
| 31 1.060911 .043996 1.43 0.154 .9780922 | 1.150743 |
| 32 1.062873 .0419362 1.55 0.122 .9837771 | 1.148328 |
| 33 1.087862 .0407682 2.25 0.025 1.010822 | 1.170774 |
| 34 1.072644 .0404761 1.86 0.063 .996175 | 1.154983 |
| 35 1.044857 .043701 1.05 0.294 .9626208 | 1.134118 |
| 36 1.026758 .0409524 0.66 0.508 .9495503 | 1.110244 |
| 37 .9976333 .0412584 -0.06 0.954 .9199588 | 1.081866 |
| 38 1.032793 .0424546 0.78 0.432 .9528476 | 1.119447 |
| 39 1.003066 .0439413 0.07 0.944 .9205366 | 1.092995 |
| year |  |
| 2018 .9936994 .009154 -0.69 0.493 .975919 | 1.011804 |
| 2019 1.00857 .0121971 0.71 0.480 .9849453 | 1.032762 |
| 2020 1 (omitted) |  |
| _cons .0000843 3.06e-06 -258.17 0.000 .0000785 | .0000905 |
| ln(popula~n) 1 (exposure) |  |
|  |  |
| /lnalpha -6.051744 .2271574 -6.496964 | -5.606524 |
|  |  |
| alpha .0023538 .0005347 .001508 | .0036738 |
|  |  |
| Note: Estimates are transformed only in the first equation. |  |
| Note: _cons estimates baseline incidence rate. |  |

**Table S13.- Myocardial infarction model for women (full specification)**

| Negative binomial regression Number of obs = | 1,092 |
| --- | --- |
| Wald chi2(49) = | 38893.45 |
| Dispersion = mean Prob > chi2 = | 0.0000 |
| Log pseudolikelihood = -4260.1373 Pseudo R2 = | 0.3220 |
|  |  |
| Robust |  |
| _freq IRR Std. Err. z P>z [95% Conf. | Interval] |
|  |  |
| t2 1.000078 .0001072 0.73 0.466 .9998681 | 1.000288 |
| 1.level .5322442 .0156294 -21.48 0.000 .502476 | .563776 |
| slope 1.011086 .001411 7.90 0.000 1.008324 | 1.013855 |
| age_group |  |
| 2 1.432709 .0232687 22.14 0.000 1.387821 | 1.479048 |
| 3 2.16852 .0326764 51.37 0.000 2.105412 | 2.23352 |
| 4 2.945557 .0428178 74.32 0.000 2.86282 | 3.030686 |
| 5 4.077511 .0601468 95.28 0.000 3.961313 | 4.197117 |
| 6 6.094771 .0903377 121.94 0.000 5.92026 | 6.274427 |
| 7 7.867528 .1267253 128.06 0.000 7.623031 | 8.119867 |
| week |  |
| 2 1.198119 .0502363 4.31 0.000 1.103595 | 1.300739 |
| 3 1.206092 .0447259 5.05 0.000 1.121541 | 1.297018 |
| 4 1.15683 .04483 3.76 0.000 1.072219 | 1.248118 |
| 5 1.156546 .0423151 3.98 0.000 1.076514 | 1.242529 |
| 6 1.092405 .0420475 2.30 0.022 1.013025 | 1.178005 |
| 7 1.134377 .0424785 3.37 0.001 1.054103 | 1.220765 |
| 8 1.12289 .0430649 3.02 0.003 1.041579 | 1.210549 |
| 9 1.114672 .0428927 2.82 0.005 1.033696 | 1.201991 |
| 10 1.148879 .0439912 3.62 0.000 1.065814 | 1.238418 |
| 11 1.112021 .0436523 2.70 0.007 1.029673 | 1.200956 |
| 12 1.091418 .0426096 2.24 0.025 1.01102 | 1.178209 |
| 13 1.149116 .0447961 3.57 0.000 1.064588 | 1.240356 |
| 14 1.159916 .0449484 3.83 0.000 1.075081 | 1.251445 |
| 15 1.20871 .0463575 4.94 0.000 1.121181 | 1.303071 |
| 16 1.214963 .0461548 5.13 0.000 1.127787 | 1.308878 |
| 17 1.207724 .0483897 4.71 0.000 1.11651 | 1.30639 |
| 18 1.202121 .0450071 4.92 0.000 1.117067 | 1.29365 |
| 19 1.228303 .0468425 5.39 0.000 1.13984 | 1.323631 |
| 20 1.205758 .047514 4.75 0.000 1.116138 | 1.302575 |
| 21 1.144847 .0433703 3.57 0.000 1.062922 | 1.233087 |
| 22 1.128929 .0443699 3.09 0.002 1.045231 | 1.21933 |
| 23 1.124719 .0432509 3.06 0.002 1.043064 | 1.212765 |
| 24 1.138142 .0454534 3.24 0.001 1.052453 | 1.230809 |
| 25 1.240167 .0473657 5.64 0.000 1.150721 | 1.336565 |
| 26 1.148022 .0480592 3.30 0.001 1.057588 | 1.246188 |
| 27 1.165681 .0450929 3.96 0.000 1.080568 | 1.257498 |
| 28 1.140092 .0437624 3.42 0.001 1.057466 | 1.229174 |
| 29 1.158997 .0444685 3.85 0.000 1.075037 | 1.249514 |
| 30 1.135245 .0455113 3.16 0.002 1.049459 | 1.228044 |
| 31 1.253983 .0462381 6.14 0.000 1.166555 | 1.347963 |
| 32 1.234607 .0462976 5.62 0.000 1.14712 | 1.328767 |
| 33 1.24005 .0449489 5.94 0.000 1.155008 | 1.331353 |
| 34 1.257129 .0459733 6.26 0.000 1.170177 | 1.350543 |
| 35 1.249806 .0463709 6.01 0.000 1.162146 | 1.344077 |
| 36 1.232659 .0482051 5.35 0.000 1.141709 | 1.330855 |
| 37 1.097347 .0444401 2.29 0.022 1.013613 | 1.187998 |
| 38 1.059704 .0443737 1.38 0.166 .9762064 | 1.150343 |
| 39 1.172147 .0500445 3.72 0.000 1.078053 | 1.274453 |
| year |  |
| 2018 .9982611 .0092724 -0.19 0.851 .980252 | 1.016601 |
| 2019 1.04694 .0125297 3.83 0.000 1.022668 | 1.071788 |
| 2020 1 (omitted) |  |
| _cons .0000502 1.75e-06 -284.26 0.000 .0000469 | .0000537 |
| ln(popula~n) 1 (exposure) |  |
|  |  |
| /lnalpha -5.899332 .1940698 -6.279702 | -5.518962 |
|  |  |
| alpha .0027413 .000532 .001874 | .00401 |
|  |  |
| Note: Estimates are transformed only in the first equation. |  |
| Note: _cons estimates baseline incidence rate. |  |
| . |  |

**Table S14.- All cancer model for both sexes (full specification)**

| Negative binomial regression Number of obs = | 1,092 |
| --- | --- |
| Wald chi2(49) = | 10618.06 |
| Dispersion = mean Prob > chi2 = | 0.0000 |
| Log pseudolikelihood = -4684.8141 Pseudo R2 = | 0.2166 |
|  |  |
| Robust |  |
| _freq IRR Std. Err. z P>z [95% Conf. | Interval] |
|  |  |
| t2 1.001763 .0001339 13.18 0.000 1.001501 | 1.002026 |
| 1.level .3229056 .017297 -21.10 0.000 .290723 | .3586507 |
| slope 1.021924 .0029782 7.44 0.000 1.016104 | 1.027778 |
| age_group |  |
| 2 1.505 .0260246 23.64 0.000 1.454848 | 1.556882 |
| 3 2.116947 .0339612 46.75 0.000 2.051419 | 2.184567 |
| 4 2.274961 .037202 50.26 0.000 2.203203 | 2.349057 |
| 5 2.789626 .0463298 61.77 0.000 2.700283 | 2.881925 |
| 6 3.383317 .0558962 73.78 0.000 3.275518 | 3.494665 |
| 7 2.962336 .0532823 60.38 0.000 2.859724 | 3.06863 |
| week |  |
| 2 1.675381 .1095076 7.90 0.000 1.473929 | 1.904366 |
| 3 1.578713 .1048415 6.88 0.000 1.386038 | 1.798171 |
| 4 1.587551 .1047894 7.00 0.000 1.394898 | 1.806812 |
| 5 1.617137 .1052897 7.38 0.000 1.423398 | 1.837247 |
| 6 1.48305 .098525 5.93 0.000 1.301988 | 1.689291 |
| 7 1.44982 .0970272 5.55 0.000 1.271595 | 1.653026 |
| 8 1.416125 .0953333 5.17 0.000 1.241077 | 1.615863 |
| 9 1.446203 .0941701 5.67 0.000 1.272925 | 1.643068 |
| 10 1.644745 .1064435 7.69 0.000 1.448809 | 1.867179 |
| 11 1.635903 .1046511 7.69 0.000 1.443128 | 1.854428 |
| 12 1.896215 .1660826 7.31 0.000 1.597107 | 2.251341 |
| 13 1.526957 .111676 5.79 0.000 1.32304 | 1.762303 |
| 14 1.602895 .1030573 7.34 0.000 1.413115 | 1.818162 |
| 15 1.53904 .1039582 6.38 0.000 1.348197 | 1.756898 |
| 16 1.516361 .1016027 6.21 0.000 1.329745 | 1.729166 |
| 17 1.5587 .1016861 6.80 0.000 1.371614 | 1.771303 |
| 18 1.263232 .0815037 3.62 0.000 1.113176 | 1.433517 |
| 19 1.605704 .1044403 7.28 0.000 1.413516 | 1.824024 |
| 20 1.590137 .101126 7.29 0.000 1.403788 | 1.801222 |
| 21 1.30187 .0857415 4.01 0.000 1.144214 | 1.481249 |
| 22 1.524271 .0975028 6.59 0.000 1.344663 | 1.727869 |
| 23 1.52467 .1049047 6.13 0.000 1.332321 | 1.744788 |
| 24 1.507955 .0987624 6.27 0.000 1.326293 | 1.714499 |
| 25 1.523311 .1008712 6.36 0.000 1.337899 | 1.734418 |
| 26 1.418005 .0965187 5.13 0.000 1.240907 | 1.620377 |
| 27 1.397544 .0968438 4.83 0.000 1.22006 | 1.600848 |
| 28 1.517299 .1011297 6.26 0.000 1.33149 | 1.729039 |
| 29 1.243726 .0854404 3.17 0.001 1.08705 | 1.422984 |
| 30 1.52373 .0978558 6.56 0.000 1.343516 | 1.728117 |
| 31 1.565577 .1012445 6.93 0.000 1.379203 | 1.777137 |
| 32 1.625801 .106092 7.45 0.000 1.430613 | 1.847621 |
| 33 1.363814 .0890037 4.75 0.000 1.200066 | 1.549906 |
| 34 1.609272 .1026087 7.46 0.000 1.420221 | 1.823488 |
| 35 1.655772 .104944 7.96 0.000 1.462349 | 1.87478 |
| 36 1.678092 .1073801 8.09 0.000 1.480294 | 1.902321 |
| 37 1.651763 .1100573 7.53 0.000 1.449546 | 1.88219 |
| 38 .7209728 .0773946 -3.05 0.002 .5841771 | .8898018 |
| 39 1.558824 .1013984 6.82 0.000 1.372234 | 1.770786 |
| year |  |
| 2018 .9871699 .0114337 -1.11 0.265 .9650128 | 1.009836 |
| 2019 .9844372 .0140865 -1.10 0.273 .9572116 | 1.012437 |
| 2020 1 (omitted) |  |
| _cons .0000301 1.85e-06 -169.69 0.000 .0000267 | .0000339 |
| ln(popula~n) 1 (exposure) |  |
|  |  |
| /lnalpha -4.37883 .1389331 -4.651134 | -4.106526 |
|  |  |
| alpha .01254 .0017422 .0095508 | .0164649 |
|  |  |
| Note: Estimates are transformed only in the first equation. |  |
| Note: _cons estimates baseline incidence rate. |  |
| . |  |

**Table S15.- All cancer model for men (full specification)**

| Negative binomial regression Number of obs = | 1,092 |
| --- | --- |
| Wald chi2(49) = | 17640.60 |
| Dispersion = mean Prob > chi2 = | 0.0000 |
| Log pseudolikelihood = -3269.5035 Pseudo R2 = | 0.3265 |
|  |  |
| Robust |  |
| _freq IRR Std. Err. z P>z [95% Conf. | Interval] |
|  |  |
| t2 1.001916 .0002158 8.89 0.000 1.001494 | 1.00234 |
| 1.level .3635448 .0266288 -13.81 0.000 .3149266 | .4196687 |
| slope 1.023929 .0038033 6.37 0.000 1.016502 | 1.031411 |
| age_group |  |
| 2 1.259417 .052464 5.54 0.000 1.160675 | 1.366558 |
| 3 2.854732 .1061019 28.22 0.000 2.65417 | 3.07045 |
| 4 4.923886 .1718292 45.68 0.000 4.598366 | 5.272449 |
| 5 9.237523 .3188408 64.41 0.000 8.633276 | 9.884062 |
| 6 15.14248 .5169444 79.60 0.000 14.16244 | 16.19033 |
| 7 16.09219 .5892929 75.87 0.000 14.97768 | 17.28964 |
| week |  |
| 2 1.599984 .1499431 5.02 0.000 1.331511 | 1.922588 |
| 3 1.52277 .1506977 4.25 0.000 1.254287 | 1.848723 |
| 4 1.541131 .1490969 4.47 0.000 1.274941 | 1.862898 |
| 5 1.485619 .1427554 4.12 0.000 1.230592 | 1.793497 |
| 6 1.392857 .134545 3.43 0.001 1.152613 | 1.683176 |
| 7 1.367252 .1348143 3.17 0.002 1.126985 | 1.658743 |
| 8 1.287853 .1253964 2.60 0.009 1.064108 | 1.558642 |
| 9 1.320401 .1298211 2.83 0.005 1.088971 | 1.601016 |
| 10 1.613353 .1500872 5.14 0.000 1.344447 | 1.936043 |
| 11 1.508773 .1443619 4.30 0.000 1.250776 | 1.819987 |
| 12 1.578561 .1689052 4.27 0.000 1.279921 | 1.946882 |
| 13 1.289424 .1319182 2.48 0.013 1.055142 | 1.575724 |
| 14 1.443131 .1425044 3.71 0.000 1.189193 | 1.751294 |
| 15 1.357957 .1317245 3.15 0.002 1.122839 | 1.642306 |
| 16 1.324181 .128951 2.88 0.004 1.094097 | 1.60265 |
| 17 1.447482 .1448372 3.70 0.000 1.189709 | 1.761107 |
| 18 1.164936 .1132447 1.57 0.116 .9628438 | 1.409446 |
| 19 1.420659 .1366244 3.65 0.000 1.176603 | 1.715337 |
| 20 1.36555 .1292181 3.29 0.001 1.134386 | 1.643821 |
| 21 1.225958 .1191119 2.10 0.036 1.013385 | 1.483122 |
| 22 1.443446 .1401481 3.78 0.000 1.193315 | 1.746007 |
| 23 1.350342 .1290595 3.14 0.002 1.119669 | 1.628537 |
| 24 1.381137 .1359996 3.28 0.001 1.138727 | 1.675152 |
| 25 1.476277 .146327 3.93 0.000 1.21562 | 1.792826 |
| 26 1.376629 .1337593 3.29 0.001 1.137917 | 1.665419 |
| 27 1.324669 .1350331 2.76 0.006 1.084771 | 1.61762 |
| 28 1.436532 .1381509 3.77 0.000 1.189749 | 1.734503 |
| 29 1.162329 .1135453 1.54 0.124 .9597921 | 1.407606 |
| 30 1.459317 .1371022 4.02 0.000 1.213891 | 1.754364 |
| 31 1.543175 .1459098 4.59 0.000 1.282132 | 1.857367 |
| 32 1.566313 .1515941 4.64 0.000 1.295675 | 1.893483 |
| 33 1.235743 .123787 2.11 0.035 1.015457 | 1.503816 |
| 34 1.479866 .1393865 4.16 0.000 1.230407 | 1.779901 |
| 35 1.546123 .145506 4.63 0.000 1.285693 | 1.859306 |
| 36 1.495238 .13832 4.35 0.000 1.247292 | 1.792472 |
| 37 1.572827 .1450085 4.91 0.000 1.312815 | 1.884336 |
| 38 .6236684 .0837587 -3.52 0.000 .479333 | .8114656 |
| 39 1.519264 .1473883 4.31 0.000 1.25619 | 1.83743 |
| year |  |
| 2018 1.004133 .0191786 0.22 0.829 .9672388 | 1.042435 |
| 2019 1.034134 .0246852 1.41 0.160 .9868663 | 1.083666 |
| 2020 1 (omitted) |  |
| _cons 5.42e-06 4.90e-07 -134.28 0.000 4.54e-06 | 6.47e-06 |
| ln(popula~n) 1 (exposure) |  |
|  |  |
| /lnalpha -4.806917 .320367 -5.434824 | -4.179009 |
|  |  |
| alpha .008173 .0026184 .004362 | .0153137 |
|  |  |
| Note: Estimates are transformed only in the first equation. |  |
| Note: _cons estimates baseline incidence rate. |  |

**Table S16.- All cancer model for women (full specification)**

| Negative binomial regression Number of obs = | 1,092 |
| --- | --- |
| Wald chi2(49) = | 6004.03 |
| Dispersion = mean Prob > chi2 = | 0.0000 |
| Log pseudolikelihood = -4512.3904 Pseudo R2 = | 0.1879 |
|  |  |
| Robust |  |
| _freq IRR Std. Err. z P>z [95% Conf. | Interval] |
|  |  |
| t2 1.001722 .0001358 12.69 0.000 1.001456 | 1.001988 |
| 1.level .3124923 .0180059 -20.19 0.000 .2791211 | .3498532 |
| slope 1.021289 .0032481 6.62 0.000 1.014943 | 1.027675 |
| age_group |  |
| 2 1.478531 .0266816 21.67 0.000 1.42715 | 1.531762 |
| 3 2.017028 .0338337 41.83 0.000 1.951793 | 2.084443 |
| 4 2.0339 .035095 41.14 0.000 1.966266 | 2.103862 |
| 5 2.187676 .0378401 45.26 0.000 2.114753 | 2.263112 |
| 6 2.2858 .0411107 45.97 0.000 2.206628 | 2.367813 |
| 7 1.762547 .036035 27.72 0.000 1.693316 | 1.834609 |
| week |  |
| 2 1.700255 .1122565 8.04 0.000 1.493877 | 1.935143 |
| 3 1.596152 .10625 7.02 0.000 1.400918 | 1.818593 |
| 4 1.609905 .1073346 7.14 0.000 1.412698 | 1.834641 |
| 5 1.657987 .1073683 7.81 0.000 1.460356 | 1.882363 |
| 6 1.504479 .1002915 6.13 0.000 1.320211 | 1.714466 |
| 7 1.476006 .0994852 5.78 0.000 1.293349 | 1.68446 |
| 8 1.44365 .0984387 5.38 0.000 1.263051 | 1.650073 |
| 9 1.490764 .0989721 6.01 0.000 1.308873 | 1.697932 |
| 10 1.655269 .1088466 7.66 0.000 1.455109 | 1.882962 |
| 11 1.673909 .1078773 7.99 0.000 1.475282 | 1.899279 |
| 12 1.96117 .1759047 7.51 0.000 1.645007 | 2.338098 |
| 13 1.596595 .1203074 6.21 0.000 1.377383 | 1.850696 |
| 14 1.659931 .1076338 7.82 0.000 1.461827 | 1.884881 |
| 15 1.591884 .1084715 6.82 0.000 1.392869 | 1.819334 |
| 16 1.566048 .1066171 6.59 0.000 1.370424 | 1.789597 |
| 17 1.590159 .1047214 7.04 0.000 1.397603 | 1.809244 |
| 18 1.293277 .0844458 3.94 0.000 1.137919 | 1.469845 |
| 19 1.663565 .1099796 7.70 0.000 1.46139 | 1.89371 |
| 20 1.653846 .107153 7.77 0.000 1.456618 | 1.87778 |
| 21 1.321378 .0886908 4.15 0.000 1.158496 | 1.507161 |
| 22 1.552063 .1000857 6.82 0.000 1.367789 | 1.761163 |
| 23 1.580232 .1092831 6.62 0.000 1.379923 | 1.809618 |
| 24 1.543968 .1032998 6.49 0.000 1.354217 | 1.760306 |
| 25 1.539862 .1033021 6.43 0.000 1.350139 | 1.756244 |
| 26 1.431395 .1003365 5.12 0.000 1.24765 | 1.642201 |
| 27 1.432058 .100659 5.11 0.000 1.247757 | 1.643582 |
| 28 1.544813 .1056864 6.36 0.000 1.350959 | 1.766485 |
| 29 1.264583 .0886915 3.35 0.001 1.102169 | 1.450929 |
| 30 1.545306 .1004548 6.70 0.000 1.360445 | 1.755286 |
| 31 1.578911 .1022533 7.05 0.000 1.390696 | 1.792599 |
| 32 1.655678 .1083584 7.70 0.000 1.456357 | 1.88228 |
| 33 1.408113 .0922734 5.22 0.000 1.238393 | 1.601093 |
| 34 1.652933 .1069604 7.77 0.000 1.456044 | 1.876445 |
| 35 1.693045 .1081902 8.24 0.000 1.493738 | 1.918945 |
| 36 1.731912 .1111788 8.56 0.000 1.527157 | 1.96412 |
| 37 1.679944 .11432 7.62 0.000 1.47018 | 1.919636 |
| 38 .7444474 .0821884 -2.67 0.008 .5995975 | .9242899 |
| 39 1.577219 .1039591 6.91 0.000 1.386075 | 1.794722 |
| year |  |
| 2018 .9816611 .0118987 -1.53 0.127 .958615 | 1.005261 |
| 2019 .9759958 .0142822 -1.66 0.097 .9484008 | 1.004394 |
| 2020 1 (omitted) |  |
| _cons .0000514 3.20e-06 -158.88 0.000 .0000455 | .0000581 |
| ln(popula~n) 1 (exposure) |  |
|  |  |
| /lnalpha -4.317819 .1455844 -4.603159 | -4.032479 |
|  |  |
| alpha .0133289 .0019405 .0100201 | .0177303 |
|  |  |
| Note: Estimates are transformed only in the first equation. |  |
| Note: _cons estimates baseline incidence rate. |  |
| . |  |

**Table S17.- All cancer (excluding sex-specific cancer) model for both gender (full specification)**

| Negative binomial regression Number of obs = | 1,092 |
| --- | --- |
| Wald chi2(49) = | 29692.92 |
| Dispersion = mean Prob > chi2 = | 0.0000 |
| Log pseudolikelihood = -3717.7049 Pseudo R2 = | 0.3416 |
|  |  |
| Robust |  |
| _freq IRR Std. Err. z P>z [95% Conf. | Interval] |
|  |  |
| t2 1.002168 .0001666 13.03 0.000 1.001842 | 1.002495 |
| 1.level .2934118 .0192796 -18.66 0.000 .2579565 | .3337403 |
| slope 1.028284 .0035453 8.09 0.000 1.021358 | 1.035256 |
| age_group |  |
| 2 1.641117 .0676708 12.01 0.000 1.513703 | 1.779256 |
| 3 7.681097 .2689293 58.23 0.000 7.171684 | 8.226695 |
| 4 13.96019 .4680519 78.63 0.000 13.07232 | 14.90837 |
| 5 23.7498 .7941014 94.74 0.000 22.24329 | 25.35834 |
| 6 35.65174 1.19305 106.80 0.000 33.38844 | 38.06846 |
| 7 32.38668 1.107901 101.66 0.000 30.28643 | 34.63258 |
| week |  |
| 2 1.696314 .150169 5.97 0.000 1.426107 | 2.017717 |
| 3 1.623055 .1483621 5.30 0.000 1.356831 | 1.941516 |
| 4 1.569263 .1404308 5.04 0.000 1.31681 | 1.870116 |
| 5 1.623746 .1479837 5.32 0.000 1.358132 | 1.941307 |
| 6 1.358795 .1237938 3.37 0.001 1.136593 | 1.624438 |
| 7 1.377122 .1245183 3.54 0.000 1.153473 | 1.644135 |
| 8 1.280088 .1188554 2.66 0.008 1.067102 | 1.535583 |
| 9 1.363659 .1235417 3.42 0.001 1.141801 | 1.628625 |
| 10 1.640991 .1458262 5.57 0.000 1.378683 | 1.953205 |
| 11 1.598318 .1426891 5.25 0.000 1.341753 | 1.903943 |
| 12 1.719981 .1824981 5.11 0.000 1.397035 | 2.117582 |
| 13 1.395142 .142236 3.27 0.001 1.142451 | 1.703723 |
| 14 1.480977 .1327567 4.38 0.000 1.242355 | 1.765433 |
| 15 1.466026 .1366771 4.10 0.000 1.221193 | 1.759944 |
| 16 1.429697 .1313446 3.89 0.000 1.194112 | 1.71176 |
| 17 1.544393 .1391722 4.82 0.000 1.294351 | 1.842737 |
| 18 1.219555 .1103825 2.19 0.028 1.021313 | 1.456278 |
| 19 1.566687 .1424275 4.94 0.000 1.310991 | 1.872255 |
| 20 1.511505 .1317836 4.74 0.000 1.274077 | 1.793177 |
| 21 1.271003 .1132641 2.69 0.007 1.067315 | 1.513563 |
| 22 1.482858 .1326766 4.40 0.000 1.244341 | 1.767093 |
| 23 1.418059 .1286788 3.85 0.000 1.187008 | 1.694083 |
| 24 1.395417 .1268419 3.67 0.000 1.167698 | 1.667544 |
| 25 1.451042 .1309282 4.13 0.000 1.215838 | 1.731747 |
| 26 1.398596 .1281738 3.66 0.000 1.168649 | 1.673788 |
| 27 1.392501 .1290685 3.57 0.000 1.161179 | 1.669905 |
| 28 1.514275 .1378427 4.56 0.000 1.266838 | 1.810043 |
| 29 1.205224 .1119691 2.01 0.045 1.004589 | 1.44593 |
| 30 1.533174 .1367278 4.79 0.000 1.287305 | 1.826002 |
| 31 1.574913 .1419468 5.04 0.000 1.31989 | 1.87921 |
| 32 1.634349 .1466598 5.47 0.000 1.37076 | 1.948624 |
| 33 1.330702 .121018 3.14 0.002 1.113448 | 1.590346 |
| 34 1.54105 .1368808 4.87 0.000 1.294823 | 1.8341 |
| 35 1.625813 .1441515 5.48 0.000 1.366468 | 1.93438 |
| 36 1.57313 .1391033 5.12 0.000 1.32281 | 1.870819 |
| 37 1.587749 .1402739 5.23 0.000 1.335304 | 1.887919 |
| 38 .6170984 .0808375 -3.69 0.000 .4773647 | .7977346 |
| 39 1.482701 .134845 4.33 0.000 1.240625 | 1.772012 |
| year |  |
| 2018 .9984459 .0156607 -0.10 0.921 .9682185 | 1.029617 |
| 2019 1.045098 .0190639 2.42 0.016 1.008393 | 1.083138 |
| 2020 1 (omitted) |  |
| _cons 2.06e-06 1.81e-07 -149.43 0.000 1.74e-06 | 2.45e-06 |
| ln(popula~n) 1 (exposure) |  |
|  |  |
| /lnalpha -4.542232 .19857 -4.931422 | -4.153042 |
|  |  |
| alpha .0106496 .0021147 .0072162 | .0157165 |
|  |  |
| Note: Estimates are transformed only in the first equation. |  |
| Note: _cons estimates baseline incidence rate. |  |
| . |  |

**Table S17.- All cancer (excluding sex-specific cancer) model for men (full specification)**

| Negative binomial regression Number of obs = | 1,092 |
| --- | --- |
| Wald chi2(49) = | 17389.11 |
| Dispersion = mean Prob > chi2 = | 0.0000 |
| Log pseudolikelihood = -3102.4967 Pseudo R2 = | 0.3496 |
|  |  |
| Robust |  |
| _freq IRR Std. Err. z P>z [95% Conf. | Interval] |
|  |  |
| t2 1.002021 .0002192 9.23 0.000 1.001591 | 1.00245 |
| 1.level .3510518 .0270366 -13.59 0.000 .3018665 | .4082511 |
| slope 1.025367 .0040633 6.32 0.000 1.017434 | 1.033362 |
| age_group |  |
| 2 1.560947 .0917523 7.58 0.000 1.391088 | 1.751546 |
| 3 5.673508 .2750301 35.81 0.000 5.159275 | 6.238996 |
| 4 10.71587 .4895206 51.92 0.000 9.798125 | 11.71958 |
| 5 20.66351 .9377688 66.73 0.000 18.90489 | 22.58572 |
| 6 34.4448 1.55433 78.43 0.000 31.5292 | 37.63001 |
| 7 36.77677 1.739132 76.23 0.000 33.52132 | 40.34836 |
| week |  |
| 2 1.625853 .1663914 4.75 0.000 1.330358 | 1.986982 |
| 3 1.54482 .1665112 4.03 0.000 1.250632 | 1.90821 |
| 4 1.53892 .1593295 4.16 0.000 1.256285 | 1.88514 |
| 5 1.54881 .1589245 4.26 0.000 1.266647 | 1.893828 |
| 6 1.396852 .1461027 3.20 0.001 1.137941 | 1.714673 |
| 7 1.394169 .1488438 3.11 0.002 1.13094 | 1.718664 |
| 8 1.319822 .1384758 2.64 0.008 1.074502 | 1.621151 |
| 9 1.360625 .1434401 2.92 0.003 1.106631 | 1.672916 |
| 10 1.704488 .1687661 5.39 0.000 1.403828 | 2.069539 |
| 11 1.578741 .1639764 4.40 0.000 1.287954 | 1.935179 |
| 12 1.610536 .1868439 4.11 0.000 1.282979 | 2.021721 |
| 13 1.325816 .1437387 2.60 0.009 1.072013 | 1.639708 |
| 14 1.447567 .1525049 3.51 0.000 1.177504 | 1.779569 |
| 15 1.405529 .1483457 3.23 0.001 1.142879 | 1.72854 |
| 16 1.358481 .1405636 2.96 0.003 1.10912 | 1.663904 |
| 17 1.489632 .1595182 3.72 0.000 1.207612 | 1.837513 |
| 18 1.197367 .1258739 1.71 0.087 .9744153 | 1.471331 |
| 19 1.452344 .1508897 3.59 0.000 1.184772 | 1.780345 |
| 20 1.38292 .1403948 3.19 0.001 1.133398 | 1.687374 |
| 21 1.235194 .1271857 2.05 0.040 1.009459 | 1.511409 |
| 22 1.467677 .1549855 3.63 0.000 1.193286 | 1.805164 |
| 23 1.356423 .1401092 2.95 0.003 1.107827 | 1.660804 |
| 24 1.402724 .150546 3.15 0.002 1.136627 | 1.731118 |
| 25 1.461928 .1514478 3.67 0.000 1.19329 | 1.791042 |
| 26 1.395327 .1502693 3.09 0.002 1.129812 | 1.72324 |
| 27 1.376714 .1505934 2.92 0.003 1.111051 | 1.705899 |
| 28 1.456568 .1509572 3.63 0.000 1.188812 | 1.784631 |
| 29 1.1786 .1235329 1.57 0.117 .9597303 | 1.447384 |
| 30 1.501783 .1525235 4.00 0.000 1.230716 | 1.832553 |
| 31 1.588518 .1636349 4.49 0.000 1.298102 | 1.943906 |
| 32 1.590995 .1654427 4.47 0.000 1.297642 | 1.950665 |
| 33 1.285672 .139855 2.31 0.021 1.038812 | 1.591196 |
| 34 1.52698 .154265 4.19 0.000 1.252679 | 1.861346 |
| 35 1.584429 .161083 4.53 0.000 1.298178 | 1.933799 |
| 36 1.521981 .1530494 4.18 0.000 1.249721 | 1.853555 |
| 37 1.61162 .1604874 4.79 0.000 1.325863 | 1.958964 |
| 38 .5926678 .0859369 -3.61 0.000 .4460536 | .7874729 |
| 39 1.539071 .1608028 4.13 0.000 1.254079 | 1.888829 |
| year |  |
| 2018 1.01979 .0202326 0.99 0.323 .9808961 | 1.060226 |
| 2019 1.041868 .0256146 1.67 0.095 .9928546 | 1.093301 |
| 2020 1 (omitted) |  |
| _cons 2.24e-06 2.29e-07 -127.32 0.000 1.83e-06 | 2.73e-06 |
| ln(popula~n) 1 (exposure) |  |
|  |  |
| /lnalpha -4.951287 .3741794 -5.684665 | -4.217909 |
|  |  |
| alpha .0070743 .0026471 .0033977 | .0147294 |
|  |  |
| Note: Estimates are transformed only in the first equation. |  |
| Note: _cons estimates baseline incidence rate. |  |

**Table S18.- All cancer (excluding sex-specific cancer) model for women (full specification)**

| Negative binomial regression Number of obs = | 1,092 |
| --- | --- |
| Wald chi2(49) = | 19008.64 |
| Dispersion = mean Prob > chi2 = | 0.0000 |
| Log pseudolikelihood = -3288.7147 Pseudo R2 = | 0.3440 |
|  |  |
| Robust |  |
| _freq IRR Std. Err. z P>z [95% Conf. | Interval] |
|  |  |
| t2 1.002263 .0001812 12.50 0.000 1.001908 | 1.002618 |
| 1.level .2540117 .0196723 -17.69 0.000 .2182384 | .2956489 |
| slope 1.030187 .0042183 7.26 0.000 1.021952 | 1.038488 |
| age_group |  |
| 2 1.726899 .08957 10.53 0.000 1.559974 | 1.911687 |
| 3 9.650407 .4230854 51.71 0.000 8.855803 | 10.51631 |
| 4 17.25283 .7290105 67.40 0.000 15.88156 | 18.74249 |
| 5 26.82921 1.131874 77.97 0.000 24.70002 | 29.14194 |
| 6 37.12891 1.578641 85.01 0.000 34.16025 | 40.35557 |
| 7 30.33263 1.319811 78.42 0.000 27.85307 | 33.03291 |
| week |  |
| 2 1.768844 .1692473 5.96 0.000 1.466373 | 2.133706 |
| 3 1.705729 .1660649 5.48 0.000 1.409417 | 2.064337 |
| 4 1.636215 .1599344 5.04 0.000 1.350947 | 1.98172 |
| 5 1.723 .1707159 5.49 0.000 1.418886 | 2.092294 |
| 6 1.361173 .1349994 3.11 0.002 1.120707 | 1.653234 |
| 7 1.382462 .1376333 3.25 0.001 1.137392 | 1.680335 |
| 8 1.264374 .1318501 2.25 0.024 1.030651 | 1.5511 |
| 9 1.401705 .1446948 3.27 0.001 1.144957 | 1.716027 |
| 10 1.620321 .1589259 4.92 0.000 1.336942 | 1.963765 |
| 11 1.645353 .1659201 4.94 0.000 1.350276 | 2.004913 |
| 12 1.789167 .2020266 5.15 0.000 1.433957 | 2.232367 |
| 13 1.482198 .1730269 3.37 0.001 1.179071 | 1.863257 |
| 14 1.539245 .1513055 4.39 0.000 1.269509 | 1.866292 |
| 15 1.540429 .1618563 4.11 0.000 1.25373 | 1.892689 |
| 16 1.508313 .1565505 3.96 0.000 1.230676 | 1.848585 |
| 17 1.611632 .1627903 4.72 0.000 1.322167 | 1.96447 |
| 18 1.259713 .1308368 2.22 0.026 1.027694 | 1.544114 |
| 19 1.683376 .1719939 5.10 0.000 1.377882 | 2.056601 |
| 20 1.638998 .1614635 5.02 0.000 1.351212 | 1.988077 |
| 21 1.315396 .1305839 2.76 0.006 1.082816 | 1.597932 |
| 22 1.518474 .1478577 4.29 0.000 1.254653 | 1.837769 |
| 23 1.493265 .1483887 4.03 0.000 1.228998 | 1.814355 |
| 24 1.406692 .1384977 3.47 0.001 1.159826 | 1.706103 |
| 25 1.472581 .1456612 3.91 0.000 1.21306 | 1.787625 |
| 26 1.427171 .1441577 3.52 0.000 1.170838 | 1.739624 |
| 27 1.434506 .1442682 3.59 0.000 1.177869 | 1.74706 |
| 28 1.595224 .1653078 4.51 0.000 1.302011 | 1.95447 |
| 29 1.242057 .1285744 2.09 0.036 1.013976 | 1.521443 |
| 30 1.572898 .1585795 4.49 0.000 1.29087 | 1.916544 |
| 31 1.598996 .1584518 4.74 0.000 1.316734 | 1.941766 |
| 32 1.697367 .1679352 5.35 0.000 1.398167 | 2.060594 |
| 33 1.391535 .1383881 3.32 0.001 1.145097 | 1.69101 |
| 34 1.58014 .1545772 4.68 0.000 1.304448 | 1.914098 |
| 35 1.688815 .1650863 5.36 0.000 1.39436 | 2.045452 |
| 36 1.647272 .1606651 5.12 0.000 1.360643 | 1.994281 |
| 37 1.597345 .1542089 4.85 0.000 1.321974 | 1.930076 |
| 38 .6388354 .0907847 -3.15 0.002 .4835317 | .8440205 |
| 39 1.466074 .1469467 3.82 0.000 1.204588 | 1.784321 |
| year |  |
| 2018 .9831849 .0184294 -0.90 0.366 .9477195 | 1.019977 |
| 2019 1.046706 .0208342 2.29 0.022 1.006658 | 1.088348 |
| 2020 1 (omitted) |  |
| _cons 1.88e-06 1.86e-07 -133.65 0.000 1.55e-06 | 2.29e-06 |
| ln(popula~n) 1 (exposure) |  |
|  |  |
| /lnalpha -4.609474 .2437528 -5.087221 | -4.131727 |
|  |  |
| alpha .0099571 .0024271 .0061752 | .0160551 |
|  |  |
| Note: Estimates are transformed only in the first equation. |  |
| Note: _cons estimates baseline incidence rate. |  |
| . |  |

**Table S19.- Gastric cancer model for both gender (full specification)**

| Negative binomial regression Number of obs = | 780 |
| --- | --- |
| Wald chi2(47) = | 5140.72 |
| Dispersion = mean Prob > chi2 = | 0.0000 |
| Log pseudolikelihood = -2551.1452 Pseudo R2 = | 0.2279 |
|  |  |
| Robust |  |
| _freq IRR Std. Err. z P>z [95% Conf. | Interval] |
|  |  |
| t2 .9999966 .0002541 -0.01 0.989 .9994987 | 1.000495 |
| 1.level .3064943 .0300701 -12.05 0.000 .2528782 | .3714784 |
| slope 1.021723 .0052582 4.18 0.000 1.011469 | 1.032081 |
| age_group |  |
| 4 1.702104 .0443439 20.42 0.000 1.617374 | 1.791274 |
| 5 2.666484 .0668696 39.11 0.000 2.538591 | 2.80082 |
| 6 3.758208 .0940063 52.93 0.000 3.578402 | 3.947048 |
| 7 2.986998 .0906899 36.04 0.000 2.814435 | 3.170143 |
| week |  |
| 2 1.618214 .1669463 4.67 0.000 1.321965 | 1.980851 |
| 3 1.464264 .1558189 3.58 0.000 1.18861 | 1.803847 |
| 4 1.39231 .1476807 3.12 0.002 1.130967 | 1.714043 |
| 5 1.477609 .1672419 3.45 0.001 1.183633 | 1.844599 |
| 6 1.177893 .136326 1.41 0.157 .9388372 | 1.47782 |
| 7 1.166926 .1269659 1.42 0.156 .9428217 | 1.4443 |
| 8 1.076878 .112078 0.71 0.477 .8781654 | 1.320556 |
| 9 1.168695 .1246314 1.46 0.144 .9482605 | 1.440371 |
| 10 1.52745 .1588411 4.07 0.000 1.245804 | 1.872769 |
| 11 1.57342 .1672333 4.26 0.000 1.277537 | 1.937831 |
| 12 1.614961 .2062358 3.75 0.000 1.257363 | 2.07426 |
| 13 1.304403 .1570658 2.21 0.027 1.030189 | 1.651607 |
| 14 1.438645 .1482598 3.53 0.000 1.175528 | 1.760655 |
| 15 1.277175 .14667 2.13 0.033 1.019762 | 1.599565 |
| 16 1.232122 .1325469 1.94 0.052 .9978952 | 1.521328 |
| 17 1.318695 .1393872 2.62 0.009 1.071943 | 1.622247 |
| 18 1.096036 .1253818 0.80 0.423 .8758927 | 1.371509 |
| 19 1.473464 .1597911 3.57 0.000 1.191325 | 1.822421 |
| 20 1.35363 .1424972 2.88 0.004 1.101269 | 1.663819 |
| 21 1.103605 .117849 0.92 0.356 .8951946 | 1.360534 |
| 22 1.360859 .1416712 2.96 0.003 1.109684 | 1.668887 |
| 23 1.306475 .1407032 2.48 0.013 1.057863 | 1.613514 |
| 24 1.16539 .1252708 1.42 0.154 .9440028 | 1.438696 |
| 25 1.290481 .1331887 2.47 0.013 1.054145 | 1.579802 |
| 26 1.188014 .1233661 1.66 0.097 .9692387 | 1.45617 |
| 27 1.227064 .1295749 1.94 0.053 .9976595 | 1.509218 |
| 28 1.341582 .1452402 2.71 0.007 1.085091 | 1.658703 |
| 29 .9715544 .1198978 -0.23 0.815 .7628196 | 1.237406 |
| 30 1.265696 .1375571 2.17 0.030 1.022869 | 1.56617 |
| 31 1.551949 .1587528 4.30 0.000 1.270006 | 1.896483 |
| 32 1.460459 .1566204 3.53 0.000 1.183603 | 1.802075 |
| 33 1.244871 .1300907 2.10 0.036 1.014314 | 1.527833 |
| 34 1.496377 .1680416 3.59 0.000 1.200748 | 1.86479 |
| 35 1.634414 .1682837 4.77 0.000 1.335734 | 1.999881 |
| 36 1.517035 .1538278 4.11 0.000 1.243608 | 1.850579 |
| 37 1.518264 .156803 4.04 0.000 1.240044 | 1.858907 |
| 38 .4862196 .0765307 -4.58 0.000 .3571526 | .6619287 |
| 39 1.367619 .1510397 2.83 0.005 1.101434 | 1.698133 |
| year |  |
| 2018 1.008032 .0220413 0.37 0.714 .9657449 | 1.052172 |
| 2019 1.008416 .0285151 0.30 0.767 .954048 | 1.065882 |
| 2020 1 (omitted) |  |
| _cons .0000109 1.04e-06 -120.44 0.000 9.08e-06 | .0000132 |
| ln(popula~n) 1 (exposure) |  |
|  |  |
| /lnalpha -4.167435 .2128203 -4.584556 | -3.750315 |
|  |  |
| alpha .0154919 .003297 .0102083 | .0235103 |
|  |  |
| Note: Estimates are transformed only in the first equation. |  |
| Note: _cons estimates baseline incidence rate. |  |

**Table S20.- Gastric cancer model for men (full specification)**

| Negative binomial regression Number of obs = | 780 |
| --- | --- |
| Wald chi2(47) = | 4220.93 |
| Dispersion = mean Prob > chi2 = | 0.0000 |
| Log pseudolikelihood = -2062.1773 Pseudo R2 = | 0.2544 |
|  |  |
| Robust |  |
| _freq IRR Std. Err. z P>z [95% Conf. | Interval] |
|  |  |
| t2 1.000166 .0003187 0.52 0.603 .9995413 | 1.00079 |
| 1.level .3376421 .0419337 -8.74 0.000 .264692 | .4306974 |
| slope 1.02138 .006735 3.21 0.001 1.008265 | 1.034666 |
| age_group |  |
| 4 1.90634 .0747178 16.46 0.000 1.765379 | 2.058556 |
| 5 3.569035 .1357679 33.45 0.000 3.312612 | 3.845306 |
| 6 5.671241 .2070888 47.53 0.000 5.279539 | 6.092005 |
| 7 5.756624 .2583221 39.01 0.000 5.271948 | 6.285858 |
| week |  |
| 2 1.526636 .1689011 3.82 0.000 1.229028 | 1.896309 |
| 3 1.396512 .1627209 2.87 0.004 1.111381 | 1.754794 |
| 4 1.343591 .1495824 2.65 0.008 1.080196 | 1.671213 |
| 5 1.390676 .1657226 2.77 0.006 1.101009 | 1.756552 |
| 6 1.155103 .1601333 1.04 0.298 .8802734 | 1.515737 |
| 7 1.104675 .1384195 0.79 0.427 .8641245 | 1.41219 |
| 8 1.112839 .1297102 0.92 0.359 .8855599 | 1.398449 |
| 9 1.186385 .1443966 1.40 0.160 .9345976 | 1.506005 |
| 10 1.613132 .1760338 4.38 0.000 1.302513 | 1.997826 |
| 11 1.434849 .1721998 3.01 0.003 1.1341 | 1.815352 |
| 12 1.477608 .1935546 2.98 0.003 1.143033 | 1.910116 |
| 13 1.215546 .1418717 1.67 0.094 .9669941 | 1.527984 |
| 14 1.44563 .1837392 2.90 0.004 1.12686 | 1.854576 |
| 15 1.139331 .1441294 1.03 0.302 .8891399 | 1.459923 |
| 16 1.177954 .138266 1.40 0.163 .9358708 | 1.482657 |
| 17 1.186111 .1430557 1.42 0.157 .9364033 | 1.502409 |
| 18 1.077973 .1381267 0.59 0.558 .8385686 | 1.385725 |
| 19 1.324193 .154782 2.40 0.016 1.053068 | 1.665123 |
| 20 1.265924 .1581285 1.89 0.059 .9910212 | 1.617084 |
| 21 1.015586 .1223249 0.13 0.898 .8020315 | 1.286002 |
| 22 1.241758 .1396895 1.92 0.054 .9960534 | 1.548073 |
| 23 1.233132 .138211 1.87 0.062 .9899334 | 1.536078 |
| 24 1.128567 .1442354 0.95 0.344 .8784969 | 1.449821 |
| 25 1.253107 .1433103 1.97 0.049 1.001478 | 1.56796 |
| 26 1.202221 .1450308 1.53 0.127 .949072 | 1.522894 |
| 27 1.149842 .1371314 1.17 0.242 .910171 | 1.452625 |
| 28 1.260918 .147377 1.98 0.047 1.002762 | 1.585535 |
| 29 .8781093 .1102182 -1.04 0.300 .6866063 | 1.123025 |
| 30 1.247578 .1462171 1.89 0.059 .9915308 | 1.569745 |
| 31 1.522379 .1796979 3.56 0.000 1.20795 | 1.918653 |
| 32 1.384416 .1622403 2.78 0.006 1.100307 | 1.741884 |
| 33 1.226028 .1491517 1.68 0.094 .9659349 | 1.556154 |
| 34 1.343405 .1612939 2.46 0.014 1.061717 | 1.699829 |
| 35 1.606342 .1779694 4.28 0.000 1.292801 | 1.995924 |
| 36 1.494455 .1653876 3.63 0.000 1.203047 | 1.85645 |
| 37 1.469784 .16654 3.40 0.001 1.177076 | 1.835281 |
| 38 .4476607 .0727023 -4.95 0.000 .3256189 | .6154435 |
| 39 1.378473 .1756293 2.52 0.012 1.07386 | 1.769492 |
| year |  |
| 2018 1.009808 .0290268 0.34 0.734 .9544898 | 1.068333 |
| 2019 .9713348 .0361113 -0.78 0.434 .9030751 | 1.044754 |
| 2020 1 (omitted) |  |
| _cons 7.69e-06 7.80e-07 -116.08 0.000 6.30e-06 | 9.38e-06 |
| ln(popula~n) 1 (exposure) |  |
|  |  |
| /lnalpha -5.106502 .7801154 -6.635501 | -3.577504 |
|  |  |
| alpha .0060572 .0047253 .0013129 | .0279454 |
|  |  |
| Note: Estimates are transformed only in the first equation. |  |
| Note: _cons estimates baseline incidence rate. |  |

**Table S21.- Gastric cancer model for women (full specification)**

| Negative binomial regression Number of obs = | 780 |
| --- | --- |
| Wald chi2(47) = | 2498.39 |
| Dispersion = mean Prob > chi2 = | 0.0000 |
| Log pseudolikelihood = -2275.779 Pseudo R2 = | 0.2014 |
|  |  |
| Robust |  |
| _freq IRR Std. Err. z P>z [95% Conf. | Interval] |
|  |  |
| t2 .9998898 .0002897 -0.38 0.704 .9993221 | 1.000458 |
| 1.level .2882305 .0325576 -11.01 0.000 .2309889 | .3596574 |
| slope 1.021206 .0060107 3.57 0.000 1.009493 | 1.033055 |
| age_group |  |
| 4 1.620885 .0484261 16.17 0.000 1.528697 | 1.718632 |
| 5 2.258224 .0661401 27.81 0.000 2.132243 | 2.39165 |
| 6 2.899067 .089048 34.65 0.000 2.729685 | 3.078958 |
| 7 1.936561 .0711316 17.99 0.000 1.802045 | 2.081117 |
| week |  |
| 2 1.688636 .1974714 4.48 0.000 1.34275 | 2.123619 |
| 3 1.514396 .1918099 3.28 0.001 1.181485 | 1.941113 |
| 4 1.443842 .1795396 2.95 0.003 1.131551 | 1.842321 |
| 5 1.559021 .1984904 3.49 0.000 1.214729 | 2.000897 |
| 6 1.193281 .148503 1.42 0.156 .934999 | 1.52291 |
| 7 1.21358 .1500445 1.57 0.117 .9524185 | 1.546355 |
| 8 1.052896 .1378433 0.39 0.694 .8146059 | 1.360891 |
| 9 1.169603 .1484465 1.23 0.217 .9120179 | 1.499938 |
| 10 1.470395 .1828556 3.10 0.002 1.152338 | 1.876237 |
| 11 1.678136 .2116257 4.11 0.000 1.310642 | 2.148672 |
| 12 1.669112 .2328007 3.67 0.000 1.269882 | 2.193853 |
| 13 1.377551 .2051477 2.15 0.031 1.028833 | 1.844464 |
| 14 1.444122 .1782598 2.98 0.003 1.133791 | 1.839393 |
| 15 1.382185 .1908218 2.34 0.019 1.05451 | 1.81168 |
| 16 1.282996 .1625137 1.97 0.049 1.000935 | 1.644541 |
| 17 1.419116 .1750922 2.84 0.005 1.114284 | 1.807342 |
| 18 1.109176 .145421 0.79 0.429 .8578315 | 1.434165 |
| 19 1.592444 .2027592 3.65 0.000 1.24075 | 2.043827 |
| 20 1.411589 .1796619 2.71 0.007 1.099944 | 1.811533 |
| 21 1.172332 .1443105 1.29 0.196 .9210223 | 1.492213 |
| 22 1.454497 .1814278 3.00 0.003 1.139037 | 1.857326 |
| 23 1.364437 .1736231 2.44 0.015 1.063259 | 1.750927 |
| 24 1.192079 .1506024 1.39 0.164 .9306099 | 1.527011 |
| 25 1.333879 .1637122 2.35 0.019 1.048686 | 1.696633 |
| 26 1.179565 .1397927 1.39 0.163 .9350701 | 1.487988 |
| 27 1.292873 .1616525 2.05 0.040 1.011876 | 1.651904 |
| 28 1.415578 .1784371 2.76 0.006 1.105701 | 1.812299 |
| 29 1.051164 .1544023 0.34 0.734 .7882068 | 1.401848 |
| 30 1.284904 .1737689 1.85 0.064 .9857238 | 1.67489 |
| 31 1.592919 .1854448 4.00 0.000 1.267939 | 2.001194 |
| 32 1.53839 .1970214 3.36 0.001 1.196887 | 1.977332 |
| 33 1.259414 .1510984 1.92 0.055 .9955096 | 1.593278 |
| 34 1.636136 .209511 3.84 0.000 1.272978 | 2.102895 |
| 35 1.667999 .2053138 4.16 0.000 1.310453 | 2.123098 |
| 36 1.533115 .185958 3.52 0.000 1.208729 | 1.944557 |
| 37 1.565104 .1881065 3.73 0.000 1.236628 | 1.98083 |
| 38 .5114528 .088367 -3.88 0.000 .3645339 | .7175846 |
| 39 1.365106 .1663229 2.55 0.011 1.07512 | 1.733308 |
| year |  |
| 2018 1.008434 .0269675 0.31 0.753 .9569397 | 1.062699 |
| 2019 1.036146 .0336974 1.09 0.275 .9721613 | 1.104342 |
| 2020 1 (omitted) |  |
| _cons .0000135 1.49e-06 -101.21 0.000 .0000108 | .0000167 |
| ln(popula~n) 1 (exposure) |  |
|  |  |
| /lnalpha -4.189677 .2636093 -4.706342 | -3.673012 |
|  |  |
| alpha .0151512 .003994 .0090378 | .0253998 |
|  |  |
| Note: Estimates are transformed only in the first equation. |  |
| Note: _cons estimates baseline incidence rate. |  |
| . |  |

**Table S22.- Colorectal cancer model for both gender (full specification)**

| Negative binomial regression Number of obs = | 1,092 |
| --- | --- |
| Wald chi2(49) = | 17091.25 |
| Dispersion = mean Prob > chi2 = | 0.0000 |
| Log pseudolikelihood = -3105.7714 Pseudo R2 = | 0.3513 |
|  |  |
| Robust |  |
| _freq IRR Std. Err. z P>z [95% Conf. | Interval] |
|  |  |
| t2 1.004795 .0001978 24.30 0.000 1.004408 | 1.005183 |
| 1.level .22948 .016746 -20.17 0.000 .1988976 | .2647646 |
| slope 1.035652 .0039033 9.29 0.000 1.02803 | 1.043331 |
| age_group |  |
| 2 2.61313 .168504 14.90 0.000 2.302886 | 2.96517 |
| 3 8.228409 .4880424 35.53 0.000 7.325369 | 9.242772 |
| 4 17.25731 .976225 50.35 0.000 15.4462 | 19.28078 |
| 5 31.80812 1.760517 62.51 0.000 28.53814 | 35.45278 |
| 6 51.36389 2.888137 70.05 0.000 46.00402 | 57.34824 |
| 7 51.28123 2.932303 68.86 0.000 45.84437 | 57.36286 |
| week |  |
| 2 1.844183 .2026724 5.57 0.000 1.48682 | 2.287439 |
| 3 1.903108 .2104767 5.82 0.000 1.532229 | 2.363759 |
| 4 1.953628 .2089844 6.26 0.000 1.584115 | 2.409334 |
| 5 1.954841 .2098277 6.24 0.000 1.583965 | 2.412556 |
| 6 1.684341 .1794284 4.89 0.000 1.366954 | 2.075421 |
| 7 1.685776 .183906 4.79 0.000 1.361256 | 2.08766 |
| 8 1.602716 .1820527 4.15 0.000 1.282828 | 2.002372 |
| 9 1.641671 .1813553 4.49 0.000 1.322069 | 2.038536 |
| 10 1.902141 .2047087 5.97 0.000 1.54041 | 2.348817 |
| 11 1.794957 .1936396 5.42 0.000 1.452869 | 2.217591 |
| 12 1.98606 .2431204 5.61 0.000 1.562406 | 2.52459 |
| 13 1.623946 .1901669 4.14 0.000 1.290905 | 2.042907 |
| 14 1.642135 .1792648 4.54 0.000 1.325827 | 2.033906 |
| 15 1.758367 .1893133 5.24 0.000 1.423854 | 2.171468 |
| 16 1.78633 .1938945 5.34 0.000 1.444007 | 2.209805 |
| 17 1.833369 .2116751 5.25 0.000 1.462086 | 2.298937 |
| 18 1.46357 .1652809 3.37 0.001 1.172971 | 1.826164 |
| 19 1.767696 .193854 5.19 0.000 1.425807 | 2.191565 |
| 20 1.84214 .1940958 5.80 0.000 1.49843 | 2.264691 |
| 21 1.573277 .1708152 4.17 0.000 1.271709 | 1.946358 |
| 22 1.737117 .1870901 5.13 0.000 1.406544 | 2.145383 |
| 23 1.680787 .1827832 4.77 0.000 1.358144 | 2.080078 |
| 24 1.709482 .190548 4.81 0.000 1.373995 | 2.126885 |
| 25 1.786609 .195177 5.31 0.000 1.442249 | 2.213189 |
| 26 1.788671 .2044175 5.09 0.000 1.429721 | 2.23774 |
| 27 1.741955 .2006001 4.82 0.000 1.389998 | 2.18303 |
| 28 1.858935 .2029727 5.68 0.000 1.500802 | 2.302527 |
| 29 1.549908 .162734 4.17 0.000 1.261634 | 1.904051 |
| 30 1.990384 .2201267 6.22 0.000 1.6025 | 2.472154 |
| 31 1.82311 .2038086 5.37 0.000 1.464386 | 2.269708 |
| 32 1.960996 .2183618 6.05 0.000 1.576498 | 2.439271 |
| 33 1.521244 .1713227 3.73 0.000 1.219935 | 1.896974 |
| 34 1.755203 .1905561 5.18 0.000 1.418781 | 2.171397 |
| 35 1.78813 .1886996 5.51 0.000 1.454027 | 2.199001 |
| 36 1.847642 .199131 5.70 0.000 1.495819 | 2.282216 |
| 37 1.835802 .1981192 5.63 0.000 1.485814 | 2.268231 |
| 38 .7903363 .1134886 -1.64 0.101 .5964628 | 1.047226 |
| 39 1.672426 .1821373 4.72 0.000 1.35097 | 2.07037 |
| year |  |
| 2018 1.032465 .0218109 1.51 0.130 .990589 | 1.07611 |
| 2019 1.093528 .0230119 4.25 0.000 1.049343 | 1.139574 |
| 2020 1 (omitted) |  |
| _cons 4.39e-07 4.96e-08 -129.57 0.000 3.52e-07 | 5.47e-07 |
| ln(popula~n) 1 (exposure) |  |
|  |  |
| /lnalpha -4.730868 .292718 -5.304585 | -4.157151 |
|  |  |
| alpha .0088188 .0025814 .0049688 | .0156521 |
|  |  |
| Note: Estimates are transformed only in the first equation. |  |
| Note: _cons estimates baseline incidence rate. |  |
| . |  |

**Table S23.- Colorectal cancer model for men (full specification)**

| Negative binomial regression Number of obs = | 1,092 |
| --- | --- |
| Wald chi2(49) = | 8827.50 |
| Dispersion = mean Prob > chi2 = | 0.0000 |
| Log pseudolikelihood = -2491.0471 Pseudo R2 = | 0.3564 |
|  |  |
| Robust |  |
| _freq IRR Std. Err. z P>z [95% Conf. | Interval] |
|  |  |
| t2 1.004327 .0002808 15.44 0.000 1.003777 | 1.004878 |
| 1.level .2947705 .0260184 -13.84 0.000 .2479427 | .3504426 |
| slope 1.032297 .0045929 7.14 0.000 1.023334 | 1.041338 |
| age_group |  |
| 2 2.703356 .2932882 9.17 0.000 2.185526 | 3.343878 |
| 3 8.218947 .7742654 22.36 0.000 6.833274 | 9.885612 |
| 4 17.76091 1.619837 31.55 0.000 14.85367 | 21.23718 |
| 5 35.93018 3.219416 39.97 0.000 30.14325 | 42.8281 |
| 6 64.0383 5.757816 46.26 0.000 53.69162 | 76.37884 |
| 7 72.19943 6.63643 46.56 0.000 60.29663 | 86.4519 |
| week |  |
| 2 1.828568 .2600848 4.24 0.000 1.383697 | 2.416468 |
| 3 1.937775 .2684227 4.78 0.000 1.477046 | 2.542218 |
| 4 2.001026 .264135 5.26 0.000 1.544879 | 2.591858 |
| 5 1.943781 .2547042 5.07 0.000 1.503522 | 2.512956 |
| 6 1.89551 .2389669 5.07 0.000 1.480522 | 2.426817 |
| 7 1.836408 .2547234 4.38 0.000 1.399269 | 2.410111 |
| 8 1.71761 .2326971 3.99 0.000 1.317062 | 2.239975 |
| 9 1.645237 .2220205 3.69 0.000 1.262877 | 2.143364 |
| 10 1.998883 .2556777 5.41 0.000 1.555643 | 2.568413 |
| 11 1.960168 .2670055 4.94 0.000 1.500881 | 2.560001 |
| 12 1.958605 .2772199 4.75 0.000 1.484118 | 2.584792 |
| 13 1.667187 .2373363 3.59 0.000 1.261274 | 2.203734 |
| 14 1.578098 .2187727 3.29 0.001 1.202628 | 2.070792 |
| 15 1.802666 .2396451 4.43 0.000 1.389175 | 2.339232 |
| 16 1.791274 .2514672 4.15 0.000 1.3604 | 2.358618 |
| 17 1.787892 .2649782 3.92 0.000 1.337172 | 2.390536 |
| 18 1.509317 .2013207 3.09 0.002 1.162098 | 1.96028 |
| 19 1.602846 .2293393 3.30 0.001 1.210876 | 2.1217 |
| 20 1.753872 .2293486 4.30 0.000 1.357341 | 2.266246 |
| 21 1.588345 .2057511 3.57 0.000 1.232202 | 2.047424 |
| 22 1.892791 .2573703 4.69 0.000 1.449978 | 2.470836 |
| 23 1.696426 .2433638 3.68 0.000 1.280632 | 2.24722 |
| 24 1.859888 .2517906 4.58 0.000 1.426434 | 2.425057 |
| 25 1.893582 .2666221 4.53 0.000 1.436919 | 2.495375 |
| 26 1.833816 .2654656 4.19 0.000 1.380813 | 2.435437 |
| 27 1.838777 .2593345 4.32 0.000 1.394693 | 2.424261 |
| 28 1.91829 .2549073 4.90 0.000 1.478443 | 2.488995 |
| 29 1.642293 .2135549 3.82 0.000 1.272814 | 2.119026 |
| 30 1.923224 .2489138 5.05 0.000 1.492323 | 2.478546 |
| 31 1.952914 .2701294 4.84 0.000 1.489169 | 2.561075 |
| 32 1.963749 .2583348 5.13 0.000 1.517431 | 2.541342 |
| 33 1.479078 .2154666 2.69 0.007 1.111709 | 1.967845 |
| 34 1.96462 .2628269 5.05 0.000 1.511488 | 2.553596 |
| 35 1.730421 .2393801 3.96 0.000 1.31947 | 2.269362 |
| 36 1.718297 .2304584 4.04 0.000 1.321097 | 2.234918 |
| 37 2.009873 .2665949 5.26 0.000 1.549755 | 2.606599 |
| 38 .8216724 .139605 -1.16 0.248 .5889475 | 1.146359 |
| 39 1.764868 .2359565 4.25 0.000 1.358031 | 2.293585 |
| year |  |
| 2018 1.089756 .0313156 2.99 0.003 1.030075 | 1.152895 |
| 2019 1.132903 .0361985 3.91 0.000 1.064132 | 1.20612 |
| 2020 1 (omitted) |  |
| _cons 3.45e-07 5.11e-08 -100.35 0.000 2.58e-07 | 4.61e-07 |
| ln(popula~n) 1 (exposure) |  |
|  |  |
| /lnalpha -5.347954 .826045 -6.966972 | -3.728936 |
|  |  |
| alpha .0047579 .0039302 .0009425 | .0240184 |
|  |  |
| Note: Estimates are transformed only in the first equation. |  |
| Note: _cons estimates baseline incidence rate. |  |

**Table S24.- Colorectal cancer model for women (full specification)**

| Negative binomial regression Number of obs = | 1,092 |
| --- | --- |
| Wald chi2(49) = | 10316.22 |
| Dispersion = mean Prob > chi2 = | 0.0000 |
| Log pseudolikelihood = -2784.5298 Pseudo R2 = | 0.3350 |
|  |  |
| Robust |  |
| _freq IRR Std. Err. z P>z [95% Conf. | Interval] |
|  |  |
| t2 1.005087 .0002537 20.10 0.000 1.00459 | 1.005585 |
| 1.level .1900596 .0175342 -18.00 0.000 .1586212 | .227729 |
| slope 1.038435 .0048772 8.03 0.000 1.02892 | 1.048039 |
| age_group |  |
| 2 2.533793 .1924071 12.24 0.000 2.183403 | 2.940413 |
| 3 8.192631 .5491447 31.38 0.000 7.18403 | 9.342835 |
| 4 16.95895 1.07392 44.70 0.000 14.97948 | 19.19999 |
| 5 29.18689 1.815191 54.25 0.000 25.83747 | 32.97052 |
| 6 43.60396 2.742972 60.01 0.000 38.54605 | 49.32556 |
| 7 40.32096 2.598846 57.36 0.000 35.53592 | 45.75032 |
| week |  |
| 2 1.869692 .2205441 5.31 0.000 1.483763 | 2.356002 |
| 3 1.904562 .2351387 5.22 0.000 1.49522 | 2.425969 |
| 4 1.949498 .2281326 5.70 0.000 1.549938 | 2.452062 |
| 5 1.988156 .2372909 5.76 0.000 1.573467 | 2.512136 |
| 6 1.568276 .1915146 3.68 0.000 1.234456 | 1.992368 |
| 7 1.605234 .2012608 3.77 0.000 1.255499 | 2.052391 |
| 8 1.545959 .2000921 3.37 0.001 1.199576 | 1.992362 |
| 9 1.654181 .2108628 3.95 0.000 1.288481 | 2.123674 |
| 10 1.851895 .2197736 5.19 0.000 1.467574 | 2.33686 |
| 11 1.703134 .2012565 4.51 0.000 1.351026 | 2.147009 |
| 12 2.009352 .2712873 5.17 0.000 1.542174 | 2.618054 |
| 13 1.62406 .2083363 3.78 0.000 1.263016 | 2.088311 |
| 14 1.704848 .2089842 4.35 0.000 1.340737 | 2.167844 |
| 15 1.758095 .2152669 4.61 0.000 1.382988 | 2.234942 |
| 16 1.799968 .2272919 4.65 0.000 1.405332 | 2.305423 |
| 17 1.88539 .2383963 5.02 0.000 1.471539 | 2.41563 |
| 18 1.455401 .1926612 2.83 0.005 1.122803 | 1.886522 |
| 19 1.901722 .2379761 5.14 0.000 1.488093 | 2.430323 |
| 20 1.922351 .2244627 5.60 0.000 1.529123 | 2.416701 |
| 21 1.576621 .196345 3.66 0.000 1.235159 | 2.012482 |
| 22 1.652265 .1987095 4.18 0.000 1.305299 | 2.09146 |
| 23 1.695511 .2055033 4.36 0.000 1.336999 | 2.150156 |
| 24 1.630385 .2052627 3.88 0.000 1.273871 | 2.086676 |
| 25 1.746831 .2118294 4.60 0.000 1.377305 | 2.2155 |
| 26 1.784147 .2277249 4.54 0.000 1.389265 | 2.29127 |
| 27 1.697406 .2135495 4.21 0.000 1.326467 | 2.172075 |
| 28 1.845254 .2295777 4.92 0.000 1.445952 | 2.354825 |
| 29 1.500214 .1770239 3.44 0.001 1.190453 | 1.890576 |
| 30 2.059275 .2535921 5.87 0.000 1.617678 | 2.621421 |
| 31 1.75214 .2182889 4.50 0.000 1.372532 | 2.236737 |
| 32 1.980218 .2441545 5.54 0.000 1.555115 | 2.521525 |
| 33 1.575707 .2000399 3.58 0.000 1.228608 | 2.020867 |
| 34 1.62151 .1929173 4.06 0.000 1.284248 | 2.047342 |
| 35 1.838684 .2170007 5.16 0.000 1.458977 | 2.31721 |
| 36 1.957095 .233394 5.63 0.000 1.549179 | 2.472419 |
| 37 1.721348 .212585 4.40 0.000 1.351282 | 2.192761 |
| 38 .7718234 .121323 -1.65 0.099 .5671755 | 1.050312 |
| 39 1.638614 .2107693 3.84 0.000 1.273472 | 2.108452 |
| year |  |
| 2018 .9945658 .0268171 -0.20 0.840 .9433699 | 1.04854 |
| 2019 1.066422 .028265 2.43 0.015 1.012438 | 1.123284 |
| 2020 1 (omitted) |  |
| _cons 5.19e-07 6.45e-08 -116.34 0.000 4.07e-07 | 6.62e-07 |
| ln(popula~n) 1 (exposure) |  |
|  |  |
| /lnalpha -4.475144 .3360837 -5.133856 | -3.816432 |
|  |  |
| alpha .0113886 .0038275 .0058938 | .0220062 |
|  |  |
| Note: Estimates are transformed only in the first equation. |  |
| Note: _cons estimates baseline incidence rate. |  |
| . |  |

**Table S25.- Lymphoma model for both gender (full specification)**

| Negative binomial regression Number of obs = | 1,092 |
| --- | --- |
| Wald chi2(49) = | 2331.70 |
| Dispersion = mean Prob > chi2 = | 0.0000 |
| Log pseudolikelihood = -2331.7523 Pseudo R2 = | 0.1995 |
|  |  |
| Robust |  |
| _freq IRR Std. Err. z P>z [95% Conf. | Interval] |
|  |  |
| t2 1.000683 .0003789 1.80 0.071 .9999406 | 1.001426 |
| 1.level .5688832 .0573084 -5.60 0.000 .4669543 | .6930614 |
| slope 1.016734 .0051097 3.30 0.001 1.006769 | 1.026799 |
| age_group |  |
| 2 1.165027 .0715723 2.49 0.013 1.032864 | 1.314101 |
| 3 1.538372 .0904011 7.33 0.000 1.371012 | 1.726161 |
| 4 2.226514 .1190923 14.96 0.000 2.004916 | 2.472604 |
| 5 3.943079 .1959645 27.61 0.000 3.577109 | 4.346491 |
| 6 5.305395 .2842284 31.15 0.000 4.776567 | 5.89277 |
| 7 4.012902 .2494031 22.36 0.000 3.55268 | 4.532742 |
| week |  |
| 2 1.61758 .1951622 3.99 0.000 1.276932 | 2.049103 |
| 3 1.333158 .1595006 2.40 0.016 1.054491 | 1.685467 |
| 4 1.305145 .1755105 1.98 0.048 1.00275 | 1.698731 |
| 5 1.277192 .1570101 1.99 0.047 1.003725 | 1.625166 |
| 6 1.205315 .1496516 1.50 0.133 .9449645 | 1.537395 |
| 7 1.17754 .1363913 1.41 0.158 .9383901 | 1.477639 |
| 8 1.035725 .1372749 0.26 0.791 .7987788 | 1.342959 |
| 9 1.429039 .1753815 2.91 0.004 1.123515 | 1.817647 |
| 10 1.436126 .1733817 3.00 0.003 1.133517 | 1.81952 |
| 11 1.277007 .1421992 2.20 0.028 1.026618 | 1.588464 |
| 12 1.236396 .1644785 1.60 0.111 .9526249 | 1.604699 |
| 13 1.182093 .1569329 1.26 0.208 .9112712 | 1.533402 |
| 14 1.098738 .1426895 0.73 0.468 .8518269 | 1.417219 |
| 15 1.279783 .1543567 2.05 0.041 1.01035 | 1.621068 |
| 16 1.245219 .1799581 1.52 0.129 .9380607 | 1.652954 |
| 17 1.520864 .1946001 3.28 0.001 1.183521 | 1.954361 |
| 18 .9163225 .1427953 -0.56 0.575 .6751515 | 1.243642 |
| 19 1.392553 .1647551 2.80 0.005 1.104345 | 1.755976 |
| 20 1.319537 .1666704 2.20 0.028 1.030164 | 1.690194 |
| 21 1.009086 .1477523 0.06 0.951 .7573451 | 1.344506 |
| 22 1.34578 .1691229 2.36 0.018 1.051972 | 1.721645 |
| 23 1.179338 .1491421 1.30 0.192 .9204352 | 1.511066 |
| 24 1.380609 .1723919 2.58 0.010 1.080897 | 1.763427 |
| 25 1.215352 .1760238 1.35 0.178 .9149968 | 1.614302 |
| 26 1.191118 .1666449 1.25 0.211 .9054538 | 1.566907 |
| 27 1.241125 .1836627 1.46 0.144 .928654 | 1.658735 |
| 28 1.309021 .1619488 2.18 0.030 1.02716 | 1.668227 |
| 29 .981675 .1421225 -0.13 0.898 .7391535 | 1.30377 |
| 30 1.150433 .1803479 0.89 0.371 .846102 | 1.564229 |
| 31 1.16302 .1437805 1.22 0.222 .9127583 | 1.481899 |
| 32 1.555152 .1741717 3.94 0.000 1.248651 | 1.936888 |
| 33 1.151591 .1528478 1.06 0.288 .8878111 | 1.493744 |
| 34 1.235356 .1537681 1.70 0.090 .9679229 | 1.57668 |
| 35 1.344912 .1535278 2.60 0.009 1.07529 | 1.682141 |
| 36 1.276032 .1654641 1.88 0.060 .9896589 | 1.645271 |
| 37 1.392892 .156101 2.96 0.003 1.11821 | 1.735047 |
| 38 .5612767 .0973374 -3.33 0.001 .3995402 | .7884852 |
| 39 1.456909 .1782575 3.08 0.002 1.146265 | 1.851738 |
| year |  |
| 2018 .960493 .0363634 -1.06 0.287 .8918021 | 1.034475 |
| 2019 1.073826 .0462541 1.65 0.098 .9868909 | 1.168419 |
| 2020 1 (omitted) |  |
| _cons 1.32e-06 1.45e-07 -123.06 0.000 1.06e-06 | 1.63e-06 |
| ln(popula~n) 1 (exposure) |  |
|  |  |
| /lnalpha -16.62365 .309268 -17.2298 | -16.01749 |
|  |  |
| alpha 6.03e-08 1.87e-08 3.29e-08 | 1.11e-07 |
|  |  |
| Note: Estimates are transformed only in the first equation. |  |
| Note: _cons estimates baseline incidence rate. |  |
| . |  |

**Table S26.- Lymphoma model for men (full specification)**

| Negative binomial regression Number of obs = | 1,092 |
| --- | --- |
| Wald chi2(49) = | 1168.77 |
| Dispersion = mean Prob > chi2 = | 0.0000 |
| Log pseudolikelihood = -1923.1854 Pseudo R2 = | 0.1584 |
|  |  |
| Robust |  |
| _freq IRR Std. Err. z P>z [95% Conf. | Interval] |
|  |  |
| t2 1.000928 .0005847 1.59 0.112 .999783 | 1.002075 |
| 1.level .6430993 .0890765 -3.19 0.001 .4902042 | .8436826 |
| slope 1.00916 .0065527 1.40 0.160 .9963983 | 1.022085 |
| age_group |  |
| 2 1.20121 .0922722 2.39 0.017 1.033315 | 1.396383 |
| 3 1.467682 .110906 5.08 0.000 1.265641 | 1.701976 |
| 4 1.984223 .1383486 9.83 0.000 1.730777 | 2.274783 |
| 5 3.433401 .2369473 17.87 0.000 2.999032 | 3.930683 |
| 6 4.915582 .3518362 22.25 0.000 4.272181 | 5.655881 |
| 7 4.188031 .3678501 16.31 0.000 3.525703 | 4.974782 |
| week |  |
| 2 1.417383 .2366517 2.09 0.037 1.021804 | 1.966107 |
| 3 1.125898 .2062515 0.65 0.517 .786265 | 1.612239 |
| 4 1.092211 .1814532 0.53 0.595 .7886662 | 1.512586 |
| 5 1.042576 .2048258 0.21 0.832 .7093813 | 1.532272 |
| 6 1.057147 .2014821 0.29 0.771 .7276194 | 1.535911 |
| 7 1.215631 .1967322 1.21 0.228 .8852114 | 1.669384 |
| 8 .9583764 .1722572 -0.24 0.813 .6738176 | 1.363107 |
| 9 1.403668 .227316 2.09 0.036 1.021921 | 1.928021 |
| 10 1.353935 .228931 1.79 0.073 .9720129 | 1.885923 |
| 11 1.192992 .2168541 0.97 0.332 .8354333 | 1.703583 |
| 12 1.181295 .2078215 0.95 0.344 .8367757 | 1.66766 |
| 13 .8963139 .1640116 -0.60 0.550 .626186 | 1.282971 |
| 14 .9635037 .1565916 -0.23 0.819 .70067 | 1.324931 |
| 15 1.152471 .2016574 0.81 0.417 .8178776 | 1.623947 |
| 16 1.0269 .1931587 0.14 0.888 .710261 | 1.484698 |
| 17 1.509313 .2495413 2.49 0.013 1.091557 | 2.08695 |
| 18 .7608359 .1594343 -1.30 0.192 .5045689 | 1.147259 |
| 19 1.413269 .2204354 2.22 0.027 1.041018 | 1.918631 |
| 20 .9791584 .15425 -0.13 0.894 .7190522 | 1.333354 |
| 21 1.010162 .1877488 0.05 0.957 .7017572 | 1.454104 |
| 22 1.348099 .2230246 1.81 0.071 .97477 | 1.86441 |
| 23 1.020459 .1612497 0.13 0.898 .7486717 | 1.390912 |
| 24 1.101797 .1878679 0.57 0.570 .7887924 | 1.539006 |
| 25 1.199456 .1904261 1.15 0.252 .8787131 | 1.637274 |
| 26 1.094388 .2107144 0.47 0.639 .750378 | 1.596108 |
| 27 1.124238 .2133904 0.62 0.537 .7749842 | 1.630888 |
| 28 1.137137 .2157776 0.68 0.498 .783958 | 1.649425 |
| 29 .9665978 .1989249 -0.17 0.869 .6457573 | 1.446846 |
| 30 1.229016 .2424281 1.05 0.296 .8349387 | 1.80909 |
| 31 1.158579 .1917303 0.89 0.374 .8376498 | 1.602466 |
| 32 1.550427 .2481299 2.74 0.006 1.132988 | 2.121667 |
| 33 1.101255 .2036117 0.52 0.602 .7664941 | 1.582221 |
| 34 1.081037 .1927287 0.44 0.662 .7622318 | 1.533182 |
| 35 1.403694 .2273598 2.09 0.036 1.021883 | 1.928162 |
| 36 1.284908 .221458 1.45 0.146 .9165682 | 1.801272 |
| 37 1.296581 .196209 1.72 0.086 .9638047 | 1.744256 |
| 38 .4683452 .1143329 -3.11 0.002 .2902485 | .7557223 |
| 39 1.560962 .2439671 2.85 0.004 1.149094 | 2.120454 |
| year |  |
| 2018 .9484197 .0511272 -0.98 0.326 .8533244 | 1.054112 |
| 2019 1.052251 .0686925 0.78 0.435 .9258736 | 1.195879 |
| 2020 1 (omitted) |  |
| _cons 1.70e-06 2.48e-07 -91.19 0.000 1.28e-06 | 2.27e-06 |
| ln(popula~n) 1 (exposure) |  |
|  |  |
| /lnalpha -16.61673 .3229799 -17.24976 | -15.9837 |
|  |  |
| alpha 6.07e-08 1.96e-08 3.22e-08 | 1.14e-07 |
|  |  |
| Note: Estimates are transformed only in the first equation. |  |
| Note: _cons estimates baseline incidence rate. |  |

**Table S27.- Lymphoma model for women (full specification)**

| Negative binomial regression Number of obs = | 1,092 |
| --- | --- |
| Wald chi2(49) = | 1302.36 |
| Dispersion = mean Prob > chi2 = | 0.0000 |
| Log pseudolikelihood = -1871.7331 Pseudo R2 = | 0.1794 |
|  |  |
| Robust |  |
| _freq IRR Std. Err. z P>z [95% Conf. | Interval] |
|  |  |
| t2 1.000437 .0005275 0.83 0.408 .9994033 | 1.001471 |
| 1.level .4973269 .0698375 -4.97 0.000 .3776691 | .6548963 |
| slope 1.025019 .0073333 3.45 0.001 1.010746 | 1.039493 |
| age_group |  |
| 2 1.142183 .1049667 1.45 0.148 .9539158 | 1.367606 |
| 3 1.645416 .1382746 5.93 0.000 1.395545 | 1.940026 |
| 4 2.55238 .1996202 11.98 0.000 2.189643 | 2.975208 |
| 5 4.635228 .3348258 21.23 0.000 4.02332 | 5.340202 |
| 6 5.921335 .4440906 23.71 0.000 5.111883 | 6.858962 |
| 7 4.205833 .3608149 16.74 0.000 3.554906 | 4.975948 |
| week |  |
| 2 1.860984 .3226718 3.58 0.000 1.324813 | 2.61415 |
| 3 1.585234 .2704929 2.70 0.007 1.13462 | 2.214809 |
| 4 1.564183 .2896009 2.42 0.016 1.088158 | 2.248451 |
| 5 1.562706 .2667017 2.62 0.009 1.118422 | 2.183479 |
| 6 1.385592 .2377462 1.90 0.057 .9898798 | 1.939494 |
| 7 1.130823 .2014316 0.69 0.490 .7975752 | 1.603311 |
| 8 1.129756 .2086265 0.66 0.509 .7866787 | 1.622452 |
| 9 1.459513 .2642548 2.09 0.037 1.02351 | 2.081248 |
| 10 1.535903 .2808949 2.35 0.019 1.073226 | 2.198045 |
| 11 1.379067 .2333931 1.90 0.058 .9897557 | 1.921509 |
| 12 1.2977 .2297335 1.47 0.141 .9172447 | 1.835962 |
| 13 1.536528 .2870274 2.30 0.021 1.065452 | 2.215884 |
| 14 1.263213 .2469697 1.20 0.232 .8611108 | 1.853081 |
| 15 1.433478 .2594527 1.99 0.047 1.005374 | 2.043875 |
| 16 1.514143 .3174509 1.98 0.048 1.003937 | 2.283638 |
| 17 1.52834 .2929714 2.21 0.027 1.049665 | 2.225302 |
| 18 1.107796 .2177793 0.52 0.603 .75357 | 1.62853 |
| 19 1.361319 .2551427 1.65 0.100 .9428119 | 1.965599 |
| 20 1.740991 .3324442 2.90 0.004 1.197454 | 2.531247 |
| 21 1.004762 .1980419 0.02 0.981 .6827924 | 1.478557 |
| 22 1.339431 .240905 1.62 0.104 .9415137 | 1.905524 |
| 23 1.374257 .2837475 1.54 0.124 .916891 | 2.059769 |
| 24 1.723865 .3069359 3.06 0.002 1.216035 | 2.443771 |
| 25 1.233204 .2739715 0.94 0.345 .7978664 | 1.906075 |
| 26 1.309108 .2404247 1.47 0.142 .913372 | 1.876304 |
| 27 1.383946 .2613992 1.72 0.085 .9557506 | 2.003982 |
| 28 1.519298 .2959884 2.15 0.032 1.037081 | 2.225735 |
| 29 .9998406 .1978666 -0.00 0.999 .6783903 | 1.473608 |
| 30 1.054429 .2211986 0.25 0.801 .6989594 | 1.59068 |
| 31 1.168629 .2213767 0.82 0.411 .8061781 | 1.694034 |
| 32 1.561466 .2657535 2.62 0.009 1.118568 | 2.17973 |
| 33 1.21314 .2411843 0.97 0.331 .8216439 | 1.791176 |
| 34 1.422363 .2499424 2.00 0.045 1.007939 | 2.007181 |
| 35 1.275383 .2301543 1.35 0.178 .8954339 | 1.816551 |
| 36 1.266608 .2331967 1.28 0.199 .8829304 | 1.817013 |
| 37 1.509304 .2557514 2.43 0.015 1.082782 | 2.10384 |
| 38 .6724414 .1352133 -1.97 0.048 .4534155 | .9972694 |
| 39 1.335176 .2468381 1.56 0.118 .9293395 | 1.91824 |
| year |  |
| 2018 .9729919 .0510795 -0.52 0.602 .8778563 | 1.078438 |
| 2019 1.096072 .0665926 1.51 0.131 .9730248 | 1.23468 |
| 2020 1 (omitted) |  |
| _cons 9.91e-07 1.65e-07 -82.81 0.000 7.14e-07 | 1.37e-06 |
| ln(popula~n) 1 (exposure) |  |
|  |  |
| /lnalpha -16.12427 .3955983 -16.89962 | -15.34891 |
|  |  |
| alpha 9.94e-08 3.93e-08 4.58e-08 | 2.16e-07 |
|  |  |
| Note: Estimates are transformed only in the first equation. |  |
| Note: _cons estimates baseline incidence rate. |  |
| . |  |

**Table S28.- Leukemia model for both gender (full specification)**

| Negative binomial regression Number of obs = | 1,092 |
| --- | --- |
| Wald chi2(49) = | 2083.43 |
| Dispersion = mean Prob > chi2 = | 0.0000 |
| Log pseudolikelihood = -1838.9741 Pseudo R2 = | 0.2388 |
|  |  |
| Robust |  |
| _freq IRR Std. Err. z P>z [95% Conf. | Interval] |
|  |  |
| t2 1.000824 .0005336 1.55 0.122 .999779 | 1.001871 |
| 1.level .3878099 .0604572 -6.08 0.000 .2857075 | .5264001 |
| slope 1.031018 .0082989 3.79 0.000 1.01488 | 1.047412 |
| age_group |  |
| 2 1.141129 .1172221 1.29 0.199 .9330293 | 1.395642 |
| 3 1.790391 .1648422 6.33 0.000 1.49478 | 2.144462 |
| 4 3.185608 .2542526 14.52 0.000 2.724303 | 3.725025 |
| 5 5.49289 .4359127 21.47 0.000 4.701647 | 6.417292 |
| 6 9.190318 .7109793 28.67 0.000 7.897326 | 10.69501 |
| 7 11.22396 .9230633 29.40 0.000 9.55307 | 13.1871 |
| week |  |
| 2 1.973585 .3735184 3.59 0.000 1.361941 | 2.859916 |
| 3 2.255944 .4192717 4.38 0.000 1.567223 | 3.247326 |
| 4 1.660084 .3496079 2.41 0.016 1.098677 | 2.508361 |
| 5 2.131582 .4294813 3.76 0.000 1.436144 | 3.163779 |
| 6 1.537448 .2949389 2.24 0.025 1.055623 | 2.239196 |
| 7 1.913372 .3567542 3.48 0.001 1.327671 | 2.757456 |
| 8 1.580582 .3227664 2.24 0.025 1.059242 | 2.358514 |
| 9 1.62562 .3255538 2.43 0.015 1.097877 | 2.407046 |
| 10 1.741123 .3498701 2.76 0.006 1.174315 | 2.581513 |
| 11 1.691836 .3219614 2.76 0.006 1.165124 | 2.456656 |
| 12 2.07253 .3865006 3.91 0.000 1.438012 | 2.987027 |
| 13 1.581254 .3352751 2.16 0.031 1.043567 | 2.395978 |
| 14 2.218343 .4053429 4.36 0.000 1.550581 | 3.17368 |
| 15 2.094126 .4129458 3.75 0.000 1.422827 | 3.082146 |
| 16 1.443561 .2859638 1.85 0.064 .9790739 | 2.128408 |
| 17 2.180211 .4290618 3.96 0.000 1.482462 | 3.20637 |
| 18 1.783089 .3264517 3.16 0.002 1.24547 | 2.552776 |
| 19 1.935767 .3795514 3.37 0.001 1.318122 | 2.842828 |
| 20 1.598557 .3194681 2.35 0.019 1.080482 | 2.365042 |
| 21 1.58838 .3492714 2.10 0.035 1.032244 | 2.444142 |
| 22 1.471077 .2798669 2.03 0.042 1.013205 | 2.135863 |
| 23 1.541038 .3063866 2.18 0.030 1.043708 | 2.275348 |
| 24 1.557013 .3263058 2.11 0.035 1.032534 | 2.347902 |
| 25 1.415235 .2890799 1.70 0.089 .9483309 | 2.112017 |
| 26 1.431303 .2826633 1.82 0.069 .9719205 | 2.107816 |
| 27 1.188499 .2672514 0.77 0.443 .7648803 | 1.846732 |
| 28 1.667107 .356387 2.39 0.017 1.096471 | 2.534718 |
| 29 1.629201 .3175093 2.50 0.012 1.111955 | 2.387055 |
| 30 1.793628 .3778415 2.77 0.006 1.186917 | 2.710469 |
| 31 1.4789 .3033283 1.91 0.056 .989359 | 2.210668 |
| 32 1.367711 .2792917 1.53 0.125 .9165913 | 2.040858 |
| 33 1.677322 .3461226 2.51 0.012 1.119353 | 2.513422 |
| 34 1.590107 .331863 2.22 0.026 1.056274 | 2.393736 |
| 35 1.576742 .2949278 2.43 0.015 1.092809 | 2.274976 |
| 36 1.44298 .3254759 1.63 0.104 .9273949 | 2.245203 |
| 37 1.454209 .3035654 1.79 0.073 .9659151 | 2.189348 |
| 38 .7088126 .1770134 -1.38 0.168 .4344691 | 1.156389 |
| 39 1.61546 .3228275 2.40 0.016 1.091932 | 2.389996 |
| year |  |
| 2018 .9036722 .047329 -1.93 0.053 .8155113 | 1.001364 |
| 2019 .9948937 .0609076 -0.08 0.933 .8824009 | 1.121728 |
| 2020 1 (omitted) |  |
| _cons 3.41e-07 6.21e-08 -81.72 0.000 2.38e-07 | 4.87e-07 |
| ln(popula~n) 1 (exposure) |  |
|  |  |
| /lnalpha -17.56787 .4710336 -18.49108 | -16.64466 |
|  |  |
| alpha 2.35e-08 1.11e-08 9.32e-09 | 5.91e-08 |
|  |  |
| Note: Estimates are transformed only in the first equation. |  |
| Note: _cons estimates baseline incidence rate. |  |
| . |  |

**Table S29.- Leukemia model for men (full specification)**

| Negative binomial regression Number of obs = | 1,092 |
| --- | --- |
| Wald chi2(49) = | 1114.91 |
| Dispersion = mean Prob > chi2 = | 0.0000 |
| Log pseudolikelihood = -1386.0349 Pseudo R2 = | 0.2112 |
|  |  |
| Robust |  |
| _freq IRR Std. Err. z P>z [95% Conf. | Interval] |
|  |  |
| t2 1.001126 .0007184 1.57 0.117 .9997192 | 1.002535 |
| 1.level .3827533 .0823744 -4.46 0.000 .2510309 | .5835938 |
| slope 1.034323 .0119989 2.91 0.004 1.011071 | 1.05811 |
| age_group |  |
| 2 1.106359 .1616702 0.69 0.489 .8308287 | 1.473264 |
| 3 1.692826 .2260255 3.94 0.000 1.303048 | 2.199199 |
| 4 2.410365 .2825721 7.50 0.000 1.915555 | 3.03299 |
| 5 5.036026 .5669403 14.36 0.000 4.038893 | 6.279335 |
| 6 9.318343 1.038419 20.03 0.000 7.49001 | 11.59298 |
| 7 12.02969 1.463604 20.44 0.000 9.477466 | 15.26922 |
| week |  |
| 2 1.860634 .4441641 2.60 0.009 1.165374 | 2.970687 |
| 3 1.585788 .3636183 2.01 0.044 1.011734 | 2.485559 |
| 4 1.764175 .411484 2.43 0.015 1.116873 | 2.786633 |
| 5 2.03231 .4613345 3.12 0.002 1.302469 | 3.171121 |
| 6 1.397782 .2969658 1.58 0.115 .9217171 | 2.119735 |
| 7 1.620618 .3489916 2.24 0.025 1.062622 | 2.471624 |
| 8 1.573065 .4108636 1.73 0.083 .9428097 | 2.624636 |
| 9 1.121809 .3206692 0.40 0.688 .6406236 | 1.964423 |
| 10 1.747207 .4139808 2.36 0.019 1.098151 | 2.779886 |
| 11 1.565484 .3410251 2.06 0.040 1.021464 | 2.399244 |
| 12 1.637829 .4014372 2.01 0.044 1.013063 | 2.647896 |
| 13 1.410467 .3499355 1.39 0.166 .867323 | 2.293746 |
| 14 2.155917 .4651461 3.56 0.000 1.41248 | 3.290648 |
| 15 2.034589 .499265 2.89 0.004 1.257771 | 3.291182 |
| 16 1.22315 .289844 0.85 0.395 .7687312 | 1.946188 |
| 17 2.218172 .5293257 3.34 0.001 1.389542 | 3.540941 |
| 18 1.573241 .3711651 1.92 0.055 .9907788 | 2.498124 |
| 19 1.874294 .435955 2.70 0.007 1.188095 | 2.956818 |
| 20 1.292016 .3214046 1.03 0.303 .7934531 | 2.103849 |
| 21 1.590052 .3975609 1.85 0.064 .9740594 | 2.595598 |
| 22 1.068856 .2768557 0.26 0.797 .6433403 | 1.775816 |
| 23 1.312956 .3248659 1.10 0.271 .8084188 | 2.132375 |
| 24 1.753261 .4353725 2.26 0.024 1.077643 | 2.852451 |
| 25 1.341428 .317042 1.24 0.214 .8440899 | 2.131797 |
| 26 1.231659 .3168753 0.81 0.418 .7438707 | 2.039311 |
| 27 1.074583 .3179756 0.24 0.808 .6016784 | 1.91918 |
| 28 1.258858 .3222574 0.90 0.369 .7622124 | 2.07911 |
| 29 1.343582 .3519711 1.13 0.260 .8040433 | 2.245169 |
| 30 1.806791 .4663807 2.29 0.022 1.089407 | 2.996578 |
| 31 1.177597 .3487844 0.55 0.581 .6589993 | 2.104303 |
| 32 1.119727 .2966598 0.43 0.670 .6661856 | 1.88204 |
| 33 1.247441 .3073301 0.90 0.369 .7696808 | 2.021758 |
| 34 1.555246 .3985417 1.72 0.085 .9411812 | 2.569951 |
| 35 1.267798 .3355137 0.90 0.370 .7547198 | 2.129681 |
| 36 1.389094 .3721686 1.23 0.220 .8216298 | 2.348481 |
| 37 1.241395 .322464 0.83 0.405 .7461096 | 2.065464 |
| 38 .6139958 .2074428 -1.44 0.149 .3166511 | 1.190556 |
| 39 1.518072 .4048828 1.57 0.118 .9000566 | 2.560443 |
| year |  |
| 2018 .9053984 .066106 -1.36 0.173 .7846767 | 1.044693 |
| 2019 .9242467 .0786599 -0.93 0.355 .7822483 | 1.092021 |
| 2020 1 (omitted) |  |
| _cons 4.28e-07 9.02e-08 -69.65 0.000 2.84e-07 | 6.47e-07 |
| ln(popula~n) 1 (exposure) |  |
|  |  |
| /lnalpha -16.46575 .1129123 -16.68706 | -16.24445 |
|  |  |
| alpha 7.06e-08 7.98e-09 5.66e-08 | 8.81e-08 |
|  |  |
| Note: Estimates are transformed only in the first equation. |  |
| Note: _cons estimates baseline incidence rate. |  |

**Table S29.- Leukemia model for women (full specification)**

| Negative binomial regression Number of obs = | 1,092 |
| --- | --- |
| Wald chi2(49) = | 1061.06 |
| Dispersion = mean Prob > chi2 = | 0.0000 |
| Log pseudolikelihood = -1423.011 Pseudo R2 = | 0.2123 |
|  |  |
| Robust |  |
| _freq IRR Std. Err. z P>z [95% Conf. | Interval] |
|  |  |
| t2 1.000542 .0007058 0.77 0.443 .9991595 | 1.001926 |
| 1.level .3924774 .0829464 -4.43 0.000 .2593709 | .5938928 |
| slope 1.027885 .0110852 2.55 0.011 1.006387 | 1.049843 |
| age_group |  |
| 2 1.19071 .1739256 1.19 0.232 .8942766 | 1.585404 |
| 3 1.909159 .2539463 4.86 0.000 1.471024 | 2.47779 |
| 4 4.074633 .4862545 11.77 0.000 3.224843 | 5.148353 |
| 5 6.029292 .7008878 15.46 0.000 4.800832 | 7.572094 |
| 6 9.22555 1.090596 18.80 0.000 7.317584 | 11.63099 |
| 7 11.19618 1.357796 19.92 0.000 8.827586 | 14.20031 |
| week |  |
| 2 2.097807 .5615195 2.77 0.006 1.241436 | 3.54492 |
| 3 2.993736 .8339312 3.94 0.000 1.734219 | 5.168007 |
| 4 1.545149 .454016 1.48 0.139 .8686787 | 2.748411 |
| 5 2.240618 .6496818 2.78 0.005 1.269277 | 3.955299 |
| 6 1.691146 .468295 1.90 0.058 .982822 | 2.909961 |
| 7 2.235945 .6075384 2.96 0.003 1.312736 | 3.80842 |
| 8 1.588347 .4579612 1.60 0.109 .9026559 | 2.794915 |
| 9 2.181701 .6284166 2.71 0.007 1.240553 | 3.836852 |
| 10 1.733635 .4910466 1.94 0.052 .9950785 | 3.020353 |
| 11 1.830789 .5292334 2.09 0.036 1.038911 | 3.226253 |
| 12 2.547768 .6978258 3.41 0.001 1.489425 | 4.358142 |
| 13 1.769307 .5543947 1.82 0.069 .9573875 | 3.269782 |
| 14 2.289285 .6661085 2.85 0.004 1.294278 | 4.049228 |
| 15 2.161485 .6183056 2.69 0.007 1.233847 | 3.786548 |
| 16 1.685875 .5177 1.70 0.089 .9235013 | 3.077606 |
| 17 2.140259 .6557329 2.48 0.013 1.174019 | 3.901733 |
| 18 2.014326 .5821816 2.42 0.015 1.143181 | 3.549315 |
| 19 2.003944 .5619033 2.48 0.013 1.15667 | 3.471853 |
| 20 1.936469 .5580106 2.29 0.022 1.100853 | 3.406369 |
| 21 1.586212 .506441 1.44 0.148 .8483832 | 2.965721 |
| 22 1.915623 .5267462 2.36 0.018 1.117511 | 3.283735 |
| 23 1.792935 .494664 2.12 0.034 1.04405 | 3.078987 |
| 24 1.337103 .4134529 0.94 0.347 .7293903 | 2.451148 |
| 25 1.495581 .4638276 1.30 0.194 .8143762 | 2.746596 |
| 26 1.652012 .4869208 1.70 0.089 .9271003 | 2.943743 |
| 27 1.313715 .4033678 0.89 0.374 .7196898 | 2.398044 |
| 28 2.121801 .6456805 2.47 0.013 1.168631 | 3.852404 |
| 29 1.946449 .5564148 2.33 0.020 1.11152 | 3.408543 |
| 30 1.772984 .5351377 1.90 0.058 .9812678 | 3.20348 |
| 31 1.814968 .5392571 2.01 0.045 1.013825 | 3.24919 |
| 32 1.643991 .4839479 1.69 0.091 .9232683 | 2.927325 |
| 33 2.159813 .6067811 2.74 0.006 1.245313 | 3.745881 |
| 34 1.621942 .4810388 1.63 0.103 .9069517 | 2.90059 |
| 35 1.922479 .5241441 2.40 0.017 1.126653 | 3.280446 |
| 36 1.496199 .4658402 1.29 0.196 .8127713 | 2.754293 |
| 37 1.690365 .4876988 1.82 0.069 .9602728 | 2.975545 |
| 38 .8135883 .2809116 -0.60 0.550 .4135292 | 1.600675 |
| 39 1.716024 .4690804 1.98 0.048 1.004257 | 2.932257 |
| year |  |
| 2018 .9027551 .0655711 -1.41 0.159 .7829669 | 1.04087 |
| 2019 1.06185 .0855599 0.74 0.456 .9067264 | 1.243511 |
| 2020 1 (omitted) |  |
| _cons 2.71e-07 7.38e-08 -55.45 0.000 1.59e-07 | 4.62e-07 |
| ln(popula~n) 1 (exposure) |  |
|  |  |
| /lnalpha -16.46072 .0926652 -16.64234 | -16.2791 |
|  |  |
| alpha 7.10e-08 6.58e-09 5.92e-08 | 8.51e-08 |
|  |  |
| Note: Estimates are transformed only in the first equation. |  |
| Note: _cons estimates baseline incidence rate. |  |
| . |  |

**Table S30.- Cervical cancer (includes dysplasia) model (full specification)**

| Negative binomial regression Number of obs = | 1,092 |
| --- | --- |
| Wald chi2(49) = | 7993.24 |
| Dispersion = mean Prob > chi2 = | 0.0000 |
| Log pseudolikelihood = -3418.4287 Pseudo R2 = | 0.2807 |
|  |  |
| Robust |  |
| _freq IRR Std. Err. z P>z [95% Conf. | Interval] |
|  |  |
| t2 1.00192 .0001815 10.59 0.000 1.001564 | 1.002276 |
| 1.level .3353555 .0267327 -13.71 0.000 .2868484 | .3920653 |
| slope 1.00706 .0046641 1.52 0.129 .9979596 | 1.016243 |
| age_group |  |
| 2 1.370266 .0258649 16.69 0.000 1.320498 | 1.421909 |
| 3 1.088022 .0208916 4.39 0.000 1.047836 | 1.129749 |
| 4 .6767461 .0149017 -17.73 0.000 .6481605 | .7065924 |
| 5 .4038538 .0102913 -35.58 0.000 .3841786 | .4245366 |
| 6 .1558999 .0064956 -44.61 0.000 .1436748 | .1691652 |
| 7 .1094872 .0068489 -35.36 0.000 .0968539 | .1237684 |
| week |  |
| 2 1.536282 .1115223 5.91 0.000 1.33254 | 1.771176 |
| 3 1.478054 .1121218 5.15 0.000 1.273855 | 1.714986 |
| 4 1.479935 .1101389 5.27 0.000 1.279072 | 1.712342 |
| 5 1.563451 .1081035 6.46 0.000 1.365302 | 1.790359 |
| 6 1.53965 .1051055 6.32 0.000 1.346833 | 1.76007 |
| 7 1.543632 .1154151 5.81 0.000 1.333216 | 1.787256 |
| 8 1.549812 .1133848 5.99 0.000 1.34278 | 1.788765 |
| 9 1.551327 .1084431 6.28 0.000 1.3527 | 1.77912 |
| 10 1.693229 .1184704 7.53 0.000 1.476249 | 1.942101 |
| 11 1.776689 .1178288 8.67 0.000 1.560128 | 2.02331 |
| 12 1.991458 .1941495 7.07 0.000 1.645078 | 2.41077 |
| 13 1.686512 .1522109 5.79 0.000 1.41308 | 2.012853 |
| 14 1.763818 .1245548 8.04 0.000 1.535836 | 2.025642 |
| 15 1.736242 .1306697 7.33 0.000 1.498127 | 2.012203 |
| 16 1.693329 .1245178 7.16 0.000 1.46605 | 1.955842 |
| 17 1.670564 .1185442 7.23 0.000 1.453655 | 1.91984 |
| 18 1.285041 .0942977 3.42 0.001 1.112897 | 1.483813 |
| 19 1.622491 .1239038 6.34 0.000 1.396945 | 1.884454 |
| 20 1.701264 .119547 7.56 0.000 1.482375 | 1.952474 |
| 21 1.376158 .1079869 4.07 0.000 1.17998 | 1.604951 |
| 22 1.70797 .1243492 7.35 0.000 1.480841 | 1.969936 |
| 23 1.703708 .1386788 6.55 0.000 1.452476 | 1.998396 |
| 24 1.618318 .118735 6.56 0.000 1.40156 | 1.868599 |
| 25 1.5939 .1119409 6.64 0.000 1.388931 | 1.829118 |
| 26 1.445597 .1142079 4.66 0.000 1.238223 | 1.687701 |
| 27 1.483142 .1143187 5.11 0.000 1.275185 | 1.725013 |
| 28 1.674738 .1254349 6.88 0.000 1.446083 | 1.939547 |
| 29 1.245665 .0959919 2.85 0.004 1.071043 | 1.448756 |
| 30 1.503255 .1055055 5.81 0.000 1.310061 | 1.72494 |
| 31 1.556314 .1083292 6.35 0.000 1.357839 | 1.7838 |
| 32 1.61369 .1124603 6.87 0.000 1.407663 | 1.849871 |
| 33 1.402057 .0992369 4.77 0.000 1.220444 | 1.610695 |
| 34 1.605911 .1108586 6.86 0.000 1.402689 | 1.838574 |
| 35 1.663084 .1216401 6.95 0.000 1.440974 | 1.919429 |
| 36 1.760761 .121433 8.20 0.000 1.538142 | 2.015601 |
| 37 1.62322 .1248062 6.30 0.000 1.396144 | 1.887229 |
| 38 .8358989 .1100671 -1.36 0.173 .6457608 | 1.082021 |
| 39 1.60051 .1227144 6.13 0.000 1.377193 | 1.860037 |
| year |  |
| 2018 .966191 .0157939 -2.10 0.035 .9357261 | .9976477 |
| 2019 .9092355 .0178391 -4.85 0.000 .8749353 | .9448804 |
| 2020 1 (omitted) |  |
| _cons .0000403 2.62e-06 -155.72 0.000 .0000355 | .0000458 |
| ln(popula~n) 1 (exposure) |  |
|  |  |
| /lnalpha -4.256016 .2002135 -4.648427 | -3.863605 |
|  |  |
| alpha .0141787 .0028388 .0095767 | .0209922 |
|  |  |
| Note: Estimates are transformed only in the first equation. |  |
| Note: _cons estimates baseline incidence rate. |  |
| . |  |

**Table S31.- Breast cancer model (full specification)**

| Negative binomial regression Number of obs = | 1,092 |
| --- | --- |
| Wald chi2(49) = | 7215.05 |
| Dispersion = mean Prob > chi2 = | 0.0000 |
| Log pseudolikelihood = -3573.0757 Pseudo R2 = | 0.2374 |
|  |  |
| Robust |  |
| _freq IRR Std. Err. z P>z [95% Conf. | Interval] |
|  |  |
| t2 1.001152 .0001673 6.89 0.000 1.000824 | 1.00148 |
| 1.level .3357729 .0232946 -15.73 0.000 .2930844 | .3846792 |
| slope 1.028142 .0038679 7.38 0.000 1.020589 | 1.035751 |
| age_group |  |
| 2 1.891104 .0575724 20.93 0.000 1.781565 | 2.007379 |
| 3 4.448573 .1230376 53.97 0.000 4.213843 | 4.696378 |
| 4 4.748838 .1312955 56.35 0.000 4.498352 | 5.013273 |
| 5 4.77386 .1368324 54.54 0.000 4.513067 | 5.049723 |
| 6 4.214123 .1272008 47.66 0.000 3.972045 | 4.470954 |
| 7 2.927316 .1086335 28.94 0.000 2.721957 | 3.148168 |
| week |  |
| 2 1.859787 .1456818 7.92 0.000 1.595094 | 2.168403 |
| 3 1.663271 .1286427 6.58 0.000 1.429317 | 1.93552 |
| 4 1.75871 .140939 7.05 0.000 1.503076 | 2.057821 |
| 5 1.744956 .1348063 7.21 0.000 1.499771 | 2.030224 |
| 6 1.603141 .1370851 5.52 0.000 1.355767 | 1.89565 |
| 7 1.504742 .1236718 4.97 0.000 1.280865 | 1.767749 |
| 8 1.550969 .1255019 5.42 0.000 1.323504 | 1.817528 |
| 9 1.514853 .1207321 5.21 0.000 1.295778 | 1.770966 |
| 10 1.684004 .1370831 6.40 0.000 1.435664 | 1.975302 |
| 11 1.634038 .1292422 6.21 0.000 1.399386 | 1.908038 |
| 12 1.958806 .1903053 6.92 0.000 1.619176 | 2.369675 |
| 13 1.614682 .1311573 5.90 0.000 1.377037 | 1.893338 |
| 14 1.685575 .1380004 6.38 0.000 1.435684 | 1.97896 |
| 15 1.554207 .1288482 5.32 0.000 1.321119 | 1.82842 |
| 16 1.512922 .124924 5.01 0.000 1.286861 | 1.778695 |
| 17 1.581088 .1273113 5.69 0.000 1.350256 | 1.851382 |
| 18 1.386449 .1106551 4.09 0.000 1.185681 | 1.621213 |
| 19 1.755861 .1468956 6.73 0.000 1.490317 | 2.068721 |
| 20 1.7143 .1366729 6.76 0.000 1.466306 | 2.004237 |
| 21 1.337774 .1114928 3.49 0.000 1.136167 | 1.575157 |
| 22 1.535882 .1193724 5.52 0.000 1.318866 | 1.788609 |
| 23 1.64196 .1346074 6.05 0.000 1.398239 | 1.928163 |
| 24 1.679181 .1363288 6.38 0.000 1.432157 | 1.968814 |
| 25 1.646714 .136121 6.03 0.000 1.400413 | 1.936335 |
| 26 1.509256 .1260972 4.93 0.000 1.281285 | 1.777789 |
| 27 1.475696 .1308366 4.39 0.000 1.240305 | 1.75576 |
| 28 1.507763 .128381 4.82 0.000 1.276016 | 1.781599 |
| 29 1.359677 .1199135 3.48 0.000 1.143842 | 1.616238 |
| 30 1.671466 .1347064 6.37 0.000 1.427242 | 1.95748 |
| 31 1.68875 .1379872 6.41 0.000 1.438845 | 1.98206 |
| 32 1.732919 .135645 7.02 0.000 1.486449 | 2.020256 |
| 33 1.488108 .1210553 4.89 0.000 1.268793 | 1.745334 |
| 34 1.840744 .1577907 7.12 0.000 1.556064 | 2.177506 |
| 35 1.786788 .142026 7.30 0.000 1.529022 | 2.088008 |
| 36 1.868195 .1451341 8.04 0.000 1.604335 | 2.175451 |
| 37 1.90082 .1579105 7.73 0.000 1.615205 | 2.236941 |
| 38 .743619 .0927966 -2.37 0.018 .5822757 | .9496692 |
| 39 1.733029 .139727 6.82 0.000 1.479711 | 2.029713 |
| year |  |
| 2018 .9860384 .0155905 -0.89 0.374 .9559503 | 1.017074 |
| 2019 .9788648 .0184338 -1.13 0.257 .9433939 | 1.015669 |
| 2020 1 (omitted) |  |
| _cons 9.11e-06 6.99e-07 -151.26 0.000 7.83e-06 | .0000106 |
| ln(popula~n) 1 (exposure) |  |
|  |  |
| /lnalpha -4.741559 .2632517 -5.257522 | -4.225595 |
|  |  |
| alpha .008725 .0022969 .0052082 | .0146166 |
|  |  |
| Note: Estimates are transformed only in the first equation. |  |
| Note: _cons estimates baseline incidence rate. |  |
| . |  |

**Table S32.- Testicular cancer model (full specification)**

| Negative binomial regression Number of obs = | 1,092 |
| --- | --- |
| Wald chi2(49) = | 258.54 |
| Dispersion = mean Prob > chi2 = | 0.0000 |
| Log pseudolikelihood = -1824.8486 Pseudo R2 = | 0.0519 |
|  |  |
| Robust |  |
| _freq IRR Std. Err. z P>z [95% Conf. | Interval] |
|  |  |
| t2 1.001096 .0006193 1.77 0.077 .9998827 | 1.00231 |
| 1.level .4693024 .0775294 -4.58 0.000 .3394949 | .6487425 |
| slope 1.012641 .0081743 1.56 0.120 .9967457 | 1.02879 |
| age_group |  |
| 2 1.036118 .0602455 0.61 0.542 .9245192 | 1.161189 |
| 3 .7522589 .0436326 -4.91 0.000 .6714224 | .8428278 |
| 4 .6064054 .0420941 -7.21 0.000 .5292688 | .6947841 |
| 5 .7192675 .054161 -4.38 0.000 .6205758 | .8336545 |
| 6 .7576934 .0611058 -3.44 0.001 .6469141 | .887443 |
| 7 .6740343 .0860455 -3.09 0.002 .5248313 | .8656537 |
| week |  |
| 2 1.404797 .2227896 2.14 0.032 1.029484 | 1.916935 |
| 3 1.347945 .1973498 2.04 0.041 1.011694 | 1.795952 |
| 4 1.568952 .2334459 3.03 0.002 1.172083 | 2.1002 |
| 5 1.095411 .2042791 0.49 0.625 .7600462 | 1.578755 |
| 6 1.341997 .1927272 2.05 0.041 1.012765 | 1.778257 |
| 7 1.173313 .1835675 1.02 0.307 .8634582 | 1.59436 |
| 8 1.053381 .1811333 0.30 0.762 .7520002 | 1.475548 |
| 9 1.053892 .1824641 0.30 0.762 .7506274 | 1.479679 |
| 10 .9831111 .186988 -0.09 0.929 .6771796 | 1.427254 |
| 11 1.018166 .1774979 0.10 0.918 .7234821 | 1.432879 |
| 12 1.319808 .2375499 1.54 0.123 .92748 | 1.878093 |
| 13 1.038423 .1899367 0.21 0.837 .7255748 | 1.486164 |
| 14 1.403688 .2727095 1.75 0.081 .9591767 | 2.0542 |
| 15 1.051729 .1646672 0.32 0.747 .7738072 | 1.429469 |
| 16 1.087731 .1800555 0.51 0.611 .7863568 | 1.504609 |
| 17 1.182673 .2174616 0.91 0.362 .8248055 | 1.695812 |
| 18 .9443263 .1576022 -0.34 0.731 .6808662 | 1.309732 |
| 19 1.188742 .2091901 0.98 0.326 .8419698 | 1.678335 |
| 20 1.263429 .2173417 1.36 0.174 .901826 | 1.770024 |
| 21 1.161972 .2149114 0.81 0.417 .8086538 | 1.669663 |
| 22 1.272829 .2042694 1.50 0.133 .9293202 | 1.743311 |
| 23 1.322789 .2046921 1.81 0.071 .9767273 | 1.791464 |
| 24 1.244377 .195338 1.39 0.164 .9148147 | 1.692663 |
| 25 1.556766 .3132866 2.20 0.028 1.049363 | 2.309515 |
| 26 1.27297 .2027731 1.52 0.130 .931599 | 1.739431 |
| 27 .9682382 .1663014 -0.19 0.851 .6914847 | 1.355757 |
| 28 1.316976 .206113 1.76 0.079 .9690823 | 1.78976 |
| 29 1.033354 .196646 0.17 0.863 .71165 | 1.500485 |
| 30 1.198001 .1735249 1.25 0.212 .9019128 | 1.591291 |
| 31 1.2485 .2218122 1.25 0.212 .8813763 | 1.768542 |
| 32 1.408692 .2125544 2.27 0.023 1.048045 | 1.893441 |
| 33 .8914834 .1389748 -0.74 0.461 .6567772 | 1.210064 |
| 34 1.177953 .2095917 0.92 0.357 .8311412 | 1.66948 |
| 35 1.281021 .2043465 1.55 0.121 .9370743 | 1.751212 |
| 36 1.314288 .1895036 1.90 0.058 .9907359 | 1.743504 |
| 37 1.268185 .2245901 1.34 0.180 .8962689 | 1.79443 |
| 38 .8561818 .163468 -0.81 0.416 .5889099 | 1.244753 |
| 39 1.383474 .2051884 2.19 0.029 1.034489 | 1.850188 |
| year |  |
| 2018 .8846952 .0487902 -2.22 0.026 .7940551 | .9856817 |
| 2019 .966606 .0671502 -0.49 0.625 .843561 | 1.107599 |
| 2020 1 (omitted) |  |
| _cons 4.06e-06 5.01e-07 -100.59 0.000 3.19e-06 | 5.17e-06 |
| ln(popula~n) 1 (exposure) |  |
|  |  |
| /lnalpha -4.69345 1.912147 -8.441189 | -.9457113 |
|  |  |
| alpha .009155 .0175058 .0002158 | .3884032 |
|  |  |
| Note: Estimates are transformed only in the first equation. |  |
| Note: _cons estimates baseline incidence rate. |  |
| . |  |

**Table S33.- Cardiovascular diseases (men, 20 to 29 years) (full specification)**

| Negative binomial regression Number of obs = | 156 |
| --- | --- |
| Wald chi2(43) = | 748.80 |
| Dispersion = mean Prob > chi2 = | 0.0000 |
| Log pseudolikelihood = -550.45612 Pseudo R2 = | 0.1618 |
|  |  |
| Robust |  |
| _freq IRR Std. Err. z P>z [95% Conf. | Interval] |
|  |  |
| t2 1.001224 .0001869 6.55 0.000 1.000858 | 1.00159 |
| 1.level .5600952 .0292684 -11.09 0.000 .5055701 | .6205008 |
| slope 1.004976 .0024998 2.00 0.046 1.000088 | 1.009887 |
| week |  |
| 2 .8137071 .0575093 -2.92 0.004 .7084494 | .9346034 |
| 3 .9923414 .0649039 -0.12 0.906 .8729481 | 1.128064 |
| 4 .8778817 .074004 -1.55 0.122 .7441853 | 1.035597 |
| 5 .780894 .0668874 -2.89 0.004 .6602106 | .9236377 |
| 6 .9024274 .0605373 -1.53 0.126 .7912456 | 1.029232 |
| 7 .9599699 .0648053 -0.61 0.545 .840998 | 1.095772 |
| 8 .9072854 .0569899 -1.55 0.121 .8021893 | 1.02615 |
| 9 .8376468 .0593526 -2.50 0.012 .7290341 | .9624407 |
| 10 .8048022 .0574244 -3.04 0.002 .699768 | .925602 |
| 11 .8499659 .0574505 -2.41 0.016 .7445046 | .9703659 |
| 12 .8460823 .066043 -2.14 0.032 .7260559 | .9859507 |
| 13 .8330769 .063673 -2.39 0.017 .7171778 | .9677058 |
| 14 .8532551 .0639587 -2.12 0.034 .736672 | .9882882 |
| 15 .8788177 .0683992 -1.66 0.097 .7544822 | 1.023643 |
| 16 1.022483 .0669557 0.34 0.734 .8993246 | 1.162507 |
| 17 .7404025 .0736217 -3.02 0.003 .6092968 | .899719 |
| 18 .9056053 .0783213 -1.15 0.252 .7644037 | 1.07289 |
| 19 .8952787 .0779928 -1.27 0.204 .7547537 | 1.061968 |
| 20 .7652349 .0559569 -3.66 0.000 .6630581 | .883157 |
| 21 .7987964 .0641019 -2.80 0.005 .6825411 | .9348532 |
| 22 .7833725 .0515137 -3.71 0.000 .6886433 | .8911327 |
| 23 .7869902 .0846789 -2.23 0.026 .6373551 | .971756 |
| 24 .7959249 .0635667 -2.86 0.004 .680598 | .9307938 |
| 25 .7779197 .0846458 -2.31 0.021 .6285142 | .9628407 |
| 26 .7653561 .067356 -3.04 0.002 .644099 | .9094409 |
| 27 .8144779 .0662106 -2.52 0.012 .6945177 | .9551581 |
| 28 .8419544 .0613103 -2.36 0.018 .7299699 | .9711185 |
| 29 .7706329 .0913436 -2.20 0.028 .6108775 | .9721673 |
| 30 .806091 .0673717 -2.58 0.010 .684293 | .949568 |
| 31 .7909319 .0837321 -2.22 0.027 .642727 | .973311 |
| 32 .8843711 .0682927 -1.59 0.112 .7601568 | 1.028883 |
| 33 .8373779 .0595852 -2.49 0.013 .728371 | .9626987 |
| 34 .8485776 .0656618 -2.12 0.034 .7291665 | .9875438 |
| 35 .81764 .0661368 -2.49 0.013 .6977673 | .9581062 |
| 36 .8419453 .0600928 -2.41 0.016 .7320326 | .968361 |
| 37 .9001201 .0800011 -1.18 0.236 .7562182 | 1.071405 |
| 38 .8588256 .0623394 -2.10 0.036 .7449361 | .990127 |
| 39 .7526181 .0761475 -2.81 0.005 .6172382 | .917691 |
| year |  |
| 2018 .9778202 .0206859 -1.06 0.289 .9381056 | 1.019216 |
| 2019 .9862829 .0212058 -0.64 0.521 .9455839 | 1.028734 |
| 2020 1 (omitted) |  |
| _cons .0000991 6.30e-06 -144.98 0.000 .0000875 | .0001123 |
| ln(popula~n) 1 (exposure) |  |
|  |  |
| /lnalpha -18.78884 .1110795 -19.00655 | -18.57113 |
|  |  |
| alpha 6.92e-09 7.69e-10 5.57e-09 | 8.60e-09 |
|  |  |
| Note: Estimates are transformed only in the first equation. |  |
| Note: _cons estimates baseline incidence rate. |  |
|  |  |

**Table S34.- Cardiovascular diseases (women, 20 to 29 years) (full specification)**

| Negative binomial regression Number of obs = | 156 |
| --- | --- |
| Wald chi2(43) = | 665.67 |
| Dispersion = mean Prob > chi2 = | 0.0000 |
| Log pseudolikelihood = -540.3084 Pseudo R2 = | 0.1455 |
|  |  |
| Robust |  |
| _freq IRR Std. Err. z P>z [95% Conf. | Interval] |
|  |  |
| t2 1.000989 .0002492 3.97 0.000 1.000501 | 1.001478 |
| 1.level .5803605 .0435878 -7.24 0.000 .5009203 | .6723989 |
| slope 1.008253 .0037283 2.22 0.026 1.000972 | 1.015587 |
| week |  |
| 2 1.12413 .1100829 1.19 0.232 .9278134 | 1.361985 |
| 3 1.274513 .0750475 4.12 0.000 1.135594 | 1.430428 |
| 4 1.121444 .1023555 1.26 0.209 .9377506 | 1.34112 |
| 5 1.165513 .0785614 2.27 0.023 1.021273 | 1.330124 |
| 6 1.043172 .137046 0.32 0.748 .8063617 | 1.349528 |
| 7 1.113652 .0773903 1.55 0.121 .9718458 | 1.276149 |
| 8 1.131173 .0781991 1.78 0.075 .9878359 | 1.295309 |
| 9 1.03567 .0800573 0.45 0.650 .8900685 | 1.205089 |
| 10 1.045717 .0796835 0.59 0.557 .9006428 | 1.214159 |
| 11 1.059495 .0643284 0.95 0.341 .9406268 | 1.193385 |
| 12 .8893941 .0788306 -1.32 0.186 .7475647 | 1.058132 |
| 13 1.098209 .0870884 1.18 0.237 .9401227 | 1.282879 |
| 14 1.057348 .121305 0.49 0.627 .8444292 | 1.323953 |
| 15 1.151127 .1214593 1.33 0.182 .9360739 | 1.415587 |
| 16 1.160584 .0930327 1.86 0.063 .9918458 | 1.358029 |
| 17 1.232648 .0766568 3.36 0.001 1.091199 | 1.392433 |
| 18 1.062608 .0811654 0.80 0.427 .9148624 | 1.234215 |
| 19 1.113729 .074155 1.62 0.106 .9774719 | 1.26898 |
| 20 .9407581 .0697468 -0.82 0.410 .8135247 | 1.08789 |
| 21 .979498 .0842677 -0.24 0.810 .8275103 | 1.159401 |
| 22 .9891573 .066803 -0.16 0.872 .8665212 | 1.12915 |
| 23 1.039847 .0770512 0.53 0.598 .8992833 | 1.202381 |
| 24 1.102532 .0860303 1.25 0.211 .9461768 | 1.284726 |
| 25 1.066757 .1038582 0.66 0.507 .8814414 | 1.291033 |
| 26 1.092298 .0786363 1.23 0.220 .9485537 | 1.257826 |
| 27 1.020134 .0839195 0.24 0.809 .8682298 | 1.198616 |
| 28 .9402524 .0575947 -1.01 0.315 .8338819 | 1.060192 |
| 29 .9982096 .0980109 -0.02 0.985 .8234648 | 1.210036 |
| 30 .9389544 .0641582 -0.92 0.357 .8212633 | 1.073511 |
| 31 1.040753 .0584007 0.71 0.477 .9323596 | 1.161748 |
| 32 1.174007 .0705046 2.67 0.008 1.043643 | 1.320655 |
| 33 1.074745 .0644818 1.20 0.230 .9555112 | 1.208858 |
| 34 1.047743 .0643037 0.76 0.447 .9289953 | 1.181669 |
| 35 .981154 .0676082 -0.28 0.782 .8572028 | 1.123028 |
| 36 1.132773 .0869647 1.62 0.104 .9745291 | 1.316712 |
| 37 .9833506 .0705774 -0.23 0.815 .8543103 | 1.131882 |
| 38 .850531 .1018793 -1.35 0.177 .6725592 | 1.075597 |
| 39 1.005077 .0874285 0.06 0.954 .8475313 | 1.191907 |
| year |  |
| 2018 .945453 .0258445 -2.05 0.040 .8961318 | .9974887 |
| 2019 1.08607 .0277561 3.23 0.001 1.033009 | 1.141856 |
| 2020 1 (omitted) |  |
| _cons .0000569 3.27e-06 -169.85 0.000 .0000508 | .0000637 |
| ln(popula~n) 1 (exposure) |  |
|  |  |
| /lnalpha -22.84021 . . | . |
|  |  |
| alpha 1.20e-10 . . | . |
|  |  |
| Note: Estimates are transformed only in the first equation. |  |
| Note: _cons estimates baseline incidence rate. |  |
| . |  |

**Table S35.- Cardiovascular diseases (men, 30 to 39 years) (full specification)**

| Negative binomial regression Number of obs = | 156 |
| --- | --- |
| Wald chi2(43) = | 466.95 |
| Dispersion = mean Prob > chi2 = | 0.0000 |
| Log pseudolikelihood = -566.50124 Pseudo R2 = | 0.1337 |
|  |  |
| Robust |  |
| _freq IRR Std. Err. z P>z [95% Conf. | Interval] |
|  |  |
| t2 1.000754 .0002342 3.22 0.001 1.000295 | 1.001213 |
| 1.level .6214325 .0342363 -8.64 0.000 .5578263 | .6922913 |
| slope 1.004835 .0028349 1.71 0.087 .9992941 | 1.010407 |
| week |  |
| 2 .9748675 .0921566 -0.27 0.788 .8099897 | 1.173307 |
| 3 .8907387 .0722684 -1.43 0.154 .7597832 | 1.044266 |
| 4 .8446043 .0715832 -1.99 0.046 .7153375 | .9972305 |
| 5 .7774015 .0658428 -2.97 0.003 .6584942 | .9177804 |
| 6 .8656473 .0796967 -1.57 0.117 .7227269 | 1.03683 |
| 7 .8784686 .078238 -1.45 0.146 .7377627 | 1.04601 |
| 8 .9193966 .0756879 -1.02 0.307 .7824004 | 1.08038 |
| 9 .9063373 .0709193 -1.26 0.209 .7774721 | 1.056562 |
| 10 1.003225 .0800249 0.04 0.968 .8580254 | 1.172997 |
| 11 .9153561 .0845935 -0.96 0.339 .7637047 | 1.097121 |
| 12 .922086 .0784121 -0.95 0.340 .7805256 | 1.089321 |
| 13 .8416803 .1012648 -1.43 0.152 .6648702 | 1.06551 |
| 14 .9930112 .0986472 -0.07 0.944 .8173245 | 1.206462 |
| 15 .9465179 .0895247 -0.58 0.561 .7863564 | 1.1393 |
| 16 .9390443 .0811562 -0.73 0.467 .7927233 | 1.112373 |
| 17 .8438582 .0692189 -2.07 0.038 .7185356 | .9910389 |
| 18 .9422011 .0799796 -0.70 0.483 .79779 | 1.112753 |
| 19 .8346137 .0711888 -2.12 0.034 .7061253 | .9864821 |
| 20 .8863729 .0724094 -1.48 0.140 .7552318 | 1.040286 |
| 21 .8586498 .0746585 -1.75 0.080 .7241111 | 1.018186 |
| 22 .7953656 .0663231 -2.75 0.006 .6754416 | .9365821 |
| 23 .8264743 .0793108 -1.99 0.047 .6847714 | .9975003 |
| 24 .7737101 .0703335 -2.82 0.005 .6474413 | .9246047 |
| 25 .943883 .0935111 -0.58 0.560 .7773008 | 1.146165 |
| 26 .7951511 .0696946 -2.62 0.009 .6696413 | .944185 |
| 27 .9165757 .0699271 -1.14 0.254 .7892757 | 1.064408 |
| 28 .8590528 .0918388 -1.42 0.155 .6966592 | 1.059301 |
| 29 .9421217 .0742204 -0.76 0.449 .8073268 | 1.099423 |
| 30 .8648066 .0767413 -1.64 0.102 .7267498 | 1.029089 |
| 31 .9000621 .0951824 -1.00 0.319 .7315721 | 1.107357 |
| 32 1.002285 .0869922 0.03 0.979 .845497 | 1.188147 |
| 33 .9997946 .0909021 -0.00 0.998 .8366019 | 1.194821 |
| 34 .9082168 .076261 -1.15 0.252 .7703994 | 1.070688 |
| 35 .9084227 .0824637 -1.06 0.290 .7603589 | 1.085319 |
| 36 .8569088 .0834331 -1.59 0.113 .708039 | 1.03708 |
| 37 .837574 .0662023 -2.24 0.025 .7173709 | .9779185 |
| 38 1.036385 .0964242 0.38 0.701 .8636273 | 1.243702 |
| 39 .8822641 .0926468 -1.19 0.233 .7181476 | 1.083886 |
| year |  |
| 2018 .9679621 .0237864 -1.33 0.185 .9224465 | 1.015724 |
| 2019 .9845485 .0266121 -0.58 0.565 .9337473 | 1.038114 |
| 2020 1 (omitted) |  |
| _cons .0001331 9.98e-06 -119.08 0.000 .0001149 | .0001542 |
| ln(popula~n) 1 (exposure) |  |
|  |  |
| /lnalpha -17.33034 1.092457 -19.47152 | -15.18916 |
|  |  |
| alpha 2.98e-08 3.25e-08 3.50e-09 | 2.53e-07 |
|  |  |
| Note: Estimates are transformed only in the first equation. |  |
| Note: _cons estimates baseline incidence rate. |  |

**Table S36.- Cardiovascular diseases (women, 30 to 39 years) (full specification)**

| Negative binomial regression Number of obs = | 156 |
| --- | --- |
| Wald chi2(43) = | 688.21 |
| Dispersion = mean Prob > chi2 = | 0.0000 |
| Log pseudolikelihood = -555.35937 Pseudo R2 = | 0.1704 |
|  |  |
| Robust |  |
| _freq IRR Std. Err. z P>z [95% Conf. | Interval] |
|  |  |
| t2 1.000385 .0002241 1.72 0.086 .9999459 | 1.000824 |
| 1.level .5585808 .0383777 -8.48 0.000 .4882066 | .6390994 |
| slope 1.007763 .0035031 2.22 0.026 1.00092 | 1.014652 |
| week |  |
| 2 1.192615 .0827789 2.54 0.011 1.040923 | 1.366412 |
| 3 1.161421 .0828903 2.10 0.036 1.00981 | 1.335795 |
| 4 1.097583 .1069204 0.96 0.339 .9068137 | 1.328485 |
| 5 1.123023 .0852868 1.53 0.127 .9677092 | 1.303263 |
| 6 1.002996 .0706564 0.04 0.966 .8736475 | 1.151496 |
| 7 .9484597 .0786712 -0.64 0.524 .8061483 | 1.115894 |
| 8 1.071678 .0855491 0.87 0.386 .9164638 | 1.25318 |
| 9 1.0438 .0853478 0.52 0.600 .8892368 | 1.225228 |
| 10 1.09573 .0812275 1.23 0.217 .9475524 | 1.26708 |
| 11 1.076738 .0840636 0.95 0.344 .923963 | 1.254774 |
| 12 .9970076 .0774018 -0.04 0.969 .8562807 | 1.160862 |
| 13 1.114652 .1205627 1.00 0.316 .9017203 | 1.377865 |
| 14 1.145139 .0833332 1.86 0.063 .9929221 | 1.320691 |
| 15 1.152214 .0951334 1.72 0.086 .9800606 | 1.354606 |
| 16 1.232102 .1266237 2.03 0.042 1.007322 | 1.507041 |
| 17 1.16951 .0891673 2.05 0.040 1.007176 | 1.358008 |
| 18 1.219257 .087348 2.77 0.006 1.059534 | 1.403058 |
| 19 1.130686 .0888045 1.56 0.118 .9693667 | 1.318851 |
| 20 1.239257 .0931708 2.85 0.004 1.069463 | 1.436008 |
| 21 1.075841 .1073977 0.73 0.464 .8846578 | 1.30834 |
| 22 1.011097 .0986036 0.11 0.910 .8351846 | 1.224061 |
| 23 1.054026 .0880221 0.63 0.529 .8948849 | 1.241468 |
| 24 1.012355 .1271957 0.10 0.922 .7913794 | 1.295032 |
| 25 1.145644 .0842558 1.85 0.064 .9918552 | 1.323277 |
| 26 1.071639 .0845394 0.88 0.380 .9181192 | 1.25083 |
| 27 1.036638 .0830496 0.45 0.653 .8859997 | 1.212888 |
| 28 1.168771 .1252481 1.46 0.146 .9473555 | 1.441937 |
| 29 1.018377 .0750607 0.25 0.805 .8813931 | 1.17665 |
| 30 1.002907 .0869246 0.03 0.973 .8462229 | 1.188602 |
| 31 1.162705 .0822812 2.13 0.033 1.012122 | 1.335693 |
| 32 1.223149 .0873663 2.82 0.005 1.06336 | 1.406949 |
| 33 1.099477 .0829078 1.26 0.209 .9484188 | 1.274596 |
| 34 1.121811 .1002124 1.29 0.198 .9416315 | 1.336467 |
| 35 1.071521 .0797813 0.93 0.354 .9260264 | 1.239875 |
| 36 1.056057 .0819974 0.70 0.482 .9069767 | 1.229643 |
| 37 .9936425 .0831746 -0.08 0.939 .8432937 | 1.170797 |
| 38 .9878181 .0695418 -0.17 0.862 .860504 | 1.133969 |
| 39 1.109825 .090086 1.28 0.199 .9465891 | 1.301211 |
| year |  |
| 2018 1.004675 .0192334 0.24 0.808 .9676768 | 1.043088 |
| 2019 1.052395 .0254967 2.11 0.035 1.00359 | 1.103573 |
| 2020 1 (omitted) |  |
| _cons .0000864 6.30e-06 -128.37 0.000 .0000749 | .0000997 |
| ln(popula~n) 1 (exposure) |  |
|  |  |
| /lnalpha -20.95582 . . | . |
|  |  |
| alpha 7.93e-10 . . | . |
|  |  |
| Note: Estimates are transformed only in the first equation. |  |
| Note: _cons estimates baseline incidence rate. |  |
| . |  |

**Table S37.- Cardiovascular diseases (men, 40 to 49 years) (full specification)**

| Negative binomial regression Number of obs = | 156 |
| --- | --- |
| Wald chi2(43) = | 652.52 |
| Dispersion = mean Prob > chi2 = | 0.0000 |
| Log pseudolikelihood = -591.03079 Pseudo R2 = | 0.1320 |
|  |  |
| Robust |  |
| _freq IRR Std. Err. z P>z [95% Conf. | Interval] |
|  |  |
| t2 1.000864 .000182 4.75 0.000 1.000507 | 1.00122 |
| 1.level .6450226 .0310242 -9.12 0.000 .5869943 | .7087873 |
| slope 1.005915 .0026472 2.24 0.025 1.00074 | 1.011117 |
| week |  |
| 2 1.040093 .0491708 0.83 0.406 .9480499 | 1.141072 |
| 3 .9947538 .0550603 -0.10 0.924 .8924851 | 1.108741 |
| 4 .9068298 .0427432 -2.07 0.038 .8268079 | .9945965 |
| 5 .9635777 .0777857 -0.46 0.646 .8225696 | 1.128758 |
| 6 .9258059 .056067 -1.27 0.203 .8221877 | 1.042483 |
| 7 .945447 .0660615 -0.80 0.422 .8244435 | 1.08421 |
| 8 .9816964 .058908 -0.31 0.758 .8727698 | 1.104218 |
| 9 .9181324 .0526066 -1.49 0.136 .820604 | 1.027252 |
| 10 .9026606 .0466439 -1.98 0.047 .8157173 | .9988707 |
| 11 .9370131 .0489447 -1.25 0.213 .8458304 | 1.038026 |
| 12 .8502419 .0394654 -3.50 0.000 .7763054 | .9312203 |
| 13 .8669173 .0614639 -2.01 0.044 .7544457 | .9961559 |
| 14 .9747101 .0826225 -0.30 0.763 .8255101 | 1.150876 |
| 15 .9971395 .0787707 -0.04 0.971 .8541102 | 1.164121 |
| 16 .9770813 .0652406 -0.35 0.728 .8572257 | 1.113695 |
| 17 1.015523 .048018 0.33 0.745 .9256386 | 1.114135 |
| 18 .9814102 .0586629 -0.31 0.754 .8729126 | 1.103393 |
| 19 .9534407 .0496881 -0.91 0.360 .8608624 | 1.055975 |
| 20 .9776571 .0542988 -0.41 0.684 .8768212 | 1.090089 |
| 21 .9957732 .0494949 -0.09 0.932 .9033404 | 1.097664 |
| 22 .8920733 .0416026 -2.45 0.014 .8141493 | .9774557 |
| 23 .8564885 .0551667 -2.41 0.016 .7549103 | .9717347 |
| 24 .9025392 .0665804 -1.39 0.165 .7810393 | 1.04294 |
| 25 1.001974 .0667287 0.03 0.976 .8793637 | 1.141679 |
| 26 .9742773 .0706144 -0.36 0.719 .8452565 | 1.122992 |
| 27 1.002018 .0593671 0.03 0.973 .892162 | 1.1254 |
| 28 1.05331 .0511713 1.07 0.285 .9576428 | 1.158534 |
| 29 .9921805 .0541411 -0.14 0.886 .8915435 | 1.104177 |
| 30 1.005941 .0547553 0.11 0.913 .9041494 | 1.119194 |
| 31 1.039247 .0516128 0.78 0.438 .9428549 | 1.145493 |
| 32 1.045014 .0533327 0.86 0.388 .9455417 | 1.154951 |
| 33 1.042935 .0482861 0.91 0.364 .9524628 | 1.142001 |
| 34 1.036956 .047234 0.80 0.426 .9483911 | 1.133791 |
| 35 1.009592 .074577 0.13 0.897 .8735125 | 1.166871 |
| 36 .9454436 .0492698 -1.08 0.282 .8536445 | 1.047115 |
| 37 .9706656 .0856998 -0.34 0.736 .8164269 | 1.154043 |
| 38 1.086653 .1044074 0.86 0.387 .9001316 | 1.311824 |
| 39 .9300834 .0599929 -1.12 0.261 .8196286 | 1.055423 |
| year |  |
| 2018 .9943226 .0188142 -0.30 0.763 .9581229 | 1.03189 |
| 2019 1.020678 .0233194 0.90 0.370 .9759815 | 1.067422 |
| 2020 1 (omitted) |  |
| _cons .0001686 7.48e-06 -195.76 0.000 .0001545 | .0001839 |
| ln(popula~n) 1 (exposure) |  |
|  |  |
| /lnalpha -26.1966 . . | . |
|  |  |
| alpha 4.20e-12 . . | . |
|  |  |
| Note: Estimates are transformed only in the first equation. |  |
| Note: _cons estimates baseline incidence rate. |  |
|  |  |

**Table S38.- Cardiovascular diseases (men, 40 to 49 years) (full specification)**

| Negative binomial regression Number of obs = | 156 |
| --- | --- |
| Wald chi2(43) = | 743.41 |
| Dispersion = mean Prob > chi2 = | 0.0000 |
| Log pseudolikelihood = -589.1275 Pseudo R2 = | 0.1731 |
|  |  |
| Robust |  |
| _freq IRR Std. Err. z P>z [95% Conf. | Interval] |
|  |  |
| t2 1.000472 .0001816 2.60 0.009 1.000116 | 1.000828 |
| 1.level .5282103 .0284578 -11.85 0.000 .475278 | .5870377 |
| slope 1.013303 .0027675 4.84 0.000 1.007893 | 1.018742 |
| week |  |
| 2 1.288587 .1301607 2.51 0.012 1.057142 | 1.570702 |
| 3 1.303611 .1000449 3.45 0.001 1.121561 | 1.51521 |
| 4 1.267128 .10063 2.98 0.003 1.08448 | 1.480537 |
| 5 1.221732 .0918809 2.66 0.008 1.054292 | 1.415764 |
| 6 1.129416 .0848471 1.62 0.105 .9747819 | 1.30858 |
| 7 1.202609 .094505 2.35 0.019 1.030942 | 1.402861 |
| 8 1.18189 .094906 2.08 0.037 1.009777 | 1.383339 |
| 9 1.170124 .0993285 1.85 0.064 .9907766 | 1.381936 |
| 10 1.296749 .1194224 2.82 0.005 1.082594 | 1.553268 |
| 11 1.204629 .102575 2.19 0.029 1.019466 | 1.423422 |
| 12 1.253418 .1052543 2.69 0.007 1.063206 | 1.47766 |
| 13 1.24251 .1079282 2.50 0.012 1.048002 | 1.473119 |
| 14 1.277227 .1025389 3.05 0.002 1.091268 | 1.494874 |
| 15 1.385033 .122513 3.68 0.000 1.164574 | 1.647225 |
| 16 1.288027 .1018486 3.20 0.001 1.103107 | 1.503946 |
| 17 1.226708 .0983627 2.55 0.011 1.048307 | 1.435471 |
| 18 1.288524 .0961732 3.40 0.001 1.113167 | 1.491505 |
| 19 1.344953 .1003305 3.97 0.000 1.162009 | 1.5567 |
| 20 1.261423 .0965294 3.03 0.002 1.085733 | 1.465542 |
| 21 1.247921 .0981525 2.82 0.005 1.06964 | 1.455916 |
| 22 1.229501 .1025449 2.48 0.013 1.044084 | 1.447845 |
| 23 1.174065 .0902571 2.09 0.037 1.009846 | 1.364988 |
| 24 1.165734 .0855375 2.09 0.037 1.009581 | 1.346039 |
| 25 1.410525 .1206565 4.02 0.000 1.192803 | 1.667987 |
| 26 1.198103 .1088481 1.99 0.047 1.00268 | 1.431615 |
| 27 1.258056 .1025262 2.82 0.005 1.072335 | 1.475941 |
| 28 1.225054 .0944278 2.63 0.008 1.05328 | 1.42484 |
| 29 1.209201 .1152545 1.99 0.046 1.003151 | 1.457573 |
| 30 1.205506 .1041676 2.16 0.031 1.017694 | 1.427978 |
| 31 1.387614 .1109229 4.10 0.000 1.186384 | 1.622975 |
| 32 1.301503 .1035631 3.31 0.001 1.113559 | 1.521167 |
| 33 1.287824 .0936649 3.48 0.001 1.116728 | 1.485133 |
| 34 1.329166 .1126335 3.36 0.001 1.125767 | 1.569315 |
| 35 1.358284 .1067941 3.89 0.000 1.164302 | 1.584586 |
| 36 1.289775 .1002042 3.28 0.001 1.1076 | 1.501913 |
| 37 1.041721 .0810349 0.53 0.599 .8944102 | 1.213293 |
| 38 1.059543 .1030183 0.59 0.552 .8757036 | 1.281977 |
| 39 1.183042 .1032989 1.93 0.054 .9969568 | 1.403861 |
| year |  |
| 2018 .9361129 .0178757 -3.46 0.001 .9017247 | .9718125 |
| 2019 .9985954 .0209009 -0.07 0.946 .9584593 | 1.040412 |
| 2020 1 (omitted) |  |
| _cons .0001242 9.05e-06 -123.39 0.000 .0001077 | .0001433 |
| ln(popula~n) 1 (exposure) |  |
|  |  |
| /lnalpha -18.13596 .4691142 -19.05541 | -17.21651 |
|  |  |
| alpha 1.33e-08 6.24e-09 5.30e-09 | 3.33e-08 |
|  |  |
| Note: Estimates are transformed only in the first equation. |  |
| Note: _cons estimates baseline incidence rate. |  |
| . |  |

**Table S39.- Cardiovascular diseases (men, 50 to 59 years) (full specification)**

| Negative binomial regression Number of obs = | 156 |
| --- | --- |
| Wald chi2(43) = | 576.40 |
| Dispersion = mean Prob > chi2 = | 0.0000 |
| Log pseudolikelihood = -634.30406 Pseudo R2 = | 0.1423 |
|  |  |
| Robust |  |
| _freq IRR Std. Err. z P>z [95% Conf. | Interval] |
|  |  |
| t2 1.000581 .0001946 2.99 0.003 1.0002 | 1.000963 |
| 1.level .6432198 .0265139 -10.71 0.000 .5932973 | .6973429 |
| slope 1.006153 .001966 3.14 0.002 1.002307 | 1.010014 |
| week |  |
| 2 1.043977 .0962394 0.47 0.641 .8714099 | 1.250717 |
| 3 .9934915 .0861355 -0.08 0.940 .8382338 | 1.177506 |
| 4 .9890356 .0949254 -0.11 0.909 .8194369 | 1.193736 |
| 5 .968882 .0857999 -0.36 0.721 .8145022 | 1.152523 |
| 6 .9439322 .0853904 -0.64 0.524 .7905676 | 1.127048 |
| 7 1.02757 .0892723 0.31 0.754 .8666851 | 1.21832 |
| 8 1.049608 .0917713 0.55 0.580 .8843079 | 1.245808 |
| 9 .9752858 .0863009 -0.28 0.777 .8199945 | 1.159986 |
| 10 .9504116 .0859763 -0.56 0.574 .7959947 | 1.134784 |
| 11 .9460134 .0854798 -0.61 0.539 .792473 | 1.129302 |
| 12 .9773623 .0854157 -0.26 0.793 .8235038 | 1.159967 |
| 13 .9700878 .0861701 -0.34 0.732 .8150819 | 1.154572 |
| 14 1.001188 .085833 0.01 0.989 .8463328 | 1.184377 |
| 15 .9846369 .0843399 -0.18 0.857 .8324646 | 1.164626 |
| 16 1.056396 .0902042 0.64 0.521 .8936017 | 1.248849 |
| 17 .9911436 .0945683 -0.09 0.926 .8220924 | 1.194958 |
| 18 1.03503 .0914921 0.39 0.697 .8703833 | 1.230823 |
| 19 1.111454 .0937012 1.25 0.210 .942174 | 1.31115 |
| 20 .9706856 .0831116 -0.35 0.728 .8207244 | 1.148047 |
| 21 .9817207 .0866073 -0.21 0.834 .8258383 | 1.167027 |
| 22 1.008313 .0875993 0.10 0.924 .8504439 | 1.195488 |
| 23 .9400067 .0925115 -0.63 0.530 .7751027 | 1.139994 |
| 24 .9652617 .0986736 -0.35 0.729 .7900076 | 1.179394 |
| 25 1.045998 .0967687 0.49 0.627 .8725363 | 1.253945 |
| 26 1.015426 .095382 0.16 0.871 .8446807 | 1.220687 |
| 27 1.066087 .0962109 0.71 0.478 .8932531 | 1.272363 |
| 28 1.054853 .1008943 0.56 0.577 .8745336 | 1.272353 |
| 29 1.078273 .1033461 0.79 0.432 .893606 | 1.301102 |
| 30 1.023538 .0872369 0.27 0.785 .8660752 | 1.20963 |
| 31 1.090313 .0958395 0.98 0.325 .9177616 | 1.295307 |
| 32 1.007735 .0880281 0.09 0.930 .8491646 | 1.195917 |
| 33 1.078052 .0923781 0.88 0.380 .9113818 | 1.275202 |
| 34 1.139178 .0995251 1.49 0.136 .9598994 | 1.35194 |
| 35 1.044331 .0928661 0.49 0.626 .8772955 | 1.24317 |
| 36 1.024404 .086708 0.28 0.776 .8678082 | 1.209259 |
| 37 .9831266 .0959173 -0.17 0.862 .8120134 | 1.190298 |
| 38 1.04892 .0914775 0.55 0.584 .8841133 | 1.244447 |
| 39 1.051669 .1077325 0.49 0.623 .8603643 | 1.285511 |
| year |  |
| 2018 .9785073 .0155434 -1.37 0.171 .9485122 | 1.009451 |
| 2019 .9984195 .0211097 -0.07 0.940 .9578907 | 1.040663 |
| 2020 1 (omitted) |  |
| _cons .0002574 .000022 -96.58 0.000 .0002177 | .0003045 |
| ln(popula~n) 1 (exposure) |  |
|  |  |
| /lnalpha -18.42072 19.38386 -56.41239 | 19.57095 |
|  |  |
| alpha 1.00e-08 1.94e-07 3.17e-25 | 3.16e+08 |
|  |  |
| Note: Estimates are transformed only in the first equation. |  |
| Note: _cons estimates baseline incidence rate. |  |

**Table S40.- Cardiovascular diseases (women, 50 to 59 years) (full specification)**

| Negative binomial regression Number of obs = | 156 |
| --- | --- |
| Wald chi2(43) = | 1390.80 |
| Dispersion = mean Prob > chi2 = | 0.0000 |
| Log pseudolikelihood = -621.32885 Pseudo R2 = | 0.1960 |
|  |  |
| Robust |  |
| _freq IRR Std. Err. z P>z [95% Conf. | Interval] |
|  |  |
| t2 1.000183 .0001716 1.07 0.286 .9998469 | 1.000519 |
| 1.level .5665758 .0261445 -12.31 0.000 .5175824 | .6202069 |
| slope 1.009862 .0023804 4.16 0.000 1.005207 | 1.014538 |
| week |  |
| 2 1.233155 .1188991 2.17 0.030 1.020812 | 1.489668 |
| 3 1.264066 .1257343 2.36 0.018 1.040165 | 1.536162 |
| 4 1.170339 .1121667 1.64 0.101 .9699097 | 1.412186 |
| 5 1.198097 .1177353 1.84 0.066 .9882018 | 1.452574 |
| 6 1.216372 .1214868 1.96 0.050 1.000118 | 1.479385 |
| 7 1.174785 .1122049 1.69 0.092 .9742253 | 1.416634 |
| 8 1.197774 .1211666 1.78 0.074 .9823531 | 1.460435 |
| 9 1.162534 .112253 1.56 0.119 .9620874 | 1.404742 |
| 10 1.190222 .113574 1.82 0.068 .9871974 | 1.434999 |
| 11 1.164451 .1128076 1.57 0.116 .9630751 | 1.407935 |
| 12 1.162503 .1164625 1.50 0.133 .9552534 | 1.414717 |
| 13 1.186448 .1173663 1.73 0.084 .9773401 | 1.440295 |
| 14 1.226101 .1181512 2.12 0.034 1.015083 | 1.480986 |
| 15 1.239232 .1372105 1.94 0.053 .9974834 | 1.53957 |
| 16 1.273301 .1234778 2.49 0.013 1.052898 | 1.539841 |
| 17 1.218072 .1259965 1.91 0.057 .9945466 | 1.491834 |
| 18 1.300818 .1272274 2.69 0.007 1.073901 | 1.575684 |
| 19 1.294443 .1253727 2.66 0.008 1.070632 | 1.565041 |
| 20 1.414795 .134879 3.64 0.000 1.173666 | 1.705464 |
| 21 1.226281 .1194178 2.09 0.036 1.013207 | 1.484163 |
| 22 1.309907 .1283552 2.75 0.006 1.081018 | 1.58726 |
| 23 1.175885 .1196807 1.59 0.111 .96323 | 1.435487 |
| 24 1.254039 .1382977 2.05 0.040 1.010274 | 1.556622 |
| 25 1.312932 .1247959 2.86 0.004 1.089769 | 1.581795 |
| 26 1.210574 .1278575 1.81 0.070 .984215 | 1.488994 |
| 27 1.260705 .1214418 2.41 0.016 1.043803 | 1.52268 |
| 28 1.218548 .1196218 2.01 0.044 1.005269 | 1.477077 |
| 29 1.244575 .120713 2.26 0.024 1.02911 | 1.505152 |
| 30 1.221264 .1271626 1.92 0.055 .9958168 | 1.497752 |
| 31 1.336798 .1345578 2.88 0.004 1.097454 | 1.628339 |
| 32 1.294759 .1268078 2.64 0.008 1.068619 | 1.568755 |
| 33 1.384249 .1311075 3.43 0.001 1.149724 | 1.666613 |
| 34 1.335525 .1324813 2.92 0.004 1.099549 | 1.622144 |
| 35 1.407669 .1366214 3.52 0.000 1.163823 | 1.702605 |
| 36 1.400862 .1486099 3.18 0.001 1.137878 | 1.724625 |
| 37 1.075561 .1165511 0.67 0.501 .8697531 | 1.330068 |
| 38 1.072883 .1082411 0.70 0.486 .8803922 | 1.30746 |
| 39 1.256028 .1433105 2.00 0.046 1.004335 | 1.570796 |
| year |  |
| 2018 .9968208 .0157114 -0.20 0.840 .9664978 | 1.028095 |
| 2019 1.066782 .0183192 3.76 0.000 1.031474 | 1.103297 |
| 2020 1 (omitted) |  |
| _cons .0001746 .000017 -88.65 0.000 .0001442 | .0002114 |
| ln(popula~n) 1 (exposure) |  |
|  |  |
| /lnalpha -18.33543 .7163263 -19.73941 | -16.93146 |
|  |  |
| alpha 1.09e-08 7.80e-09 2.67e-09 | 4.43e-08 |
|  |  |
| Note: Estimates are transformed only in the first equation. |  |
| Note: _cons estimates baseline incidence rate. |  |
| . |  |

**Table S41.- Cardiovascular diseases (men, 60 to 69 years) (full specification)**

| Negative binomial regression Number of obs = | 156 |
| --- | --- |
| Wald chi2(43) = | 729.52 |
| Dispersion = mean Prob > chi2 = | 0.0000 |
| Log pseudolikelihood = -644.7415 Pseudo R2 = | 0.1527 |
|  |  |
| Robust |  |
| _freq IRR Std. Err. z P>z [95% Conf. | Interval] |
|  |  |
| t2 .9999421 .0001768 -0.33 0.743 .9995957 | 1.000289 |
| 1.level .6535087 .0259149 -10.73 0.000 .60464 | .706327 |
| slope 1.009446 .0016435 5.77 0.000 1.00623 | 1.012672 |
| week |  |
| 2 1.047178 .0774167 0.62 0.533 .9059251 | 1.210456 |
| 3 1.040787 .0869233 0.48 0.632 .8836335 | 1.22589 |
| 4 1.055564 .084213 0.68 0.498 .9027671 | 1.234223 |
| 5 .9601696 .069813 -0.56 0.576 .8326411 | 1.10723 |
| 6 1.007228 .0780271 0.09 0.926 .8653413 | 1.172379 |
| 7 1.044219 .0885596 0.51 0.610 .8843044 | 1.233052 |
| 8 .9967825 .0710319 -0.05 0.964 .8668478 | 1.146194 |
| 9 1.018189 .073987 0.25 0.804 .8830301 | 1.174035 |
| 10 .989691 .0725095 -0.14 0.888 .8573073 | 1.142517 |
| 11 .9833877 .0703584 -0.23 0.815 .8547201 | 1.131425 |
| 12 1.085835 .0753922 1.19 0.236 .9476821 | 1.244127 |
| 13 .9906578 .0680594 -0.14 0.891 .8658548 | 1.13345 |
| 14 1.06083 .0751183 0.83 0.404 .9233607 | 1.218765 |
| 15 1.045975 .07313 0.64 0.520 .9120293 | 1.199592 |
| 16 .9986062 .0768584 -0.02 0.986 .8587782 | 1.161201 |
| 17 1.041693 .0734148 0.58 0.562 .9072988 | 1.195995 |
| 18 1.058133 .088467 0.68 0.499 .8982017 | 1.24654 |
| 19 1.066088 .0792081 0.86 0.389 .9216172 | 1.233206 |
| 20 1.010747 .0769121 0.14 0.888 .8707047 | 1.173313 |
| 21 1.040163 .0720921 0.57 0.570 .9080417 | 1.191507 |
| 22 1.000575 .0973324 0.01 0.995 .8268904 | 1.210742 |
| 23 1.035727 .0727515 0.50 0.617 .9025167 | 1.188599 |
| 24 .9986748 .0767437 -0.02 0.986 .8590393 | 1.161008 |
| 25 .9982909 .0765775 -0.02 0.982 .8589396 | 1.16025 |
| 26 1.009626 .0779161 0.12 0.901 .867902 | 1.174493 |
| 27 1.091135 .0851998 1.12 0.264 .9362967 | 1.271579 |
| 28 1.151144 .0813717 1.99 0.046 1.002213 | 1.322206 |
| 29 1.09437 .0803212 1.23 0.219 .9477426 | 1.263683 |
| 30 1.150511 .0868522 1.86 0.063 .9922785 | 1.333976 |
| 31 1.179675 .094728 2.06 0.040 1.007885 | 1.380747 |
| 32 1.146317 .0831476 1.88 0.060 .9944052 | 1.321437 |
| 33 1.161468 .0854924 2.03 0.042 1.005432 | 1.34172 |
| 34 1.103042 .0830731 1.30 0.193 .9516687 | 1.278493 |
| 35 1.076973 .0784352 1.02 0.309 .933711 | 1.242217 |
| 36 1.132056 .0857743 1.64 0.102 .9758288 | 1.313295 |
| 37 1.0366 .074739 0.50 0.618 .8999937 | 1.193941 |
| 38 1.043779 .0739536 0.60 0.545 .9084462 | 1.199272 |
| 39 1.076915 .1073144 0.74 0.457 .8858482 | 1.309193 |
| year |  |
| 2018 .9805371 .0149213 -1.29 0.196 .9517237 | 1.010223 |
| 2019 1.014081 .0194057 0.73 0.465 .9767507 | 1.052837 |
| 2020 1 (omitted) |  |
| _cons .0004266 .0000293 -112.81 0.000 .0003728 | .0004882 |
| ln(popula~n) 1 (exposure) |  |
|  |  |
| /lnalpha -14.58141 141.1824 -291.2939 | 262.131 |
|  |  |
| alpha 4.65e-07 .0000656 3.1e-127 | 7.0e+113 |
|  |  |
| Note: Estimates are transformed only in the first equation. |  |
| Note: _cons estimates baseline incidence rate. |  |

**Table S42.- Cardiovascular diseases (women, 60 to 69 years) (full specification)**

| Negative binomial regression Number of obs = | 156 |
| --- | --- |
| Wald chi2(43) = | 837.10 |
| Dispersion = mean Prob > chi2 = | 0.0000 |
| Log pseudolikelihood = -634.38804 Pseudo R2 = | 0.1890 |
|  |  |
| Robust |  |
| _freq IRR Std. Err. z P>z [95% Conf. | Interval] |
|  |  |
| t2 .9997867 .0002232 -0.96 0.339 .9993494 | 1.000224 |
| 1.level .5917919 .0304338 -10.20 0.000 .5350504 | .6545507 |
| slope 1.010321 .0020165 5.14 0.000 1.006377 | 1.014281 |
| week |  |
| 2 1.182514 .1011456 1.96 0.050 .9999988 | 1.398342 |
| 3 1.103412 .0804628 1.35 0.177 .9564595 | 1.272943 |
| 4 1.098653 .0824056 1.25 0.210 .9484515 | 1.27264 |
| 5 1.150244 .0816301 1.97 0.049 1.00088 | 1.321897 |
| 6 1.005372 .0811199 0.07 0.947 .8583145 | 1.177626 |
| 7 1.100884 .091601 1.16 0.248 .935224 | 1.295887 |
| 8 1.064585 .0730896 0.91 0.362 .9305519 | 1.217923 |
| 9 1.051636 .0861208 0.61 0.539 .8956924 | 1.234731 |
| 10 1.109959 .0793573 1.46 0.145 .964828 | 1.276922 |
| 11 1.127131 .0803953 1.68 0.093 .9800775 | 1.296249 |
| 12 1.023945 .0722914 0.34 0.737 .8916231 | 1.175905 |
| 13 1.044439 .0785659 0.58 0.563 .9012665 | 1.210356 |
| 14 1.034439 .0744865 0.47 0.638 .8982819 | 1.191234 |
| 15 1.141157 .0761282 1.98 0.048 1.001292 | 1.30056 |
| 16 1.17176 .081339 2.28 0.022 1.022707 | 1.342535 |
| 17 1.119234 .079994 1.58 0.115 .972935 | 1.287532 |
| 18 1.136157 .0873124 1.66 0.097 .9772921 | 1.320846 |
| 19 1.271576 .0931143 3.28 0.001 1.101568 | 1.467823 |
| 20 1.145729 .0812085 1.92 0.055 .9971243 | 1.316481 |
| 21 1.138493 .0814883 1.81 0.070 .989476 | 1.309953 |
| 22 1.094007 .0767848 1.28 0.201 .953404 | 1.255345 |
| 23 1.058636 .0760232 0.79 0.428 .9196438 | 1.218634 |
| 24 1.060495 .0868318 0.72 0.473 .903261 | 1.245098 |
| 25 1.195482 .0988511 2.16 0.031 1.016622 | 1.405809 |
| 26 1.121677 .0861187 1.50 0.135 .9649732 | 1.303828 |
| 27 1.074714 .090658 0.85 0.393 .9109389 | 1.267933 |
| 28 1.1102 .0766778 1.51 0.130 .9696419 | 1.271132 |
| 29 1.196751 .0874693 2.46 0.014 1.037028 | 1.381075 |
| 30 1.072442 .0842651 0.89 0.373 .9193739 | 1.250994 |
| 31 1.177547 .0781236 2.46 0.014 1.033965 | 1.341067 |
| 32 1.158531 .0790596 2.16 0.031 1.013493 | 1.324325 |
| 33 1.185981 .078501 2.58 0.010 1.041684 | 1.350266 |
| 34 1.178556 .0798658 2.42 0.015 1.031972 | 1.345961 |
| 35 1.223066 .0834063 2.95 0.003 1.070046 | 1.397968 |
| 36 1.199705 .0848778 2.57 0.010 1.044366 | 1.378148 |
| 37 1.080316 .0789968 1.06 0.291 .9360684 | 1.246791 |
| 38 1.034509 .0724386 0.48 0.628 .9018434 | 1.186689 |
| 39 1.16159 .0974281 1.79 0.074 .9855046 | 1.369138 |
| year |  |
| 2018 1.049771 .0188819 2.70 0.007 1.013408 | 1.087439 |
| 2019 1.087855 .02551 3.59 0.000 1.038987 | 1.13902 |
| 2020 1 (omitted) |  |
| _cons .0002853 .0000187 -124.40 0.000 .0002508 | .0003244 |
| ln(popula~n) 1 (exposure) |  |
|  |  |
| /lnalpha -17.19359 32.01709 -79.94593 | 45.55875 |
|  |  |
| alpha 3.41e-08 1.09e-06 1.91e-35 | 6.11e+19 |
|  |  |
| Note: Estimates are transformed only in the first equation. |  |
| Note: _cons estimates baseline incidence rate. |  |
| . |  |

**Table S43.- Cardiovascular diseases (men, 70 to 79 years) (full specification)**

| Negative binomial regression Number of obs = | 156 |
| --- | --- |
| Wald chi2(43) = | 5097.13 |
| Dispersion = mean Prob > chi2 = | 0.0000 |
| Log pseudolikelihood = -620.05602 Pseudo R2 = | 0.1982 |
|  |  |
| Robust |  |
| _freq IRR Std. Err. z P>z [95% Conf. | Interval] |
|  |  |
| t2 1.000025 .0002033 0.12 0.901 .999627 | 1.000424 |
| 1.level .5849985 .0245687 -12.77 0.000 .5387733 | .6351896 |
| slope 1.012745 .0016349 7.84 0.000 1.009545 | 1.015954 |
| week |  |
| 2 1.152538 .0883879 1.85 0.064 .9916921 | 1.339472 |
| 3 1.196789 .0906923 2.37 0.018 1.031606 | 1.388422 |
| 4 1.164438 .0893299 1.98 0.047 1.001881 | 1.353369 |
| 5 1.170374 .1006981 1.83 0.067 .9887533 | 1.385356 |
| 6 1.095788 .0866581 1.16 0.247 .9384494 | 1.279505 |
| 7 1.039042 .0815827 0.49 0.626 .8908388 | 1.211901 |
| 8 1.130899 .0926713 1.50 0.133 .9631017 | 1.32793 |
| 9 1.124589 .0892409 1.48 0.139 .9626031 | 1.313833 |
| 10 1.085638 .0864597 1.03 0.302 .9287438 | 1.269038 |
| 11 1.044027 .0841169 0.53 0.593 .8915192 | 1.222624 |
| 12 1.089541 .0968286 0.96 0.335 .9153695 | 1.296853 |
| 13 1.063596 .0768529 0.85 0.394 .9231474 | 1.225413 |
| 14 1.163331 .0845598 2.08 0.037 1.008861 | 1.341452 |
| 15 1.119153 .0782121 1.61 0.107 .9758955 | 1.283441 |
| 16 1.215059 .0881124 2.69 0.007 1.054073 | 1.400631 |
| 17 1.06447 .0797637 0.83 0.404 .9190746 | 1.232868 |
| 18 1.147932 .0834674 1.90 0.058 .9954612 | 1.323756 |
| 19 1.199556 .0987998 2.21 0.027 1.020734 | 1.409706 |
| 20 1.142381 .087818 1.73 0.083 .9826002 | 1.328144 |
| 21 1.132911 .0855923 1.65 0.099 .9769827 | 1.313726 |
| 22 1.23271 .090309 2.86 0.004 1.067829 | 1.423051 |
| 23 1.099321 .081625 1.28 0.202 .9504349 | 1.271529 |
| 24 1.15451 .0842743 1.97 0.049 1.000608 | 1.332085 |
| 25 1.223932 .1084505 2.28 0.023 1.028806 | 1.456065 |
| 26 1.139821 .083356 1.79 0.074 .9876145 | 1.315484 |
| 27 1.107063 .095745 1.18 0.240 .9344489 | 1.311562 |
| 28 1.229426 .0895253 2.84 0.005 1.065906 | 1.418032 |
| 29 1.189291 .0850962 2.42 0.015 1.033672 | 1.368338 |
| 30 1.27453 .0954631 3.24 0.001 1.100511 | 1.476065 |
| 31 1.277435 .1014841 3.08 0.002 1.093242 | 1.492661 |
| 32 1.257411 .0935422 3.08 0.002 1.086811 | 1.45479 |
| 33 1.264534 .0920787 3.22 0.001 1.09635 | 1.458518 |
| 34 1.282913 .0943689 3.39 0.001 1.110668 | 1.48187 |
| 35 1.311018 .0929574 3.82 0.000 1.140918 | 1.506479 |
| 36 1.196535 .0879715 2.44 0.015 1.035961 | 1.381998 |
| 37 1.160045 .0849507 2.03 0.043 1.004942 | 1.339088 |
| 38 1.150357 .0825441 1.95 0.051 .9994347 | 1.324069 |
| 39 1.176951 .10069 1.90 0.057 .9952605 | 1.39181 |
| year |  |
| 2018 1.057341 .0158215 3.73 0.000 1.026781 | 1.088809 |
| 2019 1.038381 .0226419 1.73 0.084 .994939 | 1.08372 |
| 2020 1 (omitted) |  |
| _cons .0005642 .0000402 -104.93 0.000 .0004906 | .0006488 |
| ln(popula~n) 1 (exposure) |  |
|  |  |
| /lnalpha -18.59202 .3298249 -19.23846 | -17.94557 |
|  |  |
| alpha 8.43e-09 2.78e-09 4.41e-09 | 1.61e-08 |
|  |  |
| Note: Estimates are transformed only in the first equation. |  |
| Note: _cons estimates baseline incidence rate. |  |

**Table S44.- Cardiovascular diseases (women, 70 to 79 years) (full specification)**

| Negative binomial regression Number of obs = | 156 |
| --- | --- |
| Wald chi2(43) = | 1493.25 |
| Dispersion = mean Prob > chi2 = | 0.0000 |
| Log pseudolikelihood = -633.92849 Pseudo R2 = | 0.1988 |
|  |  |
| Robust |  |
| _freq IRR Std. Err. z P>z [95% Conf. | Interval] |
|  |  |
| t2 1.000231 .0002178 1.06 0.289 .9998043 | 1.000658 |
| 1.level .5465377 .0252128 -13.10 0.000 .4992897 | .5982567 |
| slope 1.009075 .0018229 5.00 0.000 1.005508 | 1.012654 |
| week |  |
| 2 1.199758 .1385977 1.58 0.115 .9566685 | 1.504615 |
| 3 1.191764 .1252255 1.67 0.095 .9699511 | 1.464303 |
| 4 1.1824 .1212226 1.63 0.102 .9671576 | 1.445546 |
| 5 1.13165 .1154716 1.21 0.225 .9265251 | 1.382189 |
| 6 1.154073 .1222276 1.35 0.176 .937741 | 1.420313 |
| 7 1.209516 .1218788 1.89 0.059 .9927479 | 1.473616 |
| 8 1.113457 .114583 1.04 0.296 .9100776 | 1.362286 |
| 9 1.172966 .1256939 1.49 0.137 .9507609 | 1.447102 |
| 10 1.163662 .1339543 1.32 0.188 .9286266 | 1.458184 |
| 11 1.136538 .1164616 1.25 0.212 .9297384 | 1.389335 |
| 12 1.111049 .1157611 1.01 0.312 .9058278 | 1.362764 |
| 13 1.158127 .121652 1.40 0.162 .9426366 | 1.422879 |
| 14 1.201865 .1194672 1.85 0.064 .9891104 | 1.460381 |
| 15 1.178842 .1242101 1.56 0.118 .9588876 | 1.449251 |
| 16 1.14665 .1212331 1.29 0.196 .9320404 | 1.410674 |
| 17 1.277786 .1336618 2.34 0.019 1.040924 | 1.568547 |
| 18 1.142585 .1212064 1.26 0.209 .9280947 | 1.406646 |
| 19 1.259269 .1314174 2.21 0.027 1.02633 | 1.545076 |
| 20 1.133902 .1178242 1.21 0.227 .9249682 | 1.39003 |
| 21 1.178338 .1233708 1.57 0.117 .9597317 | 1.446738 |
| 22 1.129789 .1194983 1.15 0.249 .9182588 | 1.390046 |
| 23 1.170943 .1175818 1.57 0.116 .9617482 | 1.425642 |
| 24 1.139256 .1301671 1.14 0.254 .9106813 | 1.425202 |
| 25 1.234465 .1271615 2.04 0.041 1.008781 | 1.510638 |
| 26 1.187676 .1276862 1.60 0.110 .9620243 | 1.466257 |
| 27 1.256713 .1252384 2.29 0.022 1.033735 | 1.527788 |
| 28 1.154654 .1175927 1.41 0.158 .9457219 | 1.409744 |
| 29 1.16811 .119068 1.52 0.127 .9565751 | 1.426424 |
| 30 1.290248 .134109 2.45 0.014 1.052444 | 1.581785 |
| 31 1.315136 .1348381 2.67 0.008 1.075719 | 1.607839 |
| 32 1.243521 .1278475 2.12 0.034 1.016578 | 1.521129 |
| 33 1.252093 .1275239 2.21 0.027 1.025517 | 1.528728 |
| 34 1.32694 .1320699 2.84 0.004 1.091771 | 1.612765 |
| 35 1.263142 .1254805 2.35 0.019 1.039666 | 1.534654 |
| 36 1.192323 .1191524 1.76 0.078 .980236 | 1.450297 |
| 37 1.136486 .1254721 1.16 0.247 .915353 | 1.411042 |
| 38 1.10281 .1122072 0.96 0.336 .9034284 | 1.346194 |
| 39 1.251145 .1351532 2.07 0.038 1.012413 | 1.546171 |
| year |  |
| 2018 1.008789 .0162879 0.54 0.588 .9773655 | 1.041224 |
| 2019 1.037 .0234066 1.61 0.107 .9921238 | 1.083906 |
| 2020 1 (omitted) |  |
| _cons .0004277 .0000437 -75.92 0.000 .0003501 | .0005225 |
| ln(popula~n) 1 (exposure) |  |
|  |  |
| /lnalpha -18.61065 4.575221 -27.57792 | -9.64338 |
|  |  |
| alpha 8.27e-09 3.78e-08 1.05e-12 | .0000649 |
|  |  |
| Note: Estimates are transformed only in the first equation. |  |
| Note: _cons estimates baseline incidence rate. |  |
| . |  |

**Table S44.- Cardiovascular diseases (men, 80 years and older) (full specification)**

| Negative binomial regression Number of obs = | 156 |
| --- | --- |
| Wald chi2(43) = | 1155.68 |
| Dispersion = mean Prob > chi2 = | 0.0000 |
| Log pseudolikelihood = -589.31035 Pseudo R2 = | 0.1761 |
|  |  |
| Robust |  |
| _freq IRR Std. Err. z P>z [95% Conf. | Interval] |
|  |  |
| t2 1.000415 .0001904 2.18 0.029 1.000042 | 1.000788 |
| 1.level .6157759 .0346612 -8.61 0.000 .5514545 | .6875998 |
| slope 1.004933 .0028232 1.75 0.080 .999415 | 1.010482 |
| week |  |
| 2 1.114216 .0831576 1.45 0.147 .9625906 | 1.289726 |
| 3 1.070382 .0832489 0.87 0.382 .9190451 | 1.24664 |
| 4 1.102422 .0844473 1.27 0.203 .9487342 | 1.281006 |
| 5 1.02999 .0769306 0.40 0.692 .8897262 | 1.192367 |
| 6 1.078387 .0811475 1.00 0.316 .9305131 | 1.24976 |
| 7 .8936022 .0714182 -1.41 0.159 .7640376 | 1.045138 |
| 8 .9747187 .0903291 -0.28 0.782 .8128245 | 1.168858 |
| 9 .9822078 .068222 -0.26 0.796 .8571973 | 1.125449 |
| 10 .9998925 .0870199 -0.00 0.999 .8430898 | 1.185858 |
| 11 1.033866 .0790065 0.44 0.663 .8900545 | 1.200914 |
| 12 1.023072 .0820602 0.28 0.776 .8742419 | 1.197239 |
| 13 .9830404 .0719353 -0.23 0.815 .8516939 | 1.134643 |
| 14 1.026614 .0867528 0.31 0.756 .8699158 | 1.211537 |
| 15 1.074549 .0861489 0.90 0.370 .9182974 | 1.257386 |
| 16 1.036858 .0808247 0.46 0.642 .8899524 | 1.208013 |
| 17 1.077876 .0751217 1.08 0.282 .9402535 | 1.235642 |
| 18 1.020106 .0901693 0.23 0.822 .8578389 | 1.213067 |
| 19 .9714711 .0777499 -0.36 0.718 .8304347 | 1.13646 |
| 20 1.139767 .1081939 1.38 0.168 .9462686 | 1.372832 |
| 21 .9951376 .0863906 -0.06 0.955 .8394368 | 1.179718 |
| 22 1.096012 .0812674 1.24 0.216 .9477639 | 1.267449 |
| 23 1.056435 .0796976 0.73 0.467 .91123 | 1.224779 |
| 24 1.130228 .1150235 1.20 0.229 .9258464 | 1.379726 |
| 25 1.099581 .0837515 1.25 0.213 .947096 | 1.276617 |
| 26 1.10444 .0830219 1.32 0.186 .9531393 | 1.279758 |
| 27 1.208711 .0880624 2.60 0.009 1.047869 | 1.394241 |
| 28 1.153799 .096668 1.71 0.088 .9790712 | 1.359708 |
| 29 1.226803 .0888996 2.82 0.005 1.064371 | 1.414024 |
| 30 1.341342 .0926396 4.25 0.000 1.171525 | 1.535775 |
| 31 1.214013 .083771 2.81 0.005 1.060443 | 1.389822 |
| 32 1.216401 .0899535 2.65 0.008 1.052277 | 1.406124 |
| 33 1.199084 .083632 2.60 0.009 1.045879 | 1.374732 |
| 34 1.275748 .0896346 3.47 0.001 1.111627 | 1.4641 |
| 35 1.210398 .1064383 2.17 0.030 1.018771 | 1.43807 |
| 36 1.26065 .1048522 2.78 0.005 1.07102 | 1.483856 |
| 37 1.182477 .097906 2.02 0.043 1.005346 | 1.390817 |
| 38 1.013418 .0871754 0.15 0.877 .8561842 | 1.199527 |
| 39 1.161215 .1039793 1.67 0.095 .9743007 | 1.383988 |
| year |  |
| 2018 1.020187 .0180782 1.13 0.259 .985363 | 1.056243 |
| 2019 1.056319 .0242418 2.39 0.017 1.009859 | 1.104917 |
| 2020 1 (omitted) |  |
| _cons .0007939 .0000549 -103.15 0.000 .0006932 | .0009092 |
| ln(popula~n) 1 (exposure) |  |
|  |  |
| /lnalpha -18.67884 .5277661 -19.71324 | -17.64444 |
|  |  |
| alpha 7.72e-09 4.08e-09 2.75e-09 | 2.17e-08 |
|  |  |
| Note: Estimates are transformed only in the first equation. |  |
| Note: _cons estimates baseline incidence rate. |  |
|  |  |

**Table S45.- Cardiovascular diseases (women, 80 years and older) (full specification)**

| Negative binomial regression Number of obs = | 156 |
| --- | --- |
| Wald chi2(43) = | 809.60 |
| Dispersion = mean Prob > chi2 = | 0.0000 |
| Log pseudolikelihood = -631.91103 Pseudo R2 = | 0.1957 |
|  |  |
| Robust |  |
| _freq IRR Std. Err. z P>z [95% Conf. | Interval] |
|  |  |
| t2 1.000291 .0001778 1.64 0.102 .9999425 | 1.00064 |
| 1.level .509888 .0275157 -12.48 0.000 .4587123 | .566773 |
| slope 1.013006 .0028916 4.53 0.000 1.007355 | 1.01869 |
| week |  |
| 2 1.150429 .1079178 1.49 0.135 .9572196 | 1.382637 |
| 3 1.217607 .0913274 2.62 0.009 1.051144 | 1.410432 |
| 4 1.178968 .0886277 2.19 0.029 1.017452 | 1.366125 |
| 5 1.19477 .0863881 2.46 0.014 1.036903 | 1.376672 |
| 6 1.156307 .0831686 2.02 0.043 1.004267 | 1.331363 |
| 7 1.172082 .0863819 2.15 0.031 1.014436 | 1.354226 |
| 8 1.157667 .0930748 1.82 0.069 .988891 | 1.355249 |
| 9 1.221642 .0914086 2.68 0.007 1.055002 | 1.414603 |
| 10 1.142691 .0965117 1.58 0.114 .9683585 | 1.348408 |
| 11 1.041166 .083557 0.50 0.615 .8896278 | 1.218517 |
| 12 1.161866 .0947393 1.84 0.066 .990258 | 1.363212 |
| 13 1.077795 .0875206 0.92 0.356 .9192116 | 1.263736 |
| 14 1.192812 .0884131 2.38 0.017 1.031525 | 1.379318 |
| 15 1.112879 .0866867 1.37 0.170 .9553098 | 1.296437 |
| 16 1.207115 .0928914 2.45 0.014 1.038117 | 1.403626 |
| 17 1.086695 .0898475 1.01 0.315 .9241247 | 1.277864 |
| 18 1.210809 .1020915 2.27 0.023 1.026373 | 1.428388 |
| 19 1.158289 .0850462 2.00 0.045 1.00304 | 1.337567 |
| 20 1.143588 .0925448 1.66 0.097 .9758565 | 1.340148 |
| 21 1.129914 .0834628 1.65 0.098 .9776205 | 1.305933 |
| 22 1.205476 .0884675 2.55 0.011 1.043976 | 1.391959 |
| 23 1.206667 .1186117 1.91 0.056 .9952152 | 1.463046 |
| 24 1.183502 .0953683 2.09 0.037 1.010597 | 1.385989 |
| 25 1.138555 .082909 1.78 0.075 .9871201 | 1.313222 |
| 26 1.08895 .0815999 1.14 0.255 .9402072 | 1.261224 |
| 27 1.1893 .1032907 2.00 0.046 1.003147 | 1.409997 |
| 28 1.176346 .0974718 1.96 0.050 1.000011 | 1.383775 |
| 29 1.175755 .0991259 1.92 0.055 .9966749 | 1.387012 |
| 30 1.195439 .0899646 2.37 0.018 1.031499 | 1.385434 |
| 31 1.295643 .1013736 3.31 0.001 1.111439 | 1.510376 |
| 32 1.2058 .0974621 2.32 0.021 1.02914 | 1.412784 |
| 33 1.249106 .1018328 2.73 0.006 1.064646 | 1.465525 |
| 34 1.290318 .110163 2.99 0.003 1.091501 | 1.52535 |
| 35 1.290865 .1004025 3.28 0.001 1.108345 | 1.503441 |
| 36 1.179464 .0893312 2.18 0.029 1.016753 | 1.368213 |
| 37 1.241536 .1025751 2.62 0.009 1.055926 | 1.459773 |
| 38 1.199068 .0910922 2.39 0.017 1.033187 | 1.391582 |
| 39 1.165069 .1111552 1.60 0.109 .9663651 | 1.404629 |
| year |  |
| 2018 1.039713 .0164741 2.46 0.014 1.007921 | 1.072509 |
| 2019 1.051481 .0208946 2.53 0.012 1.011316 | 1.093242 |
| 2020 1 (omitted) |  |
| _cons .0006184 .0000452 -101.10 0.000 .0005359 | .0007136 |
| ln(popula~n) 1 (exposure) |  |
|  |  |
| /lnalpha -9.082617 5.777577 -20.40646 | 2.241226 |
|  |  |
| alpha .0001136 .0006565 1.37e-09 | 9.404857 |
|  |  |
| Note: Estimates are transformed only in the first equation. |  |
| Note: _cons estimates baseline incidence rate. |  |
| . |  |

**Table S45.- Oncologic diseases (excluding sex-specific cancer) (men, 20 to 29 years) (full specification)**

| Negative binomial regression Number of obs = | 156 |
| --- | --- |
| Wald chi2(43) = | 129.65 |
| Dispersion = mean Prob > chi2 = | 0.0000 |
| Log pseudolikelihood = -283.18825 Pseudo R2 = | 0.0935 |
|  |  |
| Robust |  |
| _freq IRR Std. Err. z P>z [95% Conf. | Interval] |
|  |  |
| t2 1.002569 .0009885 2.60 0.009 1.000633 | 1.004508 |
| 1.level .7016971 .1674476 -1.48 0.138 .4395675 | 1.120144 |
| slope .9995926 .012431 -0.03 0.974 .9755228 | 1.024256 |
| week |  |
| 2 1.087966 .3645628 0.25 0.801 .5641432 | 2.098174 |
| 3 1.717967 .5328591 1.74 0.081 .9354033 | 3.155227 |
| 4 .8115791 .2978351 -0.57 0.569 .3953257 | 1.666121 |
| 5 1.528848 .579572 1.12 0.263 .7272463 | 3.214007 |
| 6 .8968961 .3737663 -0.26 0.794 .3962947 | 2.02986 |
| 7 1.252268 .4105951 0.69 0.493 .6585734 | 2.381169 |
| 8 1.427303 .4942273 1.03 0.304 .724058 | 2.813577 |
| 9 .6227605 .2514208 -1.17 0.241 .2822761 | 1.373941 |
| 10 1.330888 .4016155 0.95 0.344 .7366795 | 2.404387 |
| 11 1.592758 .6169173 1.20 0.229 .7455159 | 3.402849 |
| 12 1.164457 .359404 0.49 0.622 .6359227 | 2.132273 |
| 13 .967835 .2780162 -0.11 0.909 .5511741 | 1.699471 |
| 14 1.254884 .5011437 0.57 0.570 .5736837 | 2.744953 |
| 15 1.155316 .3573424 0.47 0.641 .6301177 | 2.118262 |
| 16 .8642138 .3711933 -0.34 0.734 .3724062 | 2.005513 |
| 17 1.245033 .3848495 0.71 0.478 .6793099 | 2.281887 |
| 18 .7641642 .3742703 -0.55 0.583 .2926089 | 1.995657 |
| 19 .9526987 .375998 -0.12 0.902 .4395569 | 2.064886 |
| 20 .5701193 .2163671 -1.48 0.139 .2709721 | 1.199518 |
| 21 .9477054 .3494619 -0.15 0.884 .4600413 | 1.952315 |
| 22 1.134263 .3750558 0.38 0.703 .5932739 | 2.168565 |
| 23 1.037013 .4004494 0.09 0.925 .4865045 | 2.210453 |
| 24 1.504424 .4889748 1.26 0.209 .7956255 | 2.844669 |
| 25 1.969374 .6313291 2.11 0.035 1.050644 | 3.691483 |
| 26 1.403005 .4854697 0.98 0.328 .712074 | 2.76435 |
| 27 1.212747 .4862097 0.48 0.630 .5527256 | 2.660912 |
| 28 .9304342 .2807856 -0.24 0.811 .5150042 | 1.680972 |
| 29 1.20639 .4081088 0.55 0.579 .6216351 | 2.341208 |
| 30 1.573447 .5531349 1.29 0.197 .789983 | 3.133909 |
| 31 1.477004 .5557926 1.04 0.300 .7064428 | 3.088066 |
| 32 1.84141 .6943792 1.62 0.105 .8793664 | 3.855946 |
| 33 1.010118 .423569 0.02 0.981 .4440592 | 2.297752 |
| 34 1.831757 .5645887 1.96 0.050 1.001172 | 3.351409 |
| 35 .8221273 .3080892 -0.52 0.601 .3944159 | 1.713656 |
| 36 1.548831 .6867538 0.99 0.324 .6495013 | 3.693414 |
| 37 1.999109 .6342588 2.18 0.029 1.073433 | 3.723041 |
| 38 .6344104 .2965753 -0.97 0.330 .2537751 | 1.585957 |
| 39 1.536667 .4807331 1.37 0.170 .8323172 | 2.837075 |
| year |  |
| 2018 .9769104 .103498 -0.22 0.825 .7937338 | 1.20236 |
| 2019 1.041276 .1217778 0.35 0.729 .8279753 | 1.309527 |
| 2020 1 (omitted) |  |
| _cons 2.37e-06 6.69e-07 -45.82 0.000 1.36e-06 | 4.12e-06 |
| ln(popula~n) 1 (exposure) |  |
|  |  |
| /lnalpha -17.70552 .1099986 -17.92112 | -17.48993 |
|  |  |
| alpha 2.04e-08 2.25e-09 1.65e-08 | 2.54e-08 |
|  |  |
| Note: Estimates are transformed only in the first equation. |  |
| Note: _cons estimates baseline incidence rate. |  |

**Table S46.- Oncologic diseases (excluding sex-specific cancer) (women, 20 to 29 years) (full specification)**

| Negative binomial regression Number of obs = | 156 |
| --- | --- |
| Wald chi2(43) = | 223.04 |
| Dispersion = mean Prob > chi2 = | 0.0000 |
| Log pseudolikelihood = -273.21004 Pseudo R2 = | 0.1134 |
|  |  |
| Robust |  |
| _freq IRR Std. Err. z P>z [95% Conf. | Interval] |
|  |  |
| t2 1.003436 .0012191 2.82 0.005 1.00105 | 1.005828 |
| 1.level .3032606 .0907621 -3.99 0.000 .1686794 | .5452178 |
| slope 1.043637 .013237 3.37 0.001 1.018013 | 1.069906 |
| week |  |
| 2 1.577597 .6118175 1.18 0.240 .7377113 | 3.373694 |
| 3 1.571881 .6780516 1.05 0.294 .674905 | 3.660974 |
| 4 .9891702 .30527 -0.04 0.972 .5402314 | 1.811182 |
| 5 1.396248 .4522008 1.03 0.303 .7400907 | 2.634147 |
| 6 .9001809 .4825349 -0.20 0.844 .314811 | 2.574007 |
| 7 1.059996 .3867866 0.16 0.873 .5184483 | 2.16722 |
| 8 .9749128 .3861211 -0.06 0.949 .4485815 | 2.118801 |
| 9 .8904327 .2663564 -0.39 0.698 .4954273 | 1.600377 |
| 10 1.290483 .4399518 0.75 0.454 .6615464 | 2.517353 |
| 11 .9643555 .3863878 -0.09 0.928 .4397307 | 2.114889 |
| 12 .9203726 .3763144 -0.20 0.839 .4129822 | 2.051143 |
| 13 .9122077 .3516843 -0.24 0.812 .4284786 | 1.94204 |
| 14 1.004373 .345985 0.01 0.990 .5112983 | 1.972949 |
| 15 1.094568 .4442379 0.22 0.824 .494057 | 2.424983 |
| 16 1.379881 .4622799 0.96 0.336 .7156107 | 2.660764 |
| 17 1.46411 .5246023 1.06 0.287 .7254061 | 2.955058 |
| 18 .6764749 .2655083 -1.00 0.319 .3134478 | 1.45995 |
| 19 1.434881 .4429862 1.17 0.242 .7834789 | 2.627875 |
| 20 1.893313 .6036556 2.00 0.045 1.013512 | 3.536843 |
| 21 1.123917 .3289729 0.40 0.690 .6332656 | 1.994724 |
| 22 1.389619 .5016241 0.91 0.362 .6849035 | 2.819435 |
| 23 1.190931 .3916236 0.53 0.595 .6251429 | 2.26879 |
| 24 1.267927 .5469231 0.55 0.582 .544411 | 2.95299 |
| 25 1.611175 .4849198 1.58 0.113 .8932117 | 2.906239 |
| 26 1.768814 .5499165 1.83 0.067 .9617169 | 3.253246 |
| 27 .873591 .3215341 -0.37 0.713 .424634 | 1.797221 |
| 28 .6901222 .2124286 -1.20 0.228 .3774984 | 1.261644 |
| 29 1.021911 .3036684 0.07 0.942 .570785 | 1.829591 |
| 30 1.008496 .3117771 0.03 0.978 .5502051 | 1.848518 |
| 31 1.077851 .3376241 0.24 0.811 .5833513 | 1.991534 |
| 32 1.063017 .3095565 0.21 0.834 .600711 | 1.881113 |
| 33 .8868008 .3355608 -0.32 0.751 .4224116 | 1.861728 |
| 34 .8740071 .3184871 -0.37 0.712 .4278959 | 1.78522 |
| 35 1.487353 .4374239 1.35 0.177 .8357556 | 2.646967 |
| 36 1.079375 .3516711 0.23 0.815 .5699573 | 2.0441 |
| 37 .9108622 .2811133 -0.30 0.762 .497453 | 1.667836 |
| 38 .2988122 .14033 -2.57 0.010 .1190288 | .7501442 |
| 39 1.396357 .442135 1.05 0.292 .750718 | 2.597266 |
| year |  |
| 2018 1.044865 .1079651 0.42 0.671 .8533087 | 1.279423 |
| 2019 1.122393 .1402589 0.92 0.356 .8785674 | 1.433886 |
| 2020 1 (omitted) |  |
| _cons 2.03e-06 6.01e-07 -44.34 0.000 1.14e-06 | 3.63e-06 |
| ln(popula~n) 1 (exposure) |  |
|  |  |
| /lnalpha -17.51638 .0958983 -17.70434 | -17.32842 |
|  |  |
| alpha 2.47e-08 2.37e-09 2.05e-08 | 2.98e-08 |
|  |  |
| Note: Estimates are transformed only in the first equation. |  |
| Note: _cons estimates baseline incidence rate. |  |
| . |  |

**Table S46.- Oncologic diseases (excluding sex-specific cancer) (men, 30 to 39 years) (full specification)**

| Negative binomial regression Number of obs = | 156 |
| --- | --- |
| Wald chi2(43) = | 134.18 |
| Dispersion = mean Prob > chi2 = | 0.0000 |
| Log pseudolikelihood = -308.43881 Pseudo R2 = | 0.1096 |
|  |  |
| Robust |  |
| _freq IRR Std. Err. z P>z [95% Conf. | Interval] |
|  |  |
| t2 1.004181 .0010409 4.02 0.000 1.002143 | 1.006223 |
| 1.level .4303774 .1129858 -3.21 0.001 .257268 | .7199678 |
| slope 1.013493 .0136029 1.00 0.318 .9871798 | 1.040508 |
| week |  |
| 2 2.352072 .5823423 3.45 0.001 1.447785 | 3.821175 |
| 3 1.350324 .3507283 1.16 0.248 .8116153 | 2.246602 |
| 4 1.702044 .5338202 1.70 0.090 .920459 | 3.147293 |
| 5 1.248003 .2528056 1.09 0.274 .8390526 | 1.856273 |
| 6 1.330606 .3554369 1.07 0.285 .7882667 | 2.246084 |
| 7 2.20683 .4948194 3.53 0.000 1.422037 | 3.424734 |
| 8 1.405465 .313044 1.53 0.126 .9082998 | 2.174759 |
| 9 1.398593 .6193037 0.76 0.449 .5871854 | 3.331252 |
| 10 1.826679 .590928 1.86 0.063 .9689463 | 3.443695 |
| 11 1.558072 .4003639 1.73 0.084 .9415896 | 2.57818 |
| 12 1.497431 .6098384 0.99 0.321 .6740462 | 3.326627 |
| 13 1.698787 .3988222 2.26 0.024 1.072268 | 2.691377 |
| 14 1.580878 .4345145 1.67 0.096 .9224445 | 2.709297 |
| 15 2.510704 .6438142 3.59 0.000 1.518882 | 4.150183 |
| 16 1.765219 .472033 2.13 0.034 1.045155 | 2.981373 |
| 17 1.752073 .4592326 2.14 0.032 1.048203 | 2.928593 |
| 18 .818342 .274733 -0.60 0.550 .4238091 | 1.580154 |
| 19 2.23356 .5562267 3.23 0.001 1.37095 | 3.638929 |
| 20 1.410656 .3061442 1.59 0.113 .9219151 | 2.158495 |
| 21 1.199996 .2858597 0.77 0.444 .7523306 | 1.914038 |
| 22 2.183307 .5714129 2.98 0.003 1.307191 | 3.646619 |
| 23 1.181828 .4090519 0.48 0.629 .5997059 | 2.329005 |
| 24 1.85693 .5107324 2.25 0.024 1.083128 | 3.183546 |
| 25 1.35777 .3453161 1.20 0.229 .82479 | 2.235161 |
| 26 .9623695 .3316855 -0.11 0.911 .4897461 | 1.891092 |
| 27 2.291837 .6375249 2.98 0.003 1.328628 | 3.95334 |
| 28 1.610792 .4646263 1.65 0.098 .9151958 | 2.835078 |
| 29 1.692265 .4320328 2.06 0.039 1.026025 | 2.791123 |
| 30 1.772318 .4518204 2.24 0.025 1.075333 | 2.921057 |
| 31 1.573316 .5148455 1.38 0.166 .8284612 | 2.987856 |
| 32 1.83638 .5770981 1.93 0.053 .9918942 | 3.399851 |
| 33 1.730768 .4825163 1.97 0.049 1.002154 | 2.989119 |
| 34 1.897769 .4209798 2.89 0.004 1.228634 | 2.931328 |
| 35 1.703348 .3498242 2.59 0.010 1.138907 | 2.547525 |
| 36 1.067193 .4936151 0.14 0.888 .4310542 | 2.642131 |
| 37 1.411497 .3351671 1.45 0.147 .8862531 | 2.24803 |
| 38 .6125511 .2380219 -1.26 0.207 .2860138 | 1.311891 |
| 39 1.562382 .3751632 1.86 0.063 .9758754 | 2.501381 |
| year |  |
| 2018 1.034844 .0984937 0.36 0.719 .8587361 | 1.247067 |
| 2019 1.090611 .1255082 0.75 0.451 .8703888 | 1.366554 |
| 2020 1 (omitted) |  |
| _cons 2.45e-06 5.04e-07 -62.92 0.000 1.64e-06 | 3.67e-06 |
| ln(popula~n) 1 (exposure) |  |
|  |  |
| /lnalpha -16.88888 .2372209 -17.35382 | -16.42393 |
|  |  |
| alpha 4.63e-08 1.10e-08 2.91e-08 | 7.37e-08 |
|  |  |
| Note: Estimates are transformed only in the first equation. |  |
| Note: _cons estimates baseline incidence rate. |  |

**Table S46.- Oncologic diseases (excluding sex-specific cancer) (women, 30 to 39 years) (full specification)**

| Negative binomial regression Number of obs = | 156 |
| --- | --- |
| Wald chi2(43) = | 255.77 |
| Dispersion = mean Prob > chi2 = | 0.0000 |
| Log pseudolikelihood = -315.7882 Pseudo R2 = | 0.1292 |
|  |  |
| Robust |  |
| _freq IRR Std. Err. z P>z [95% Conf. | Interval] |
|  |  |
| t2 1.003973 .0008065 4.94 0.000 1.002394 | 1.005555 |
| 1.level .2533154 .0643006 -5.41 0.000 .1540266 | .4166077 |
| slope 1.030814 .0130022 2.41 0.016 1.005642 | 1.056615 |
| week |  |
| 2 1.617243 .7108293 1.09 0.274 .6833533 | 3.827412 |
| 3 1.609537 .6205324 1.23 0.217 .7560199 | 3.426642 |
| 4 1.293821 .5447469 0.61 0.541 .566874 | 2.952989 |
| 5 1.839495 .7470476 1.50 0.133 .8298746 | 4.077414 |
| 6 1.220496 .4960809 0.49 0.624 .5502477 | 2.707163 |
| 7 1.396885 .5716812 0.82 0.414 .626329 | 3.115434 |
| 8 1.148453 .4690226 0.34 0.735 .5158065 | 2.557054 |
| 9 1.263297 .5240568 0.56 0.573 .5602727 | 2.84847 |
| 10 1.317154 .514704 0.70 0.481 .6123682 | 2.83309 |
| 11 1.310884 .543467 0.65 0.514 .5816648 | 2.954309 |
| 12 1.765475 .7460296 1.35 0.179 .7712104 | 4.041573 |
| 13 1.511883 .642897 0.97 0.331 .6569958 | 3.479157 |
| 14 1.025634 .4934336 0.05 0.958 .399465 | 2.633337 |
| 15 1.798933 .7012943 1.51 0.132 .8378829 | 3.862305 |
| 16 1.705709 .7341299 1.24 0.215 .7337555 | 3.965139 |
| 17 1.229568 .4930108 0.52 0.606 .5603408 | 2.698066 |
| 18 1.14242 .4466848 0.34 0.733 .5308933 | 2.458355 |
| 19 1.283032 .5640002 0.57 0.571 .5420796 | 3.03677 |
| 20 1.495625 .6113018 0.98 0.325 .6712962 | 3.332204 |
| 21 .9631479 .4692712 -0.08 0.939 .3706509 | 2.50277 |
| 22 1.027498 .4041611 0.07 0.945 .475297 | 2.221247 |
| 23 1.453895 .5685172 0.96 0.339 .6755965 | 3.128807 |
| 24 1.00793 .451468 0.02 0.986 .4189515 | 2.424919 |
| 25 1.283271 .5084398 0.63 0.529 .590292 | 2.789778 |
| 26 1.341182 .5797196 0.68 0.497 .5748569 | 3.129072 |
| 27 .9084666 .3478919 -0.25 0.802 .4288905 | 1.924294 |
| 28 2.006023 .8707499 1.60 0.109 .8567552 | 4.696941 |
| 29 1.232327 .5404256 0.48 0.634 .5217222 | 2.910801 |
| 30 2.235722 .8868255 2.03 0.043 1.027492 | 4.86471 |
| 31 1.273662 .5366719 0.57 0.566 .5576869 | 2.908825 |
| 32 1.591622 .6786593 1.09 0.276 .6900684 | 3.671029 |
| 33 1.180783 .4683859 0.42 0.675 .5426507 | 2.569328 |
| 34 1.362444 .5392476 0.78 0.435 .627217 | 2.959507 |
| 35 1.283114 .5512716 0.58 0.562 .5527881 | 2.978323 |
| 36 1.26863 .5973835 0.51 0.613 .5040964 | 3.192685 |
| 37 1.504928 .5807014 1.06 0.289 .7064241 | 3.206018 |
| 38 .6817585 .2697648 -0.97 0.333 .31392 | 1.480615 |
| 39 1.408717 .5704558 0.85 0.397 .6369896 | 3.115411 |
| year |  |
| 2018 .9837734 .0822517 -0.20 0.845 .8350788 | 1.158945 |
| 2019 1.014887 .0832502 0.18 0.857 .8641606 | 1.191903 |
| 2020 1 (omitted) |  |
| _cons 3.05e-06 1.15e-06 -33.78 0.000 1.46e-06 | 6.38e-06 |
| ln(popula~n) 1 (exposure) |  |
|  |  |
| /lnalpha -18.09963 .1301956 -18.35481 | -17.84445 |
|  |  |
| alpha 1.38e-08 1.79e-09 1.07e-08 | 1.78e-08 |
|  |  |
| Note: Estimates are transformed only in the first equation. |  |
| Note: _cons estimates baseline incidence rate. |  |
| . |  |

**Table S47.- Oncologic diseases (excluding sex-specific cancer) (men, 40 to 49 years) (full specification)**

| Negative binomial regression Number of obs = | 156 |
| --- | --- |
| Wald chi2(43) = | 273.93 |
| Dispersion = mean Prob > chi2 = | 0.0000 |
| Log pseudolikelihood = -427.66798 Pseudo R2 = | 0.1251 |
|  |  |
| Robust |  |
| _freq IRR Std. Err. z P>z [95% Conf. | Interval] |
|  |  |
| t2 1.002941 .000457 6.45 0.000 1.002046 | 1.003837 |
| 1.level .3277276 .0537809 -6.80 0.000 .2375903 | .4520613 |
| slope 1.022993 .0092167 2.52 0.012 1.005087 | 1.041218 |
| week |  |
| 2 1.530526 .3111771 2.09 0.036 1.027495 | 2.279827 |
| 3 1.479935 .3225401 1.80 0.072 .9654505 | 2.268586 |
| 4 1.268208 .2888536 1.04 0.297 .8115523 | 1.981822 |
| 5 1.448551 .2955638 1.82 0.069 .9710776 | 2.160796 |
| 6 1.35273 .317827 1.29 0.198 .8535308 | 2.143891 |
| 7 1.668965 .3291451 2.60 0.009 1.133907 | 2.456501 |
| 8 1.231072 .2760073 0.93 0.354 .7933112 | 1.910397 |
| 9 1.477639 .3047928 1.89 0.058 .9862589 | 2.213838 |
| 10 1.745467 .3377638 2.88 0.004 1.194528 | 2.550509 |
| 11 1.830928 .3916421 2.83 0.005 1.203916 | 2.784494 |
| 12 1.581425 .3913209 1.85 0.064 .9736888 | 2.568484 |
| 13 1.486331 .436061 1.35 0.177 .8363526 | 2.641446 |
| 14 1.988884 .4150031 3.30 0.001 1.321285 | 2.993796 |
| 15 1.383821 .3399133 1.32 0.186 .8550589 | 2.239566 |
| 16 1.29118 .3182247 1.04 0.300 .796525 | 2.093024 |
| 17 1.785361 .364982 2.84 0.005 1.195954 | 2.665247 |
| 18 1.108906 .2321525 0.49 0.621 .7356877 | 1.671461 |
| 19 1.515157 .3897628 1.62 0.106 .9151505 | 2.508551 |
| 20 1.697139 .3993461 2.25 0.025 1.070101 | 2.691595 |
| 21 1.251078 .2611782 1.07 0.283 .8309702 | 1.883577 |
| 22 1.621245 .3381246 2.32 0.021 1.077267 | 2.439911 |
| 23 1.798509 .3621716 2.91 0.004 1.212003 | 2.668836 |
| 24 1.59992 .349238 2.15 0.031 1.043023 | 2.454158 |
| 25 1.456757 .3799342 1.44 0.149 .873749 | 2.428776 |
| 26 1.210118 .2872642 0.80 0.422 .7599153 | 1.927039 |
| 27 1.619849 .3326656 2.35 0.019 1.08309 | 2.422615 |
| 28 1.375114 .2657954 1.65 0.099 .9414778 | 2.008478 |
| 29 1.133606 .275562 0.52 0.606 .7039602 | 1.825475 |
| 30 1.611618 .3689524 2.08 0.037 1.02895 | 2.524238 |
| 31 1.676268 .3770883 2.30 0.022 1.078598 | 2.605117 |
| 32 1.638914 .3618491 2.24 0.025 1.063219 | 2.526326 |
| 33 1.451704 .3811545 1.42 0.156 .8677401 | 2.42866 |
| 34 1.639705 .4339503 1.87 0.062 .9760989 | 2.754466 |
| 35 2.071267 .4103505 3.68 0.000 1.404752 | 3.054024 |
| 36 1.786397 .3592144 2.89 0.004 1.204525 | 2.649356 |
| 37 1.578445 .3349011 2.15 0.031 1.041426 | 2.39238 |
| 38 .7228697 .292815 -0.80 0.423 .3267843 | 1.599038 |
| 39 1.649577 .3610224 2.29 0.022 1.074189 | 2.533171 |
| year |  |
| 2018 .9143795 .0490521 -1.67 0.095 .8231208 | 1.015756 |
| 2019 .9185391 .0516753 -1.51 0.131 .8226416 | 1.025616 |
| 2020 1 (omitted) |  |
| _cons .0000118 2.19e-06 -61.42 0.000 8.25e-06 | .000017 |
| ln(popula~n) 1 (exposure) |  |
|  |  |
| /lnalpha -20.38361 . . | . |
|  |  |
| alpha 1.40e-09 . . | . |
|  |  |
| Note: Estimates are transformed only in the first equation. |  |
| Note: _cons estimates baseline incidence rate. |  |

**Table S48.- Oncologic diseases (excluding sex-specific cancer) (women, 40 to 49 years) (full specification)**

| Negative binomial regression Number of obs = | 156 |
| --- | --- |
| Wald chi2(43) = | 408.15 |
| Dispersion = mean Prob > chi2 = | 0.0000 |
| Log pseudolikelihood = -477.96258 Pseudo R2 = | 0.1772 |
|  |  |
| Robust |  |
| _freq IRR Std. Err. z P>z [95% Conf. | Interval] |
|  |  |
| t2 1.002312 .0004458 5.19 0.000 1.001439 | 1.003186 |
| 1.level .274274 .0579805 -6.12 0.000 .1812357 | .415074 |
| slope 1.027349 .0116503 2.38 0.017 1.004766 | 1.050438 |
| week |  |
| 2 1.840477 .4147255 2.71 0.007 1.183379 | 2.862441 |
| 3 1.611993 .3865307 1.99 0.046 1.007531 | 2.579099 |
| 4 1.678069 .3827177 2.27 0.023 1.073188 | 2.623878 |
| 5 1.617578 .3684212 2.11 0.035 1.03513 | 2.52776 |
| 6 1.349481 .3171658 1.28 0.202 .8513548 | 2.13906 |
| 7 1.276915 .2918823 1.07 0.285 .8158141 | 1.998633 |
| 8 1.412592 .3130913 1.56 0.119 .9148585 | 2.181121 |
| 9 1.326078 .3157362 1.19 0.236 .8315718 | 2.114648 |
| 10 1.998108 .4564723 3.03 0.002 1.276909 | 3.12664 |
| 11 1.760099 .4106013 2.42 0.015 1.114208 | 2.780405 |
| 12 1.709513 .4976942 1.84 0.066 .9661851 | 3.024715 |
| 13 1.471936 .4961916 1.15 0.251 .7602344 | 2.849903 |
| 14 1.775576 .3981288 2.56 0.010 1.144138 | 2.755497 |
| 15 1.38862 .3584036 1.27 0.203 .8373127 | 2.302921 |
| 16 1.500949 .3560455 1.71 0.087 .9428645 | 2.389365 |
| 17 1.899973 .40867 2.98 0.003 1.246408 | 2.89624 |
| 18 1.316184 .2866457 1.26 0.207 .8588893 | 2.016954 |
| 19 1.79484 .4228225 2.48 0.013 1.131104 | 2.848059 |
| 20 1.618931 .3527912 2.21 0.027 1.05618 | 2.481527 |
| 21 1.658845 .3591523 2.34 0.019 1.085212 | 2.535697 |
| 22 1.73106 .417259 2.28 0.023 1.079285 | 2.776437 |
| 23 1.819868 .4159852 2.62 0.009 1.162713 | 2.848442 |
| 24 1.744604 .3778606 2.57 0.010 1.141134 | 2.667209 |
| 25 1.55538 .3345946 2.05 0.040 1.020294 | 2.371087 |
| 26 1.690793 .4024605 2.21 0.027 1.060421 | 2.695892 |
| 27 1.663822 .3702748 2.29 0.022 1.075663 | 2.573577 |
| 28 2.08323 .4667667 3.28 0.001 1.34282 | 3.231891 |
| 29 1.343171 .3167071 1.25 0.211 .8461084 | 2.132243 |
| 30 1.868943 .4242939 2.75 0.006 1.197715 | 2.916344 |
| 31 1.98 .4415049 3.06 0.002 1.278976 | 3.065265 |
| 32 1.843867 .4466873 2.53 0.012 1.146889 | 2.964409 |
| 33 1.492533 .3537123 1.69 0.091 .9379925 | 2.374917 |
| 34 1.878881 .4215771 2.81 0.005 1.210346 | 2.916682 |
| 35 2.124901 .4997462 3.20 0.001 1.340135 | 3.369218 |
| 36 1.5672 .3484043 2.02 0.043 1.013664 | 2.42301 |
| 37 1.870494 .4234452 2.77 0.006 1.200218 | 2.915094 |
| 38 .6833116 .2035248 -1.28 0.201 .3811429 | 1.225039 |
| 39 1.518377 .3653507 1.74 0.083 .9474672 | 2.433297 |
| year |  |
| 2018 .9784434 .0383931 -0.56 0.579 .9060152 | 1.056662 |
| 2019 1.084658 .0526971 1.67 0.094 .9861385 | 1.19302 |
| 2020 1 (omitted) |  |
| _cons .0000164 3.48e-06 -52.06 0.000 .0000109 | .0000249 |
| ln(popula~n) 1 (exposure) |  |
|  |  |
| /lnalpha -7.843429 13.25554 -33.82381 | 18.13695 |
|  |  |
| alpha .0003923 .0052004 2.04e-15 | 7.53e+07 |
|  |  |
| Note: Estimates are transformed only in the first equation. |  |
| Note: _cons estimates baseline incidence rate. |  |
| . |  |

**Table S49.- Oncologic diseases (excluding sex-specific cancer) (men, 50 to 59 years) (full specification)**

| Negative binomial regression Number of obs = | 156 |
| --- | --- |
| Wald chi2(43) = | 404.83 |
| Dispersion = mean Prob > chi2 = | 0.0000 |
| Log pseudolikelihood = -472.63206 Pseudo R2 = | 0.1824 |
|  |  |
| Robust |  |
| _freq IRR Std. Err. z P>z [95% Conf. | Interval] |
|  |  |
| t2 1.002027 .0003592 5.65 0.000 1.001323 | 1.002731 |
| 1.level .3481637 .0429094 -8.56 0.000 .2734497 | .4432918 |
| slope 1.026388 .0064836 4.12 0.000 1.013758 | 1.039174 |
| week |  |
| 2 1.337398 .19965 1.95 0.051 .9981398 | 1.791968 |
| 3 1.249648 .2149128 1.30 0.195 .8920701 | 1.750557 |
| 4 1.415892 .2287707 2.15 0.031 1.03157 | 1.943398 |
| 5 1.349436 .2366227 1.71 0.087 .9569616 | 1.902873 |
| 6 1.241192 .2128133 1.26 0.208 .8869372 | 1.73694 |
| 7 1.059968 .2007197 0.31 0.758 .7313172 | 1.536313 |
| 8 1.120395 .1613375 0.79 0.430 .8448852 | 1.485746 |
| 9 1.358157 .2032467 2.05 0.041 1.012905 | 1.82109 |
| 10 1.667794 .2463813 3.46 0.001 1.24852 | 2.227868 |
| 11 1.404017 .2054072 2.32 0.020 1.054003 | 1.870264 |
| 12 1.477987 .2649595 2.18 0.029 1.040101 | 2.100226 |
| 13 1.127766 .187343 0.72 0.469 .8143637 | 1.561778 |
| 14 1.386121 .1978478 2.29 0.022 1.047864 | 1.833569 |
| 15 1.24048 .1828314 1.46 0.144 .9292514 | 1.655947 |
| 16 1.158641 .1996602 0.85 0.393 .8265471 | 1.624166 |
| 17 1.449223 .2426181 2.22 0.027 1.043839 | 2.012042 |
| 18 1.280634 .2452809 1.29 0.197 .8798198 | 1.864045 |
| 19 1.38319 .1947015 2.30 0.021 1.049698 | 1.822633 |
| 20 1.387022 .2028698 2.24 0.025 1.04132 | 1.847492 |
| 21 1.112511 .2110052 0.56 0.574 .767115 | 1.613422 |
| 22 1.502216 .2286432 2.67 0.008 1.114747 | 2.024364 |
| 23 1.373365 .1929951 2.26 0.024 1.042723 | 1.808852 |
| 24 1.127294 .1907472 0.71 0.479 .8091092 | 1.570606 |
| 25 1.261503 .2103539 1.39 0.164 .9098114 | 1.749141 |
| 26 1.335234 .2371507 1.63 0.104 .9427042 | 1.891207 |
| 27 1.535811 .3033484 2.17 0.030 1.042824 | 2.261852 |
| 28 1.421461 .2230027 2.24 0.025 1.045192 | 1.933186 |
| 29 1.067425 .1624735 0.43 0.668 .7920919 | 1.438466 |
| 30 1.401991 .2054841 2.31 0.021 1.051933 | 1.86854 |
| 31 1.675127 .2717114 3.18 0.001 1.218933 | 2.302054 |
| 32 1.798051 .2625057 4.02 0.000 1.350614 | 2.393717 |
| 33 1.249539 .2059884 1.35 0.177 .9045399 | 1.726122 |
| 34 1.395443 .1976183 2.35 0.019 1.057225 | 1.84186 |
| 35 1.451038 .219092 2.47 0.014 1.079335 | 1.95075 |
| 36 1.40737 .2085785 2.31 0.021 1.052583 | 1.881743 |
| 37 1.61315 .2424392 3.18 0.001 1.201567 | 2.165714 |
| 38 .5908359 .1517453 -2.05 0.040 .3571507 | .9774223 |
| 39 1.577412 .2596815 2.77 0.006 1.142395 | 2.178082 |
| year |  |
| 2018 1.024681 .0366649 0.68 0.496 .9552808 | 1.099122 |
| 2019 1.124062 .0469635 2.80 0.005 1.035683 | 1.219982 |
| 2020 1 (omitted) |  |
| _cons .0000249 3.40e-06 -77.57 0.000 .000019 | .0000325 |
| ln(popula~n) 1 (exposure) |  |
|  |  |
| /lnalpha -17.70393 .2350267 -18.16457 | -17.24328 |
|  |  |
| alpha 2.05e-08 4.81e-09 1.29e-08 | 3.25e-08 |
|  |  |
| Note: Estimates are transformed only in the first equation. |  |
| Note: _cons estimates baseline incidence rate. |  |

**Table S50.- Oncologic diseases (excluding sex-specific cancer) (women, 50 to 59 years) (full specification)**

| Negative binomial regression Number of obs = | 156 |
| --- | --- |
| Wald chi2(43) = | 725.84 |
| Dispersion = mean Prob > chi2 = | 0.0000 |
| Log pseudolikelihood = -540.10147 Pseudo R2 = | 0.1924 |
|  |  |
| Robust |  |
| _freq IRR Std. Err. z P>z [95% Conf. | Interval] |
|  |  |
| t2 1.002489 .0003686 6.76 0.000 1.001767 | 1.003212 |
| 1.level .2393103 .0368758 -9.28 0.000 .1769286 | .3236867 |
| slope 1.030095 .0080934 3.77 0.000 1.014354 | 1.04608 |
| week |  |
| 2 1.646541 .3256739 2.52 0.012 1.117406 | 2.426242 |
| 3 1.663488 .3222488 2.63 0.009 1.137958 | 2.431719 |
| 4 1.48901 .3033151 1.95 0.051 .9988629 | 2.219676 |
| 5 1.602023 .3257862 2.32 0.020 1.075398 | 2.386539 |
| 6 1.293245 .25855 1.29 0.198 .8739886 | 1.913621 |
| 7 1.343228 .2740715 1.45 0.148 .9004734 | 2.00368 |
| 8 1.224611 .2511758 0.99 0.323 .8192396 | 1.830565 |
| 9 1.225177 .2807191 0.89 0.375 .7819288 | 1.919689 |
| 10 1.535776 .2961847 2.22 0.026 1.052368 | 2.241237 |
| 11 1.658073 .3392237 2.47 0.013 1.110343 | 2.475997 |
| 12 1.770064 .3857164 2.62 0.009 1.154789 | 2.713158 |
| 13 1.210261 .2675352 0.86 0.388 .784722 | 1.86656 |
| 14 1.39769 .2869235 1.63 0.103 .9347009 | 2.090013 |
| 15 1.559375 .3774537 1.84 0.066 .9703165 | 2.506038 |
| 16 1.479945 .3112167 1.86 0.062 .9800471 | 2.234829 |
| 17 1.448841 .3151113 1.70 0.088 .9460002 | 2.218964 |
| 18 1.365162 .2862396 1.48 0.138 .9051258 | 2.059014 |
| 19 1.78419 .4226813 2.44 0.015 1.121471 | 2.838533 |
| 20 1.804079 .3455585 3.08 0.002 1.239407 | 2.626014 |
| 21 1.24066 .2376794 1.13 0.260 .8522832 | 1.806017 |
| 22 1.525147 .2936129 2.19 0.028 1.045787 | 2.224233 |
| 23 1.650707 .3202013 2.58 0.010 1.12864 | 2.414263 |
| 24 1.244969 .243857 1.12 0.263 .8480675 | 1.827624 |
| 25 1.549905 .3090199 2.20 0.028 1.048558 | 2.29096 |
| 26 1.345868 .2584645 1.55 0.122 .9237089 | 1.960965 |
| 27 1.502945 .310046 1.97 0.048 1.003106 | 2.25185 |
| 28 1.69078 .3368867 2.64 0.008 1.144158 | 2.498552 |
| 29 1.298246 .2593345 1.31 0.191 .8776531 | 1.920397 |
| 30 1.691153 .3572609 2.49 0.013 1.1178 | 2.558596 |
| 31 1.696464 .3312675 2.71 0.007 1.156994 | 2.487473 |
| 32 1.698992 .3392186 2.65 0.008 1.148792 | 2.512703 |
| 33 1.433818 .283517 1.82 0.068 .9731526 | 2.11255 |
| 34 1.632348 .3314925 2.41 0.016 1.09636 | 2.430369 |
| 35 1.57797 .3110438 2.31 0.021 1.072291 | 2.322119 |
| 36 1.797899 .3524234 2.99 0.003 1.224371 | 2.640083 |
| 37 1.753335 .3475269 2.83 0.005 1.18891 | 2.585716 |
| 38 .6419002 .2015993 -1.41 0.158 .3468436 | 1.187959 |
| 39 1.519103 .2910039 2.18 0.029 1.043587 | 2.211292 |
| year |  |
| 2018 .9686783 .0342606 -0.90 0.368 .9038034 | 1.03821 |
| 2019 1.030271 .0414289 0.74 0.458 .9521896 | 1.114756 |
| 2020 1 (omitted) |  |
| _cons .0000325 6.13e-06 -54.82 0.000 .0000225 | .0000471 |
| ln(popula~n) 1 (exposure) |  |
|  |  |
| /lnalpha -5.753163 1.183861 -8.073488 | -3.432838 |
|  |  |
| alpha .0031727 .0037561 .0003117 | .0322952 |
|  |  |
| Note: Estimates are transformed only in the first equation. |  |
| Note: _cons estimates baseline incidence rate. |  |
| . |  |

**Table S51.- Oncologic diseases (excluding sex-specific cancer) (men, 60 to 69 years) (full specification)**

| Negative binomial regression Number of obs = | 156 |
| --- | --- |
| Wald chi2(43) = | 426.30 |
| Dispersion = mean Prob > chi2 = | 0.0000 |
| Log pseudolikelihood = -524.66969 Pseudo R2 = | 0.1562 |
|  |  |
| Robust |  |
| _freq IRR Std. Err. z P>z [95% Conf. | Interval] |
|  |  |
| t2 1.001673 .0004359 3.84 0.000 1.000819 | 1.002528 |
| 1.level .360548 .0645478 -5.70 0.000 .2538488 | .5120955 |
| slope 1.024273 .0091783 2.68 0.007 1.006441 | 1.042421 |
| week |  |
| 2 1.665192 .3747287 2.27 0.023 1.071305 | 2.588306 |
| 3 1.458973 .3424752 1.61 0.108 .9209556 | 2.311298 |
| 4 1.893274 .4291332 2.82 0.005 1.214167 | 2.952219 |
| 5 1.641359 .3607694 2.25 0.024 1.066866 | 2.525207 |
| 6 1.388212 .3112324 1.46 0.143 .8945802 | 2.154231 |
| 7 1.447783 .345096 1.55 0.121 .9074223 | 2.309922 |
| 8 1.250261 .2754136 1.01 0.311 .8118834 | 1.92534 |
| 9 1.663079 .3811536 2.22 0.026 1.06128 | 2.606129 |
| 10 1.745532 .3892608 2.50 0.012 1.127474 | 2.702398 |
| 11 1.746379 .4141272 2.35 0.019 1.097208 | 2.779637 |
| 12 1.903716 .521446 2.35 0.019 1.112884 | 3.256525 |
| 13 1.369584 .3370825 1.28 0.201 .8454554 | 2.21864 |
| 14 1.502251 .3645432 1.68 0.094 .933654 | 2.417124 |
| 15 1.454909 .3464952 1.57 0.115 .9122567 | 2.320355 |
| 16 1.547246 .3370883 2.00 0.045 1.009517 | 2.371401 |
| 17 1.250075 .3201263 0.87 0.383 .7567558 | 2.064984 |
| 18 1.277299 .2862249 1.09 0.275 .8232846 | 1.981686 |
| 19 1.610189 .3512404 2.18 0.029 1.050023 | 2.469191 |
| 20 1.331876 .2951605 1.29 0.196 .8626345 | 2.056368 |
| 21 1.43354 .3326944 1.55 0.121 .9096282 | 2.259206 |
| 22 1.580693 .3463327 2.09 0.037 1.02884 | 2.428552 |
| 23 1.422285 .3340834 1.50 0.134 .897524 | 2.25386 |
| 24 1.318603 .3355056 1.09 0.277 .8008184 | 2.17117 |
| 25 1.565711 .346496 2.03 0.043 1.014702 | 2.415932 |
| 26 1.493627 .3377371 1.77 0.076 .9588915 | 2.326563 |
| 27 1.391238 .3042712 1.51 0.131 .9062315 | 2.135816 |
| 28 1.727795 .3963509 2.38 0.017 1.102121 | 2.708665 |
| 29 1.231441 .283078 0.91 0.365 .784772 | 1.932342 |
| 30 1.613476 .365425 2.11 0.035 1.035094 | 2.515042 |
| 31 1.72329 .3757826 2.50 0.013 1.123943 | 2.642241 |
| 32 1.740097 .3779555 2.55 0.011 1.136814 | 2.66353 |
| 33 1.2999 .3055582 1.12 0.265 .8200191 | 2.06061 |
| 34 1.626935 .3658568 2.16 0.030 1.047023 | 2.52804 |
| 35 1.611238 .3628779 2.12 0.034 1.036227 | 2.505328 |
| 36 1.543708 .3341475 2.01 0.045 1.009987 | 2.359469 |
| 37 1.753783 .3853433 2.56 0.011 1.140115 | 2.697757 |
| 38 .6543333 .2226573 -1.25 0.213 .3358545 | 1.274814 |
| 39 1.600153 .356953 2.11 0.035 1.033429 | 2.477666 |
| year |  |
| 2018 1.025437 .0373739 0.69 0.491 .9547402 | 1.101368 |
| 2019 1.036582 .050304 0.74 0.459 .9425314 | 1.140017 |
| 2020 1 (omitted) |  |
| _cons .0000452 9.56e-06 -47.29 0.000 .0000299 | .0000684 |
| ln(popula~n) 1 (exposure) |  |
|  |  |
| /lnalpha -5.647841 1.492424 -8.572939 | -2.722743 |
|  |  |
| alpha .0035251 .005261 .0001892 | .0656943 |
|  |  |
| Note: Estimates are transformed only in the first equation. |  |
| Note: _cons estimates baseline incidence rate. |  |

**Table S52.- Oncologic diseases (excluding sex-specific cancer) (women, 60 to 69 years) (full specification)**

| Negative binomial regression Number of obs = | 156 |
| --- | --- |
| Wald chi2(43) = | 910.88 |
| Dispersion = mean Prob > chi2 = | 0.0000 |
| Log pseudolikelihood = -548.00915 Pseudo R2 = | 0.1990 |
|  |  |
| Robust |  |
| _freq IRR Std. Err. z P>z [95% Conf. | Interval] |
|  |  |
| t2 1.002145 .0003226 6.66 0.000 1.001513 | 1.002777 |
| 1.level .2594638 .0364685 -9.60 0.000 .1969871 | .3417557 |
| slope 1.025891 .0077962 3.36 0.001 1.010724 | 1.041285 |
| week |  |
| 2 1.871043 .4646284 2.52 0.012 1.150028 | 3.044102 |
| 3 1.668087 .4234211 2.02 0.044 1.014268 | 2.743371 |
| 4 1.871059 .4645865 2.52 0.012 1.150093 | 3.043982 |
| 5 2.011631 .5123275 2.74 0.006 1.22113 | 3.313863 |
| 6 1.43517 .3644583 1.42 0.155 .8724544 | 2.360827 |
| 7 1.304102 .3249508 1.07 0.287 .8002259 | 2.125252 |
| 8 1.20078 .308216 0.71 0.476 .7260677 | 1.985865 |
| 9 1.422507 .3666533 1.37 0.172 .8583333 | 2.357507 |
| 10 1.514854 .3807244 1.65 0.098 .9256366 | 2.47914 |
| 11 1.56201 .3914014 1.78 0.075 .9558588 | 2.552549 |
| 12 1.881864 .5020809 2.37 0.018 1.115547 | 3.174597 |
| 13 1.660841 .4715862 1.79 0.074 .9519945 | 2.897489 |
| 14 1.550657 .3871659 1.76 0.079 .9505805 | 2.529546 |
| 15 1.708169 .4419705 2.07 0.039 1.028707 | 2.836418 |
| 16 1.514007 .3850429 1.63 0.103 .9197077 | 2.492331 |
| 17 1.875478 .4721022 2.50 0.012 1.145103 | 3.071705 |
| 18 1.243271 .3285019 0.82 0.410 .7407264 | 2.086765 |
| 19 1.752089 .4383536 2.24 0.025 1.072988 | 2.860998 |
| 20 1.746461 .4326644 2.25 0.024 1.074692 | 2.838139 |
| 21 1.508045 .3776099 1.64 0.101 .9231584 | 2.463501 |
| 22 1.636784 .4081222 1.98 0.048 1.004036 | 2.668291 |
| 23 1.321685 .3430342 1.07 0.283 .794702 | 2.19812 |
| 24 1.407368 .3533739 1.36 0.174 .8603613 | 2.302155 |
| 25 1.400226 .3702918 1.27 0.203 .8338666 | 2.351254 |
| 26 1.4057 .3637345 1.32 0.188 .8465219 | 2.334247 |
| 27 1.645226 .4200225 1.95 0.051 .9975061 | 2.713534 |
| 28 1.596513 .4176023 1.79 0.074 .9561424 | 2.665769 |
| 29 1.261348 .344641 0.85 0.395 .7383448 | 2.154818 |
| 30 1.54488 .3989464 1.68 0.092 .9312842 | 2.562755 |
| 31 1.793603 .4506348 2.33 0.020 1.096139 | 2.934857 |
| 32 1.7822 .4860772 2.12 0.034 1.044239 | 3.041677 |
| 33 1.470951 .3864631 1.47 0.142 .8789456 | 2.461696 |
| 34 1.622546 .4105936 1.91 0.056 .9880895 | 2.664391 |
| 35 1.794168 .4486741 2.34 0.019 1.099006 | 2.929046 |
| 36 1.919894 .4811293 2.60 0.009 1.174801 | 3.137546 |
| 37 1.604917 .3985899 1.90 0.057 .9863974 | 2.611278 |
| 38 .6438363 .2193863 -1.29 0.196 .330164 | 1.255513 |
| 39 1.727715 .4465435 2.12 0.034 1.041049 | 2.867298 |
| year |  |
| 2018 .9893499 .0358789 -0.30 0.768 .9214695 | 1.062231 |
| 2019 1.012759 .034042 0.38 0.706 .9481877 | 1.081727 |
| 2020 1 (omitted) |  |
| _cons .0000497 .0000121 -40.55 0.000 .0000308 | .0000802 |
| ln(popula~n) 1 (exposure) |  |
|  |  |
| /lnalpha -5.801606 1.359305 -8.465795 | -3.137418 |
|  |  |
| alpha .0030227 .0041088 .0002105 | .0433947 |
|  |  |
| Note: Estimates are transformed only in the first equation. |  |
| Note: _cons estimates baseline incidence rate. |  |
| . |  |

**Table S53.- Oncologic diseases (excluding sex-specific cancer) (men, 70 to 79 years) (full specification)**

| Negative binomial regression Number of obs = | 156 |
| --- | --- |
| Wald chi2(43) = | 812.96 |
| Dispersion = mean Prob > chi2 = | 0.0000 |
| Log pseudolikelihood = -500.94234 Pseudo R2 = | 0.1973 |
|  |  |
| Robust |  |
| _freq IRR Std. Err. z P>z [95% Conf. | Interval] |
|  |  |
| t2 1.001807 .0003324 5.44 0.000 1.001156 | 1.002459 |
| 1.level .3195881 .0421686 -8.65 0.000 .2467615 | .413908 |
| slope 1.031814 .0076604 4.22 0.000 1.016908 | 1.046938 |
| week |  |
| 2 1.739179 .3867721 2.49 0.013 1.124728 | 2.689312 |
| 3 1.819804 .406906 2.68 0.007 1.174078 | 2.82067 |
| 4 1.509824 .3350377 1.86 0.063 .9773283 | 2.33245 |
| 5 1.77674 .4005667 2.55 0.011 1.142142 | 2.763933 |
| 6 1.493736 .3491504 1.72 0.086 .9447382 | 2.361763 |
| 7 1.422604 .3476802 1.44 0.149 .8811557 | 2.296757 |
| 8 1.393806 .3110239 1.49 0.137 .9000343 | 2.158468 |
| 9 1.19764 .2738741 0.79 0.430 .7650242 | 1.874897 |
| 10 1.779393 .3958371 2.59 0.010 1.150579 | 2.751864 |
| 11 1.574891 .3767449 1.90 0.058 .985431 | 2.51695 |
| 12 1.485516 .3764564 1.56 0.118 .9039985 | 2.441109 |
| 13 1.300757 .3025832 1.13 0.258 .8244962 | 2.052123 |
| 14 1.333513 .3162076 1.21 0.225 .8378324 | 2.12245 |
| 15 1.375849 .3172628 1.38 0.166 .8755657 | 2.161986 |
| 16 1.427556 .3210823 1.58 0.114 .9186351 | 2.218417 |
| 17 1.508526 .3373832 1.84 0.066 .9731524 | 2.338432 |
| 18 1.058771 .2357128 0.26 0.798 .6843848 | 1.637963 |
| 19 1.487925 .3299545 1.79 0.073 .9634364 | 2.297942 |
| 20 1.388816 .3083249 1.48 0.139 .8988215 | 2.145934 |
| 21 1.212636 .2749538 0.85 0.395 .7775507 | 1.891178 |
| 22 1.368867 .3564991 1.21 0.228 .8216366 | 2.280567 |
| 23 1.252738 .2921763 0.97 0.334 .7931117 | 1.978728 |
| 24 1.578064 .3522254 2.04 0.041 1.018909 | 2.44407 |
| 25 1.518529 .3583752 1.77 0.077 .9561769 | 2.411615 |
| 26 1.563201 .3673053 1.90 0.057 .9862976 | 2.477546 |
| 27 1.195799 .2793259 0.77 0.444 .7565311 | 1.890122 |
| 28 1.417838 .3186621 1.55 0.120 .9126771 | 2.202602 |
| 29 1.222378 .3023883 0.81 0.417 .7527273 | 1.985058 |
| 30 1.531174 .3461196 1.88 0.059 .9831313 | 2.38472 |
| 31 1.518157 .3503769 1.81 0.070 .965755 | 2.386526 |
| 32 1.28106 .2973654 1.07 0.286 .812802 | 2.019083 |
| 33 1.385224 .34978 1.29 0.197 .8444714 | 2.272245 |
| 34 1.566416 .3495139 2.01 0.044 1.01153 | 2.425691 |
| 35 1.621894 .3721091 2.11 0.035 1.034504 | 2.542802 |
| 36 1.580922 .3640305 1.99 0.047 1.00672 | 2.48263 |
| 37 1.55743 .356969 1.93 0.053 .9938252 | 2.44066 |
| 38 .5084925 .1335123 -2.58 0.010 .3039407 | .8507075 |
| 39 1.384697 .3418848 1.32 0.187 .8534754 | 2.246561 |
| year |  |
| 2018 1.040272 .0355225 1.16 0.248 .9729283 | 1.112278 |
| 2019 1.059977 .0394582 1.56 0.118 .9853942 | 1.140205 |
| 2020 1 (omitted) |  |
| _cons .0000778 .0000169 -43.48 0.000 .0000508 | .0001192 |
| ln(popula~n) 1 (exposure) |  |
|  |  |
| /lnalpha -18.1358 1.255278 -20.5961 | -15.6755 |
|  |  |
| alpha 1.33e-08 1.67e-08 1.14e-09 | 1.56e-07 |
|  |  |
| Note: Estimates are transformed only in the first equation. |  |
| Note: _cons estimates baseline incidence rate. |  |

**Table S54.- Oncologic diseases (excluding sex-specific cancer) (women, 70 to 79 years) (full specification)**

| Negative binomial regression Number of obs = | 156 |
| --- | --- |
| Wald chi2(43) = | 904.84 |
| Dispersion = mean Prob > chi2 = | 0.0000 |
| Log pseudolikelihood = -547.07256 Pseudo R2 = | 0.1914 |
|  |  |
| Robust |  |
| _freq IRR Std. Err. z P>z [95% Conf. | Interval] |
|  |  |
| t2 1.002039 .0003666 5.57 0.000 1.00132 | 1.002757 |
| 1.level .2449935 .0514627 -6.70 0.000 .162313 | .3697904 |
| slope 1.031389 .0114002 2.80 0.005 1.009285 | 1.053976 |
| week |  |
| 2 1.864267 .3842694 3.02 0.003 1.244673 | 2.792292 |
| 3 1.93599 .3968675 3.22 0.001 1.295423 | 2.893309 |
| 4 1.819784 .376315 2.90 0.004 1.213386 | 2.729233 |
| 5 1.859014 .3840877 3.00 0.003 1.239988 | 2.787071 |
| 6 1.506632 .3159312 1.95 0.051 .9988858 | 2.272474 |
| 7 1.653996 .3566037 2.33 0.020 1.083962 | 2.523798 |
| 8 1.305427 .3244505 1.07 0.284 .8020387 | 2.124761 |
| 9 1.730004 .363371 2.61 0.009 1.1462 | 2.611163 |
| 10 1.746173 .3659196 2.66 0.008 1.158013 | 2.633061 |
| 11 1.9504 .4469696 2.92 0.004 1.244673 | 3.056272 |
| 12 1.998059 .5436005 2.54 0.011 1.172268 | 3.405571 |
| 13 1.836865 .4160649 2.68 0.007 1.178346 | 2.863395 |
| 14 1.629297 .3430679 2.32 0.020 1.078375 | 2.461676 |
| 15 1.588098 .3552293 2.07 0.039 1.024421 | 2.461932 |
| 16 1.600108 .3593342 2.09 0.036 1.030376 | 2.484863 |
| 17 1.658161 .3758998 2.23 0.026 1.063315 | 2.585779 |
| 18 1.342826 .3034783 1.30 0.192 .86228 | 2.091178 |
| 19 1.598451 .3449826 2.17 0.030 1.047105 | 2.440103 |
| 20 1.624444 .3739297 2.11 0.035 1.034587 | 2.550599 |
| 21 1.163498 .2404226 0.73 0.464 .7760245 | 1.74444 |
| 22 1.447632 .3015259 1.78 0.076 .9624148 | 2.17748 |
| 23 1.537522 .3223712 2.05 0.040 1.019414 | 2.318955 |
| 24 1.477282 .3100903 1.86 0.063 .9790191 | 2.229131 |
| 25 1.648761 .3423014 2.41 0.016 1.097587 | 2.476719 |
| 26 1.626819 .3558658 2.22 0.026 1.059593 | 2.497693 |
| 27 1.427928 .3113145 1.63 0.102 .9313841 | 2.189192 |
| 28 1.547185 .3724239 1.81 0.070 .9652707 | 2.479908 |
| 29 1.34183 .3082682 1.28 0.201 .8553522 | 2.10499 |
| 30 1.405791 .2862297 1.67 0.094 .9432128 | 2.095231 |
| 31 1.467319 .306138 1.84 0.066 .9748365 | 2.208601 |
| 32 1.816251 .380315 2.85 0.004 1.204864 | 2.737876 |
| 33 1.436458 .3162012 1.65 0.100 .9330859 | 2.211384 |
| 34 1.659995 .3575767 2.35 0.019 1.088306 | 2.531994 |
| 35 1.687163 .3550708 2.49 0.013 1.11691 | 2.548568 |
| 36 1.695052 .3500966 2.56 0.011 1.130774 | 2.540917 |
| 37 1.65868 .3396467 2.47 0.013 1.110357 | 2.477779 |
| 38 .6333271 .1942604 -1.49 0.136 .3471675 | 1.155359 |
| 39 1.351088 .2807264 1.45 0.148 .89913 | 2.030227 |
| year |  |
| 2018 .982143 .0348824 -0.51 0.612 .9161001 | 1.052947 |
| 2019 1.069462 .0422113 1.70 0.089 .9898481 | 1.155478 |
| 2020 1 (omitted) |  |
| _cons .0000677 .0000138 -47.13 0.000 .0000454 | .0001009 |
| ln(popula~n) 1 (exposure) |  |
|  |  |
| /lnalpha -5.201362 1.115386 -7.387478 | -3.015245 |
|  |  |
| alpha .0055091 .0061447 .000619 | .0490338 |
|  |  |
| Note: Estimates are transformed only in the first equation. |  |
| Note: _cons estimates baseline incidence rate. |  |
| . |  |

**Table S55.- Oncologic diseases (excluding sex-specific cancer) (men, 80 years and older) (full specification)**

| Negative binomial regression Number of obs = | 156 |
| --- | --- |
| Wald chi2(43) = | 393.22 |
| Dispersion = mean Prob > chi2 = | 0.0000 |
| Log pseudolikelihood = -442.34485 Pseudo R2 = | 0.1575 |
|  |  |
| Robust |  |
| _freq IRR Std. Err. z P>z [95% Conf. | Interval] |
|  |  |
| t2 1.001802 .0005519 3.27 0.001 1.000721 | 1.002885 |
| 1.level .3419299 .0697896 -5.26 0.000 .2291937 | .5101191 |
| slope 1.023702 .0105255 2.28 0.023 1.003278 | 1.044541 |
| week |  |
| 2 1.875399 .5800636 2.03 0.042 1.022859 | 3.43852 |
| 3 1.768478 .5767124 1.75 0.080 .9332914 | 3.351059 |
| 4 1.576319 .4611319 1.56 0.120 .8884558 | 2.79674 |
| 5 1.521746 .4535945 1.41 0.159 .8484381 | 2.729381 |
| 6 1.706269 .5145333 1.77 0.076 .9448518 | 3.081281 |
| 7 1.481365 .4334835 1.34 0.179 .834795 | 2.628721 |
| 8 1.699173 .541074 1.66 0.096 .9103036 | 3.171677 |
| 9 1.254767 .3792289 0.75 0.453 .6939116 | 2.268936 |
| 10 1.64134 .49787 1.63 0.102 .9057379 | 2.974369 |
| 11 1.435296 .4281883 1.21 0.226 .7998426 | 2.575599 |
| 12 1.623799 .5326364 1.48 0.139 .8537334 | 3.088462 |
| 13 1.574273 .4747398 1.50 0.132 .8717475 | 2.842952 |
| 14 1.360235 .4134643 1.01 0.311 .7496843 | 2.468026 |
| 15 1.558336 .4953847 1.40 0.163 .8357375 | 2.905711 |
| 16 1.346302 .4294147 0.93 0.351 .7205169 | 2.515594 |
| 17 1.785685 .5221306 1.98 0.047 1.006735 | 3.167338 |
| 18 1.473465 .4321146 1.32 0.186 .8293022 | 2.617983 |
| 19 1.184505 .4361485 0.46 0.646 .5755921 | 2.437582 |
| 20 1.457633 .4767531 1.15 0.249 .7677907 | 2.767284 |
| 21 1.211337 .3523791 0.66 0.510 .6849353 | 2.1423 |
| 22 1.263852 .421522 0.70 0.483 .6573582 | 2.429911 |
| 23 1.256742 .3775175 0.76 0.447 .6975098 | 2.26434 |
| 24 1.464361 .4327089 1.29 0.197 .820585 | 2.613199 |
| 25 1.494745 .4412315 1.36 0.173 .8381127 | 2.665827 |
| 26 1.293033 .4088079 0.81 0.416 .6958121 | 2.402853 |
| 27 1.170281 .3533236 0.52 0.602 .6475909 | 2.114849 |
| 28 1.334911 .3972025 0.97 0.332 .7450366 | 2.391813 |
| 29 1.137278 .3413051 0.43 0.668 .6315604 | 2.047944 |
| 30 1.318684 .3933392 0.93 0.354 .7349248 | 2.366129 |
| 31 1.385376 .4097154 1.10 0.270 .7759449 | 2.473459 |
| 32 1.50688 .4389763 1.41 0.159 .8513556 | 2.667142 |
| 33 1.035155 .313612 0.11 0.909 .5716422 | 1.874505 |
| 34 1.322322 .4043719 0.91 0.361 .7261667 | 2.4079 |
| 35 1.532629 .480049 1.36 0.173 .8295153 | 2.831717 |
| 36 1.504359 .4567817 1.34 0.179 .8296479 | 2.72778 |
| 37 1.512272 .4564839 1.37 0.171 .8369354 | 2.732547 |
| 38 .5543104 .2380045 -1.37 0.169 .2389309 | 1.285979 |
| 39 1.651494 .4883301 1.70 0.090 .9250925 | 2.94828 |
| year |  |
| 2018 1.053896 .0498282 1.11 0.267 .9606227 | 1.156225 |
| 2019 .9931898 .0611994 -0.11 0.912 .8802014 | 1.120682 |
| 2020 1 (omitted) |  |
| _cons .000085 .0000248 -32.12 0.000 .000048 | .0001506 |
| ln(popula~n) 1 (exposure) |  |
|  |  |
| /lnalpha -18.21689 .2458144 -18.69868 | -17.7351 |
|  |  |
| alpha 1.23e-08 3.01e-09 7.57e-09 | 1.98e-08 |
|  |  |
| Note: Estimates are transformed only in the first equation. |  |
| Note: _cons estimates baseline incidence rate. |  |

**Table S56.- Oncologic diseases (excluding sex-specific cancer) (men, 80 years and older) (full specification)**

| Negative binomial regression Number of obs = | 156 |
| --- | --- |
| Wald chi2(43) = | 463.09 |
| Dispersion = mean Prob > chi2 = | 0.0000 |
| Log pseudolikelihood = -464.08227 Pseudo R2 = | 0.1793 |
|  |  |
| Robust |  |
| _freq IRR Std. Err. z P>z [95% Conf. | Interval] |
|  |  |
| t2 1.00201 .0003958 5.08 0.000 1.001234 | 1.002786 |
| 1.level .2497382 .0358363 -9.67 0.000 .188513 | .3308481 |
| slope 1.036391 .0092463 4.01 0.000 1.018426 | 1.054673 |
| week |  |
| 2 1.748995 .2887871 3.39 0.001 1.26544 | 2.417328 |
| 3 1.706033 .2619018 3.48 0.001 1.262744 | 2.304941 |
| 4 1.482771 .2507499 2.33 0.020 1.064458 | 2.065475 |
| 5 1.517809 .2455251 2.58 0.010 1.105413 | 2.084059 |
| 6 1.360204 .2635548 1.59 0.112 .9304097 | 1.988538 |
| 7 1.40815 .218483 2.21 0.027 1.038915 | 1.908614 |
| 8 1.392009 .2840566 1.62 0.105 .9331334 | 2.076539 |
| 9 1.452372 .2267468 2.39 0.017 1.069515 | 1.972282 |
| 10 1.626812 .2935752 2.70 0.007 1.142166 | 2.317105 |
| 11 1.521496 .2419403 2.64 0.008 1.114082 | 2.077899 |
| 12 1.510808 .3131581 1.99 0.047 1.006406 | 2.268011 |
| 13 1.308186 .2733537 1.29 0.199 .8685719 | 1.970305 |
| 14 1.717846 .2762164 3.37 0.001 1.253481 | 2.354241 |
| 15 1.452119 .2394655 2.26 0.024 1.051073 | 2.006189 |
| 16 1.47489 .3721629 1.54 0.124 .8994432 | 2.418497 |
| 17 1.371051 .2147082 2.02 0.044 1.008682 | 1.8636 |
| 18 1.158706 .2941955 0.58 0.562 .7044549 | 1.905871 |
| 19 1.695559 .2712073 3.30 0.001 1.239258 | 2.319871 |
| 20 1.344303 .231707 1.72 0.086 .95892 | 1.88457 |
| 21 1.243077 .2321845 1.16 0.244 .862003 | 1.792616 |
| 22 1.493497 .255048 2.35 0.019 1.068668 | 2.08721 |
| 23 1.346726 .2202905 1.82 0.069 .9773367 | 1.855729 |
| 24 1.457146 .2605034 2.11 0.035 1.026428 | 2.068606 |
| 25 1.267455 .1960619 1.53 0.125 .9359674 | 1.716344 |
| 26 1.109914 .191751 0.60 0.546 .7911044 | 1.5572 |
| 27 1.160097 .1892414 0.91 0.363 .842639 | 1.597155 |
| 28 1.282091 .2443322 1.30 0.192 .8824755 | 1.862666 |
| 29 .9972089 .1600466 -0.02 0.986 .7280696 | 1.365839 |
| 30 1.433353 .2449774 2.11 0.035 1.025351 | 2.003705 |
| 31 1.293428 .2345163 1.42 0.156 .9065831 | 1.845342 |
| 32 1.521909 .237951 2.69 0.007 1.12022 | 2.067637 |
| 33 1.271156 .2175442 1.40 0.161 .9089193 | 1.777758 |
| 34 1.35671 .2142292 1.93 0.053 .9955877 | 1.84882 |
| 35 1.508932 .2661303 2.33 0.020 1.067932 | 2.132044 |
| 36 1.291046 .1986596 1.66 0.097 .9549113 | 1.745502 |
| 37 1.238384 .1985377 1.33 0.182 .9044624 | 1.695586 |
| 38 .6531706 .2304311 -1.21 0.227 .3271391 | 1.30413 |
| 39 1.187777 .2081692 0.98 0.326 .8424689 | 1.67462 |
| year |  |
| 2018 .9926845 .0412194 -0.18 0.860 .915096 | 1.076852 |
| 2019 1.049453 .0473973 1.07 0.285 .9605493 | 1.146586 |
| 2020 1 (omitted) |  |
| _cons .0000631 9.37e-06 -65.19 0.000 .0000472 | .0000845 |
| ln(popula~n) 1 (exposure) |  |
|  |  |
| /lnalpha -18.15148 .8053597 -19.72995 | -16.573 |
|  |  |
| alpha 1.31e-08 1.05e-08 2.70e-09 | 6.35e-08 |
|  |  |
| Note: Estimates are transformed only in the first equation. |  |
| Note: _cons estimates baseline incidence rate. |  |
| . |  |
